# A single small molecule-based human embryo model reveals V-ATPase requirement in mammalian blastocyst cavitation

**DOI:** 10.1038/s41422-026-01239-3

**Published:** 2026-04-06

**Authors:** Samhan Alsolami, Arun Pandian Chandrasekaran, Yiqing Jin, Yibo Wang, Ling Zhang, Ismail M. Shakir, Yingzi Zhang, Aisha Siddique, Gerardo Ramos-Mandujano, Baolei Yuan, Maya Ayach, Alfonso Saera-Vila, Zejun Fan, Siyi Fu, Huoming Zhang, Saige Xin, Kholoud Khalid AlDakhil, Juan Carlos Izpisua Belmonte, Jin Zhang, Yang Yu, Mo Li

**Affiliations:** 1 Bioscience Program, Biomedical Sciences Division (BioMed), King Abdullah University of Science and Technology (KAUST), Thuwal, Saudi Arabia; 2 The Third Affiliated Hospital, Guangzhou Medical University, Guangzhou, Guangdong China; 3 Beijing Key Laboratory of Collaborative Innovation in Frontier Technologies for Population Quality, State Key Laboratory of Female Fertility Promotion, Beijing Advanced Center of Cellular Homeostasis and Aging-Related Diseases, Institute of Advanced Clinical Medicine, Clinical Stem Cell Research Center, Peking University Third Hospital, Beijing, China; 4 Center for Reproductive Medicine, The First Affiliated Hospital, Zhejiang University School of Medicine, Hangzhou, Zhejiang China; 5 Liangzhu Laboratory, Zhejiang University, Hangzhou, Zhejiang China; 6 KAUST Center of Excellence for Smart Health (KCSH), Thuwal, Saudi Arabia; 7 Altos Labs, San Diego, CA USA; 8 KAUST Core Labs, King Abdullah University of Science and Technology (KAUST), Thuwal, Saudi Arabia; 9 Sequentia Biotech Inc., Barcelona, Spain; 10 Bioengineering Program, Biomedical Sciences Division (BioMed), King Abdullah University of Science and Technology (KAUST), Thuwal, Saudi Arabia; 11 Cytogenetics and Molecular Cytogenetics Section, Pathology and Clinical Laboratory Medicine, Riyadh, Saudi Arabia; 12 Center for Stem Cell and Regenerative Medicine, Department of Basic Medical Sciences, and Bone Marrow Transplantation Center of the First Affiliated Hospital, Zhejiang University School of Medicine, Hangzhou, Zhejiang China; 13 Center of Gene and Cell Therapy and Genome Medicine of Zhejiang Province, Hangzhou, Zhejiang China

**Keywords:** Pluripotency, Embryonic stem cells, Stem-cell differentiation

## Abstract

Human naïve pluripotent stem cells (nPSCs) can be induced by various combinations of signaling factors to generate blastocyst-like structures, termed blastoids. Despite rapid progress in human blastoid models, their potential to uncover fundamental mechanisms of early human development remains limited, leaving key morphogenetic processes poorly understood. Here, we describe a simple and robust system in which dimethyl sulfoxide (DMSO) alone induces blastoid formation from human nPSCs. This model recapitulates key pre- and post-implantation features and exhibits enhanced polar trophectoderm (TE) organization, more efficient attachment within an implantation-relevant window, improved epiblast lumenogenesis associated with amniotic cavity formation, and more robust, sustained expansion of embryonic lineages following attachment. Using this system, we reveal a previously unrecognized mechanism underlying TE cavitation and identify lysosome-associated genes — particularly subunits of the proton pump V-ATPase — as essential regulators of blastoid cavitation. DMSO treatment upregulates key V-ATPase subunits (*ATP6V0A4* and *ATP6V1B1*), which are also enriched in the TE of human embryos. Genetic or pharmacological inhibition of V-ATPase activity disrupts lysosomal acidification, blocks intracellular vacuole formation, and impairs blastoid cavitation, whereas overexpression of V-ATPase subunits rescues this phenotype. Furthermore, genetic and pharmacological perturbations of V-ATPase function significantly compromise cavitation in both mouse and human blastocysts. Finally, DMSO treatment induces membrane biomechanical changes characteristic of early embryonic development, suggesting a mode of action distinct from conventional small-molecule, signaling pathway-based induction strategies. This simple DMSO-based blastoid model recapitulates key aspects of human blastocyst development and reveals a conserved requirement for V-ATPase-mediated lysosomal acidification during early mammalian embryogenesis.

## Introduction

Blastocyst cavitation breaks the radial symmetry of the human embryo and initiates the first axis of embryo symmetry.^1,2^ The trophectoderm (TE) plays a critical role in this process by forming a single-cell epithelial layer surrounding the inner cell mass (ICM) and regulating the movement of water to form a fluid-filled blastocoel. Understanding the mechanisms that underlie TE and blastocoel morphogenesis is crucial for modeling the formation of human blastocysts, implantation, and placental development.

In vitro derivation of human TE cells can be achieved using pluripotent stem cells (PSCs), such as naïve PSCs (nPSCs).^3^ Recently, the ability to derive TE-like cells from nPSCs has fueled the generation of many blastocyst-like models, termed blastoids.^4–10^ In vitro-derived TE cells can be produced by simultaneously inhibiting the ERK and TGFβ/Nodal pathways with PD0325901 (PD) and A83-01 (A83), respectively.^3^ Brief induction of TE in so-called PXGL nPSCs using PD and A83 (PDA83) results in the formation of human blastoids, which consist of the TE, primitive endoderm (PE, also known as the hypoblast), and epiblast (EPI).^6,8^ A multitude of other inhibitors and growth factors, including valproic acid (VPA, an HDAC inhibitor),^5,7,9,10^ CHIR99021 (a GSK3 inhibitor), BMP4,^4,5,10,11^ hEGF,^7,9^ IM-12 (a GSK3β inhibitor),^7,9^ and WH-4-023 (an LCK/SRC inhibitor)^7,9^ have been used to facilitate TE induction and/or blastoid formation. Although these external interventions are successful in inducing TE from nPSCs to different extents, they target multiple pathways that exhibit complex crosstalk, making it challenging to understand the logic of the TE program. Indeed, these targeted developmental pathways exhibit distinct activities in different blastoid protocols and between blastoids and blastocysts.^12^ Complex culture conditions involving many inhibitors and growth factors often require labor-intensive optimization to accommodate cell lines of different genetic backgrounds, and the use of supra-physiological levels of signaling manipulation has been associated with limited developmental potential.^8,13^ For example, the Rho-associated kinase (ROCK) inhibitor Y-27632 (Y) is known to suppress TE characteristics in the blastocyst^14^ by activating Hippo signaling and preventing microlumen formation preceding cavitation,^15^ but it is widely used in blastoid protocols,^4–6,10^ sometimes simultaneously with the Hippo pathway inhibitor lysophosphatidic acid (LPA).^8^ In addition, existing protocols have relied on aggregates of human PSCs for blastoid generation. Variability among cellular aggregates may hamper culture reproducibility and forward genetic screens aimed at understanding gene regulatory networks in human embryogenesis. Here, we demonstrate that a single molecule, DMSO, can induce TE from different types of human nPSCs and promote the generation of human blastoids from single nPSCs without the need for previously used inhibitors, growth factors, or genetic manipulations.^5^

We then leverage the simple, robust induction of TE cavitation by DMSO to model the first lumenogenesis event in the human embryo, which occurs on the basolateral side of polarized TE cells. Unlike well-studied apical lumenogenesis, such as formation of the amniotic cavity in post-implantation EPI,^16,17^ basolateral lumenogenesis is not well understood. An elegant study described the phenomenon in which hydraulic fracturing of cell–cell contacts in the late-morula mouse embryo forms numerous microlumens that coalesce into the blastocoel.^15^ However, the molecular and cellular mechanisms that regulate the formation of microlumens and basolateral lumenogenesis remain unclear. Answering these fundamental questions using human embryos is even more challenging and requires a tractable and reproducible model. The DMSO model enables live-cell tracking of the dynamics of microlumen formation and the intracellular vacuolar system in humans, revealing the requirement for vesicular acidification by V-ATPases in TE cavitation. Finally, we employed genetic and pharmacological manipulations and gene expression profiling across the DMSO blastoid model, mouse blastocysts, and human blastocysts to demonstrate the evolutionarily conserved requirement for V-ATPase family genes in blastocyst cavitation.

## Results

### DMSO promotes TE differentiation of human naïve PSCs

The molecular and cellular basis of the first lineage segregation event in the blastocyst, in which totipotent blastomeres of the morula differentiate into the TE and ICM lineages, remains elusive. Hippo pathway effectors,^18–20^ GATA transcription factors (TFs),^3^ and hydraulic fracturing of cellular contacts^15^ have been proposed to underlie TE lineage specification in the blastocyst. Cell surface fluctuations have been shown to regulate early embryonic lineage sorting.^21^ DMSO is known to downregulate pluripotency genes, promoting differentiation in human primed embryonic stem cells (ESCs).^22–24^ Its effects on plasma membrane fluidity and permeability are well documented and are thought to induce cellular differentiation and fusion.^25–29^ We hypothesized that DMSO could bias nPSCs toward the TE branch of the first cell fate bifurcation.

**Fig. 1:**
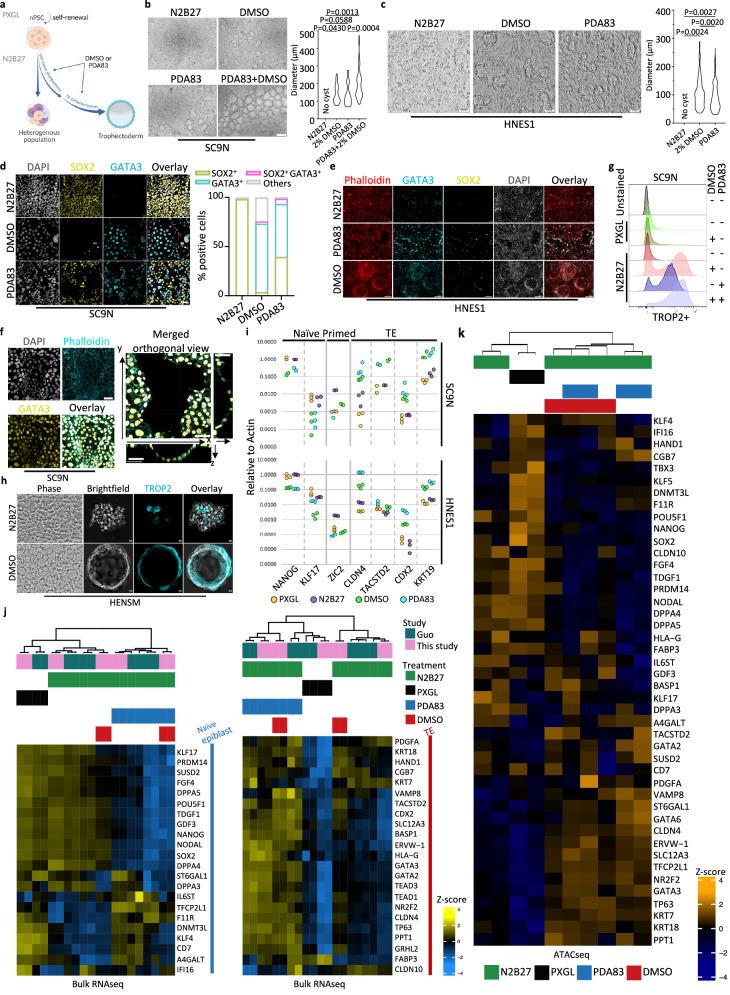
DMSO drives TE differentiation. **a** Schematic depicting the function of DMSO in promoting TE fate. **b** Representative brightfield images of day 4 structures, showing the effects of N2B27, 2% DMSO, and PDA83 conditions on cyst formation in SC9N. Scale bar, 400 µm. The violin plot shows cyst diameters. The width of each violin represents the frequency of cysts at a given diameter (right). N2B27: no cysts; 2% DMSO: *n* = 26 cysts; PDA83: *n* = 31 cysts; PDA83 + 2% DMSO: *n* = 84 cysts. One-way ANOVA followed by Tukey’s post hoc test was used, and *P* values are as indicated. **c** Representative brightfield images of day 4 structures, showing the effects of N2B27, 2% DMSO, and PDA83 conditions on cyst formation in the HNES1 cell line. Scale bars, 100 µm. The violin plot shows cyst diameters. The width of each violin represents the frequency of cysts at a given diameter (right). N2B27: no cysts; 2% DMSO: *n* = 409 cysts; and PDA83: *n* = 199 cysts. One-way ANOVA followed by Tukey’s post hoc test was used, and the *P* values are as indicated. **d** Immunofluorescence analysis showing the expression of SOX2 (yellow) and GATA3 (cyan) under N2B27, 2% DMSO, and PDA83 conditions (left) and its quantification (right) (*n* = 2). Scale bar, 40 µm. **e** Immunofluorescence analysis showing the expression of phalloidin (red), GATA3 (cyan), and SOX2 (yellow) under N2B27, PDA83, and 2% DMSO conditions in the HNES1 cell line (*n* = 2). Scale bars, 50 µm. **f** Immunofluorescence analysis showing the expression of phalloidin (cyan) and GATA3 (yellow) under 2% DMSO conditions (left) and an orthogonal view demonstrating 2D TE sphere formation (right). Scale bar, 50 μm. **g** FACS histogram showing TROP2 expression under various conditions (*n* = 3). **h** HENSM naïve cells on day 3 showing TROP2 expression (cyan). **i** qRT-PCR showing the expression levels of various marker genes of the naïve, primed, and TE states under different culture conditions in SC9N (top; *n* = 2) and HNES1 (bottom; *n* = 3) cell lines. **j**, **k** Heatmaps showing the differential expression patterns of genes under different treatments from the bulk RNA-seq (**j**) and ATAC-seq (**k**) analyses.

To test this hypothesis, we exposed PXGL nPSCs (hereafter referred to as nPSCs, unless otherwise specified) to DMSO in N2B27 basal medium (N2B27) in two-dimensional (2D) culture (Fig. 1a). Intriguingly, within 3–4 days, DMSO-treated nPSCs consistently differentiated into numerous cystic structures, a process reminiscent of the TE induction by PDA83 reported previously.^3^ DMSO further boosted the effect of PDA83 on epithelial cyst formation, resulting in a confluent layer of fluid-filled cysts in SC9N (an iPSC-reverted nPSC line; Fig. 1b) and HNES1 (an embryonic nPSC line; Fig. 1c). The highly reproducible (> 15 trials) formation of cystic structures suggested that DMSO promoted differentiation of functional nPSC-derived TE-like cells. Consistent with this notion, DMSO treatment downregulated the pluripotency marker SOX2 and activated the key TE TF GATA3 in the majority of cells in SC9N (Fig. 1d; Supplementary information, Fig. S1a) and HNES1 cell lines (Fig. 1e). Three-dimensional (3D) imaging reconstruction showed that the lining of the cysts consisted of a single layer of cells with strong nuclear GATA3 signals and prominent cortical F-actin (stained by phalloidin) at cell–cell boundaries, consistent with a polarized TE-like epithelium in both nPSC lines (Fig. 1f; Supplementary information, Fig. S1b, c). Flow cytometry revealed that DMSO upregulated the TE marker TROP2 (encoded by the *TACSTD2* gene) more potently than PDA83 and acted additively with PDA83 to promote the TE fate in most cells (Fig. 1g; Supplementary information, Fig. S1d–g). Similarly, DMSO potently induced TROP2^+^ TE trophospheres in nPSCs maintained under human enhanced naive stem cell medium (HENSM) conditions,^11,30^ demonstrating that its TE induction effect is consistent across different nPSC culture conditions (Fig. 1h). Gene expression analysis by quantitative reverse transcription PCR (qRT-PCR) confirmed the sharp downregulation of naïve pluripotency genes and the upregulation of TE marker genes in DMSO-treated cells, without upregulation of primed pluripotency markers, in SC9N and HNES1 cell lines (Fig. 1i).

To gain a comprehensive view of the DMSO-induced transcriptional program, we performed RNA sequencing (RNA-seq) of nPSCs in PXGL and various differentiation conditions. Principal component analysis (PCA) showed good reproducibility and agreement with published datasets^3^ and revealed that DMSO-treated samples clustered closer to TE-differentiated cells (Supplementary information, Fig. S1h). DMSO, with or without PDA83, upregulated TE markers (e.g., *TACSTD2*, *GRHL2*, *TEAD1*, *CDX2*, *KRT18*, *KRT19*, *CLDN4*, and *CGB7*) and downregulated naïve pluripotency genes (e.g., *NANOG*, *KLF17*, *KLF4*, *DNMT3L*, and *SUSD2*) (Fig. 1j; Supplementary information, Fig. S1i). Differentially expressed genes (DEGs) between the DMSO and N2B27 conditions were significantly enriched in Gene Ontology (GO) terms consistent with TE cell fate (e.g., placenta, basolateral plasma membrane, and Hippo signaling) (Supplementary information, Fig. S1j). Together, these data suggested that a single small molecule, DMSO, could induce differentiation of TE-like cells from nPSCs without the need for other factors.

### DMSO directs changes in chromatin accessibility that promote TE differentiation

To further understand the *cis*-regulatory elements involved in DMSO-induced transcriptomic changes, we performed an assay for transposase-accessible chromatin with sequencing (ATAC-seq). The chromatin accessibility landscape of DMSO-treated samples clustered close to that of PDA83-differentiated TE cells and was distinct from that resulting from the simple removal of naïve culture conditions (N2B27) (Supplementary information, Fig. S2a). DMSO increased chromatin accessibility in the neighborhood of TE genes, a chromatin dynamics profile shared with PDA83 (Fig. 1k; Supplementary information, Fig. S2b). Notably, DMSO led to diminished ATAC-seq peaks for many naïve and primed pluripotency markers, reminiscent of PDA83 and corroborated by changes in gene expression (Supplementary information, Figs. S2b and S1i, j).

Motif enrichment analysis showed that DMSO was associated with significantly higher activation scores^31^ of TE TFs (e.g., *GATA2*, *GATA3*, *TEAD2*, and *TEAD3*) and lower activation scores of naïve TFs (e.g., *POU5F1*, *SOX2*, and *DUX*) (Supplementary information, Fig. S2c, d). Finally, DMSO-induced TE-like cells could be further differentiated into syncytin-1/ERVW-1-positive syncytiotrophoblasts (STBs) and HLA-G-positive extravillous trophoblasts (EVTs) following established protocols^4,32^ (Supplementary information, Fig. S2e). Together, these data demonstrate that DMSO alone can induce gene expression and *cis*-regulatory programs consistent with TE differentiation from nPSCs.

### DMSO enhances pre- and peri-implantation blastoid formation

Since DMSO dramatically enhanced TE differentiation (Fig. 1), we hypothesized that it could facilitate human blastoid formation through a method^8^ that combines PDA83, LPA, LIF, and Y (referred to as PALLY). We first optimized the timing and dosage of DMSO treatment in the PALLY protocol (Supplementary information, Fig. S3a) and found that DMSO significantly promoted PALLY-induced cavitation. The average blastoid diameter increased from 0.5% to 2% with increasing DMSO dosage (Supplementary information, Fig. S3a, right panel), whereas 0.1% DMSO had little effect (data not shown). We therefore decided to use 0.5% DMSO, which produced diameters consistent with those of high-quality human blastocysts 5–7 days after fertilization,^33^ while consistently producing cavitated structures (Supplementary information, Fig. S3b, c). The cavitated structures in PALLY + DMSO were morphologically indistinguishable from PALLY blastoids, consisted of properly arranged TE-, EPI-, and PE-like cells, and underwent the same morphogenic processes of compaction, cavitation, and expansion (Supplementary information, Fig. S3d and Videos S1, S2), thereby demonstrating their blastoid identity. Importantly, DMSO significantly improved cavitation efficiency across different nPSC lines and nPSCs of different passages, which otherwise exhibited substantial variability in blastoid formation (Supplementary information, Fig. S3e). Current blastoid models^4–8,10,34^ have not demonstrated derivation from single cells. To test clonal blastoid generation, we seeded single human nPSCs in microwells using limiting dilution (Supplementary information, Fig. S3f). Single nPSCs were cultured in N2B27 for 6–8 days, followed by PALLY and LY treatments (Supplementary information, Fig. S3g). PALLY induced cavitated structures with all three lineages (Supplementary information, Fig. S3g–i). Addition of DMSO further enhanced cavitation efficiency (Supplementary information, Fig. S3i) and tri-lineage formation (Supplementary information, Fig. S3j), confirming its role in reliable blastoid generation under PALLY conditions.

Single-cell RNA-seq (scRNA-seq) revealed that blastoids from both PALLY + DMSO and PALLY conditions possessed well-delineated cellular populations expressing marker genes^3,8,35^ specific to the three lineages of the blastocyst (Supplementary information, Fig. S3k–n). The transcriptomic profiles of cells from PALLY + DMSO blastoids aligned well with those from human blastocysts^35^ and PALLY blastoids (from this study and a published study^8^) but not with human embryos prior to lineage segregation. This is consistent with the expected developmental stage of the blastoids (Supplementary information, Fig. S3k, m). PSCs (OCT4^+^NANOG^+^) and trophoblast stem cells (GATA3^+^CK7^+^) were successfully re-derived from PALLY + DMSO and PALLY blastoids as described previously^8^ (Supplementary information, Fig. S3o).

After expansion and hatching, the blastocyst implants into the endometrium via the TE, which gives rise to cytotrophoblasts (CTBs), STBs, and EVTs. To model peri-implantation, blastoids were cultured for 4 days on dishes pre-coated with Matrigel (see Materials and Methods). PALLY + DMSO blastoids first collapsed, and the outer TE invaded the matrix, while the inner cells flattened into a disc-like structure (Supplementary information, Fig. S4a). The TE derivatives (CK18^+^GATA3^+^phalloidin^+^) spread outward and secreted human chorionic gonadotropin (hCG) at levels detectable by commercial pregnancy tests (Supplementary information, Fig. S4b, c). Post-implantation EPI undergoes epithelialization and another cavitation event to form the amnion and the amniotic cavity.^16^ Interestingly, the disc-like structure contained well-packed SOX2^+^ EPI analogs, with strong phalloidin and PODXL staining between cells at the periphery of the colony, suggesting the formation of an early amniotic cavity-like structure. By contrast, PODXL^+^ signals were found only sparsely in attached PALLY blastoids (Supplementary information, Fig. S4c). GATA4^+^ PE derivatives were also present near the periphery of the SOX2^+^ cell clusters (Supplementary information, Fig. S4c).

scRNA-seq revealed that the transcriptomes of cells in attached blastoids aligned well with those of cells in human post-implantation embryos^36–38^ but not with those in pre-implantation blastocysts,^35^ as expected from their developmental stage (Supplementary information, Fig. S4d, e). Attached PALLY + DMSO and PALLY blastoids contained well-delineated cellular populations expressing marker genes^3,8,35^ specific to STBs (e.g., *CGB2*, *CGB5*, and *ERVFRD-1*), CTBs (e.g., *FABP5*, *ITGA6*, and *FOXH1*), and EVTs (e.g., *HLA-G*, *RXRA*, and *TFDP2*) (Supplementary information, Fig. S4f). Together, these observations demonstrate that DMSO facilitates the efficient formation of blastoids that can differentiate into lineages analogous to those found in post-implantation embryos.

### DMSO alone induces blastoids without other lineage-instructive cues

Considering that DMSO promotes TE differentiation and improves the efficiency of PALLY blastoid formation (Fig. 1; Supplementary information, Figs. S1–S3), we tested whether DMSO alone is sufficient to induce blastoid formation. To test this hypothesis, we first treated nPSCs with 2% DMSO in N2B27 during a 2-day window. By day 6, blastocyst-like cavitated structures emerged, although at low efficiency (Supplementary information, Fig. S5a–c) and without a detectable PE lineage (data not shown). However, extending the culture to day 7 confirmed the presence of all three lineages (Supplementary information, Fig. S5d–h). We next tested whether LIF and LPA, both shown to enhance human blastoid development,^8,39^ could improve the efficiency of DMSO-only blastoid formation and support the PE lineage. Supplementing DMSO treatment with LIF, LPA, or both enhanced cavitation efficiency and PE lineage formation, particularly when LPA was added at later stages (conditions #1, #5, and #7), without affecting blastoid size (Supplementary information, Fig. S5i–l). LPA facilitated the formation of all three lineages, whereas LIF was dispensable (Supplementary information, Fig. S5m–o), indicating that Hippo inhibition enhances DMSO-induced blastoid generation.

**Fig. 2:**
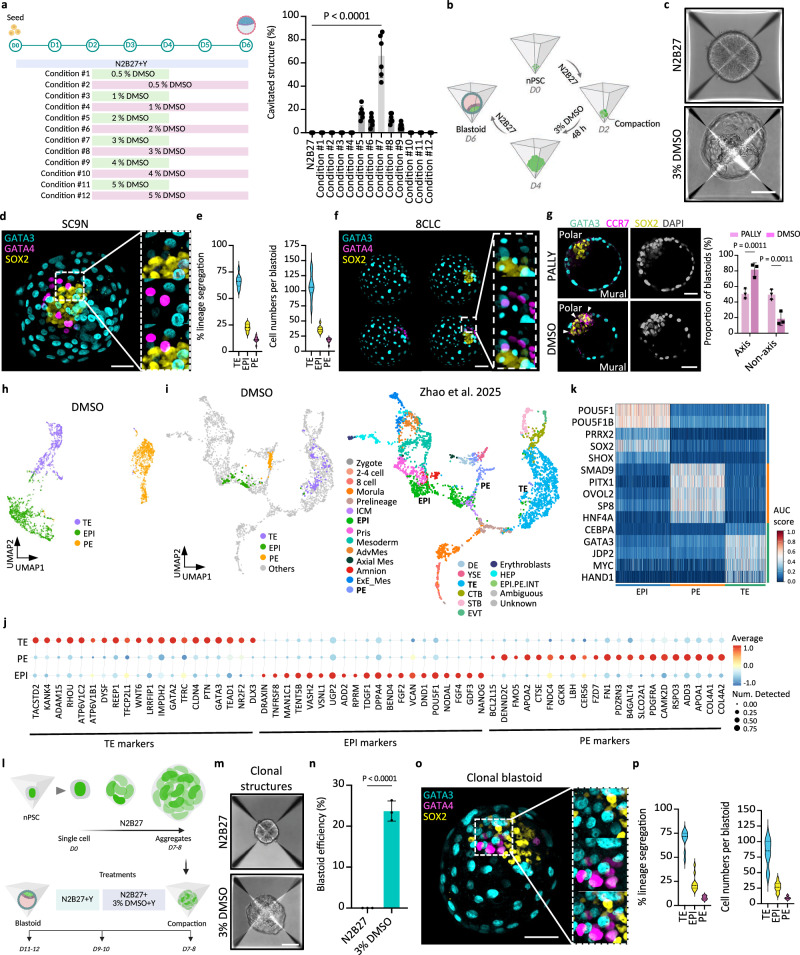
Derivation of human blastocyst-like structures using only DMSO. **a** Schematic showing the DMSO optimization strategy for generation of blastocyst-like structures (left) and a graph showing the percentage of cavitated structures under various DMSO conditions (right). Data are presented as mean ± standard deviation. One-way ANOVA followed by Dunnett’s post hoc test was used, and *P* values are as indicated (*n* = 3 for conditions #1–4 and #10–12; *n* = 6 for conditions #5–9). **b** Schematic showing the generation of blastoids using DMSO alone. **c** Representative phase-contrast images of N2B27- and 3% DMSO-treated structures harvested from the microwells on day 6 (*n* = 3). Scale bar, 100 µm. **d** Immunofluorescence analysis of a TE marker (GATA3; cyan), EPI marker (SOX2; yellow), and PE marker (GATA4; magenta) on a 3% DMSO-derived blastoid generated from PXGL nPSCs (*n* = 3). Scale bar, 50 µm. **e** Graphs showing the percentage lineage segregation (left) and cell number distribution (right) of 3% DMSO blastoids (*n* = 10). One-way ANOVA was used for analysis. **f** Immunofluorescence analysis of a TE marker (GATA3; cyan), EPI marker (SOX2; yellow), and PE marker (GATA4; magenta) on 3% DMSO-derived blastoids generated from 8CLC cells (*n* = 3). Scale bar, 50 µm. **g** Immunofluorescence analysis of a polar TE marker (CCR7; magenta), EPI marker (SOX2; yellow), and TE marker (GATA3; cyan) on 3% DMSO-derived blastoids (left). The graph shows quantification of the proportion of blastoids from PALLY and DMSO conditions with preferential CCR7 expression on the polar side (axis) compared with the non-polar side or no expression (non-axis) (*n* = 3). Scale bars, 50 μm. Two-way ANOVA was used. **P < 0.01. **h**, **i** UMAP of the transcriptome of single cells from 3% DMSO blastoids at day 6 with cell-type annotations (**h**) and projection onto a published human embryonic reference atlas (**i**). **j** Dot plots showing gene expression profiles of the three distinct clusters shown in **h**. **k** Heatmap showing average AUC values from the SCENIC analysis. **l** Schematic depicting the generation of clonal blastocyst-like structures under 3% DMSO conditions. **m** Representative phase-contrast images showing cavitated structures under 3% DMSO conditions (*n* = 3). Scale bar, 100 µm. **n** Graph showing the efficiency of cavitated clonal structures. Data are presented as mean ± standard deviation of three independent experiments. A two-tailed Student’s *t*-test was used, and the *P* value is indicated. **o** Immunofluorescence analysis of a TE marker (GATA3; cyan), EPI marker (SOX2; yellow), and PE marker (GATA4; magenta) on DMSO-only clonal blastoids (*n* = 3). Scale bar, 50 µm. **p** Graphs showing the percentage lineage segregation (left) and cell number distribution (right) of 3% DMSO clonal blastoids (*n* = 10). O*n*e-way ANOVA was used for analysis.

We noticed that several blastoid models,^5,7,8^ including the 2% DMSO model, displayed an aberrant positioning of the ICM, which extended tangentially outside the bounds of the TE (termed “tangential ICM”, Supplementary information, Fig. S6a). To address this positional misalignment, we tested various DMSO concentrations and treatment timings. Surprisingly, we found that 3% DMSO (condition #7) not only improved ICM centrality but also significantly improved the cavitation rate compared with other conditions (up to 86.67%) (Fig. 2a; Supplementary information, Fig. S6b). We therefore used 3% DMSO to induce blastocyst-like structures from nPSCs (Fig. 2b). Window-specific treatment with 3% DMSO generated blastoids that contained all three lineages with correct developmental timing (by day 6) from nPSCs derived using either the PXGL or 8-cell-like cell (8CLC) culture conditions (Fig. 2c–f; Supplementary information, Fig. S6c–e). Next, we assessed polar trophectoderm (pTE) specification by examining the spatial localization of the pTE marker CCR7.^35^ DMSO blastoids exhibited a significantly higher frequency of correctly localized CCR7^+^ cells on the polar side compared with PALLY blastoids (Fig. 2g), indicating that the simplified DMSO system supported pTE differentiation and spatial organization more effectively than the PALLY conditions.

scRNA-seq of 3% DMSO-only blastoids revealed three distinct clusters corresponding to the TE, EPI, and PE, which matched human blastocyst transcriptional profiles but differed from those of pre-lineage embryos (Fig. 2h).^8,35^ Integration with a human embryo reference atlas further demonstrated strong concordance between each blastoid lineage and its corresponding in vivo counterpart^40^ (Fig. 2i). An independent replicate of the 3% DMSO-only blastoid scRNA-seq experiment reproducibly confirmed these observations (Supplementary information, Fig. S6f).

Analysis of lineage-specific expression further highlighted canonical marker genes associated with TE, EPI, and PE identities (Fig. 2j; Supplementary information, Fig. S6g, h). To assess developmental dynamics, we performed RNA velocity and latent time analyses. RNA velocity vectors were largely confined within individual TE, EPI, and PE clusters, indicating lineage-specific transcriptional maturation (Supplementary information, Fig. S6i). Latent time analysis suggested the presence of a common progenitor state within the EPI cluster, consistent with early blastocyst developmental trajectories (Supplementary information, Fig. S6j).

We next used single-cell regulatory network inference and clustering analysis (SCENIC) to examine lineage-specific TF activity. Regulon AUC heatmaps revealed distinct, lineage-restricted regulatory programs that independently supported our lineage annotation (Fig. 2k). Specifically, the EPI compartment was enriched for pluripotency-associated regulons, with elevated *POU5F1* and *SOX2* activity; the PE compartment showed elevated activity of *OVOL2* and *SP8* regulons; and the TE compartment displayed strong activation of TE-associated regulons, including *GATA3*. These lineage-specific regulon signatures provide orthogonal, regulatory network-level validation of TE, EPI, and PE identities in DMSO blastoids, complementing marker-based expression analyses.

Overall, scRNA-seq analysis demonstrated robust lineage segregation in 3% DMSO-only blastoids that closely paralleled human blastocyst development, with transcription corresponding to the expected developmental stage. Moreover, window-specific treatment with 3% DMSO alone enabled clonal derivation of cavitated blastoids from single nPSCs encompassing all three lineages (Fig. 2l–p; Supplementary information, Fig. S6k–m), demonstrating the strong inductive potential of DMSO.

### DMSO-only blastoids model human implantation in vitro

**Fig. 3:**
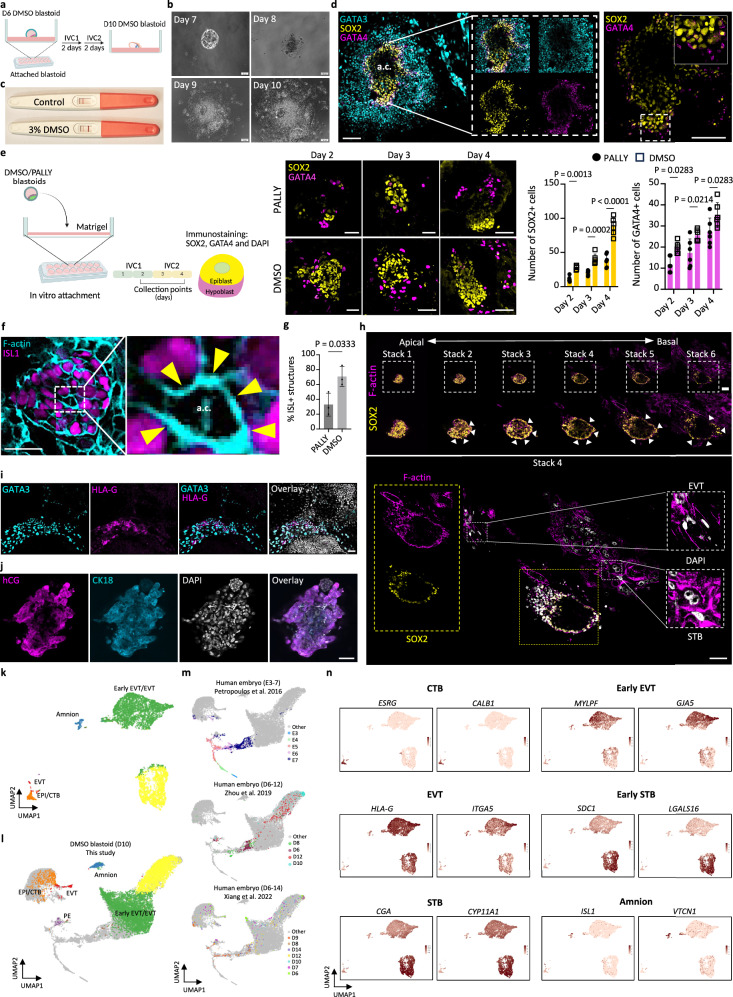
Generation of embryonic and extraembryonic-like structures from DMSO-derived blastoids. **a** Schematic showing the in vitro attachment assay to recapitulate post-implantation development from 3% DMSO-only blastoids. **b** Representative phase-contrast images of attached DMSO-only blastoids (*n* = 3). Scale bars, 100 µm. **c** Detection of secreted hCGβ in the medium of attached DMSO blastoids using a commercial pregnancy test kit (*n* = 1). **d** Immunofluorescence images showing the expression of GATA3, SOX2, and GATA4 in 3% DMSO-attached blastoids (*n* = 3). Scale bar, 50 µm. Dotted areas indicate formation of the amniotic cavity (a.c.). **e** Schematic showing the protocol used to study epiblast development. Samples were collected on days 2, 3, and 4 for immunofluorescence analysis using the indicated antibodies. Time-resolved immunostaining analysis shows SOX2 and GATA4 expression in attached blastoids. Scale bars, 50 μm. Graphs show the percentage of EPI and hypoblast areas in PALLY- and DMSO-attached blastoids (*n* = 6). Two-way ANOVA followed by Tukey’s post hoc test was used. *P* values are as indicated. **f** Immunofluorescence images showing the expression of F-actin and ISL1 in attached 3% DMSO blastoids (*n* = 3). Scale bar, 50 µm. Yellow arrows highlight formation of the a.c.. **g** Graph showing the percentage of ISL1-positive structures under PALLY and DMSO conditions. Data are presented as the mean and standard deviation of three independent experiments. Student’s *t*-test was used, and the *P* value is as indicated. **h**–**j** Immunofluorescence images showing the expression of SOX2, F-actin, GATA3, HLA-G, CK18, and hCG in attached 3% DMSO blastoids (*n* = 3). Scale bar, 50 µm. White arrows in **h** indicate formation of the a.c. **k** UMAP of single-cell transcriptomes from day 10 3% DMSO blastoids with cell-type annotations. **l** Same as in **k**, shown as an integrated dataset projection. **m** UMAP projections of integrated datasets showing cells from this study alongside those from previously published reports. **n** Feature plots of markers of TE subtypes (CTB, early EVT/EVT, and early STB/STB) and amnion under 3% DMSO conditions.

To evaluate the ability of DMSO-only blastoids to recapitulate human implantation, we performed an established in vitro attachment assay^17^ (Fig. 3a). The majority of 3% DMSO-only blastoids attached within 24 h and developed outgrowth morphologies resembling those of human blastocysts (Fig. 3b). By day 4 post attachment, hCG was detectable (Fig. 3c). Human blastocysts at 6–7 days post fertilization attach within 24 h under comparable in vitro conditions.^41,42^ Given that DMSO blastoids displayed enhanced polar TE differentiation relative to the PALLY conditions (Fig. 2g), we directly compared attachment efficiency between the two systems within this time window. DMSO blastoids exhibited significantly higher attachment rates than PALLY blastoids (Supplementary information, Fig. S7a).

After 4 days of attachment, GATA3^+^ TE cells expanded outward, whereas SOX2^+^ EPI cells were surrounded by GATA4^+^/GATA6^+^ PE cells, with clear spatial segregation among these lineages (Fig. 3d; Supplementary information, Fig. S7b).

Because sustained embryonic lineage expansion is a defining feature of early post-implantation human embryo development,^43^ we next compared post-attachment growth kinetics between DMSO- and PALLY-derived blastoids. Immunofluorescence analyses over 3 consecutive days following attachment revealed progressive increases in both EPI (SOX2^+^) and PE (GATA4^+^) populations under both conditions (Fig. 3e). However, DMSO blastoids exhibited significantly greater and more sustained EPI and PE expansion throughout this period compared with PALLY blastoids (Fig. 3e), indicating improved post-attachment growth and maturation.

In vitro-attached human blastocysts are known to undergo EPI expansion, polarization, and amniotic cavity formation.^17,44^ Consistent with this process, attached DMSO blastoids developed a prominent central lumen surrounded by radially organized SOX2^+^ EPI cells enriched for F-actin and PODXL, a marker of EPI lumenogenesis (Fig. 3f; Supplementary information, Fig. S7c). Expression of the amnion marker ISL1 further confirmed the presence of amnion-like cells, which displayed characteristic apical F-actin accumulation (Fig. 3f). Quantitatively, the DMSO condition yielded a significantly higher proportion of blastoids containing ISL1^+^ cells compared with the PALLY condition (Fig. 3g; Supplementary information, Fig. S7d).

We next identified STB- and EVT-like cells at the perimeter of the attached DMSO-only blastoids, which were characterized by multi-nucleated and spindle-shaped morphologies, respectively (Fig. 3h). Unlike other blastoid models that displayed either an absence or delayed development of EVT-like cells,^5,34,45^ DMSO-only blastoids gave rise to HLA-G^+^ EVT-like cells by day 10 (Fig. 3i). We also identified the presence of hCG^+^ STB-like cells (Fig. 3j). Together, these observations demonstrate robust recapitulation of key human peri-implantation events in the DMSO-only blastoid model (Supplementary information, Fig. S7e).

To further characterize post-implantation cell states, we performed scRNA-seq of day 10 (D10) DMSO blastoids, which revealed distinct clusters (Fig. 3k) that aligned closely with post-implantation human embryo reference datasets (Fig. 3l, m; Supplementary information, Fig. S7f). Further analysis identified well-defined, differentiated TE populations within both the D10 DMSO blastoid and integrated datasets, characterized by the expression of marker genes specific to early CTB (*ESRG*, *CALB1*, *ALPL*, *RAB3B*, and *AKAP12*), early EVT (*MYLPF*, *GJA5*, *ERVMER34-1*, *LINC00643*, *LGALS3*, and *CLMP*), EVT (*HLA-G*, *ITGA5*, *COL4A2*, *COL4A1*, *FAM114A1*, and *LIFR*), early STB (*SDC1*, *LGALS16*, *PRR9*, *LYPD3*, *SLC40A1*, *HOPX*, and *GADD45G*), and STB (*CGA*, *CYP11A1*, *NEURL1*, *FDX1*, and *S100P*) (Fig. 3n; Supplementary information, Fig. S7g). Consistent with our immunofluorescence findings in D10 DMSO blastoids (Fig. 3d, f, h; Supplementary information, Fig. S7b, c), we identified a cluster of amnion-like cells that exhibited high expression of amnion markers, including *ISL1* and *VTCN1* (Fig. 3n). No differences in the expression of apoptosis-related genes were detected between the PALLY and DMSO blastoids (D6 and D10), indicating that window-specific DMSO treatment did not cause overt cytotoxicity (Supplementary information, Fig. S7h–k). Together, these findings show that DMSO-only blastoids robustly recapitulate key features of post-implantation development.

### Signaling requirements of DMSO-induced cavitation

DMSO has a wide range of bioactive properties, including acting as a cryoprotectant,^46^ a solvent and penetration enhancer,^47^ and a potent inducer of cellular differentiation.^25–29^ However, its mode of action at the molecular level remains unclear. We demonstrated that DMSO significantly promotes cavitation and subsequent blastoid development of nPSCs. Cavitation, a critical event in embryogenesis, requires the differentiation of a polarized fluid-tight TE epithelium, which is actuated by Hippo signaling pathway effectors and TE-specific TFs in humans.^3,18,20^ Since atypical PKC (aPKC) initiates apical-basal polarity in morula-stage embryos,^19^ we hypothesized that it might play a role in DMSO-induced cavitation. aPKC was initially expressed at day 3 and later became restricted to GATA3^+^ TE cells, but not OCT4^+^ ICM cells, under both DMSO and PDA83 conditions (Supplementary information, Fig. S8a, b, d). This was accompanied by the upregulation of associated polarity genes (e.g., *AMOT*, *GRHL2*, *PARD6A/B*, *RAB25*, and *YAP1*) (Supplementary information, Fig. S8c). Blocking PKC activity using Gö6983 abolished epithelial cyst formation under both DMSO and PDA83 conditions and reduced GATA3 and YAP1 expression without affecting OCT4 levels (Supplementary information, Fig. S8e, f), consistent with previous findings in human blastoids.^7^ Inhibition of specific PKC isozymes showed that PKCη and PKCζ are critical for cavitation (Supplementary information, Fig. S8g, h), indicating that PKC signaling is essential for DMSO-induced blastoid formation.

Retinoic acid (RA), like DMSO, is a known differentiation inducer^48^ involved in early embryogenesis.^49,50^ We therefore tested whether RA signaling mediated the effects of DMSO. Although RA receptor motifs were enriched in DMSO samples (Supplementary information, Fig. S2d), RA itself failed to induce cysts or TE-like cells, and RA inhibitors did not block DMSO-induced effects (Supplementary information, Fig. S9a, b), suggesting that RA signaling is not involved in DMSO-induced cavitation. We also found that DMSO downregulated cell cycle genes (Supplementary information, Fig. S9c), as reported in other studies,^51,52^ and caused G1-phase accumulation (Supplementary information, Fig. S9d). DMSO treatment significantly reduced the fraction of phosphorylated Rb (pRb-Ser480) required for G1/S transition^53^ (Supplementary information, Fig. S9e), which coincided with TE marker expression and cyst formation (Supplementary information, Fig. S1a–g). Thus, DMSO may promote TE differentiation by modulating cell cycle progression, as observed in the DMSO-induced differentiation of primed PSCs.^52^

### The lysosomal pathway underlies DMSO-induced basolateral lumenogenesis and cavitation

The development of a water-tight TE epithelium leads to the formation of a fluid-filled cavity, the blastocoel, that positions the ICM to one side of the blastocyst, thereby initiating the first axis of embryo symmetry.^1,2^ The blastocoel forms through basolateral lumenogenesis initiated by the hydraulic fracturing of cell–cell contacts into microlumens in late morulae.^15^ However, the molecular and cellular events that underlie microlumen formation and basolateral lumenogenesis remain unknown. Our results strongly establish DMSO as a simple and robust inducer of TE cavitation in 2D and 3D cultures across different nPSC culture conditions, thus offering a good system to model the first lumenogenesis event in the human embryo.

**Fig. 4:**
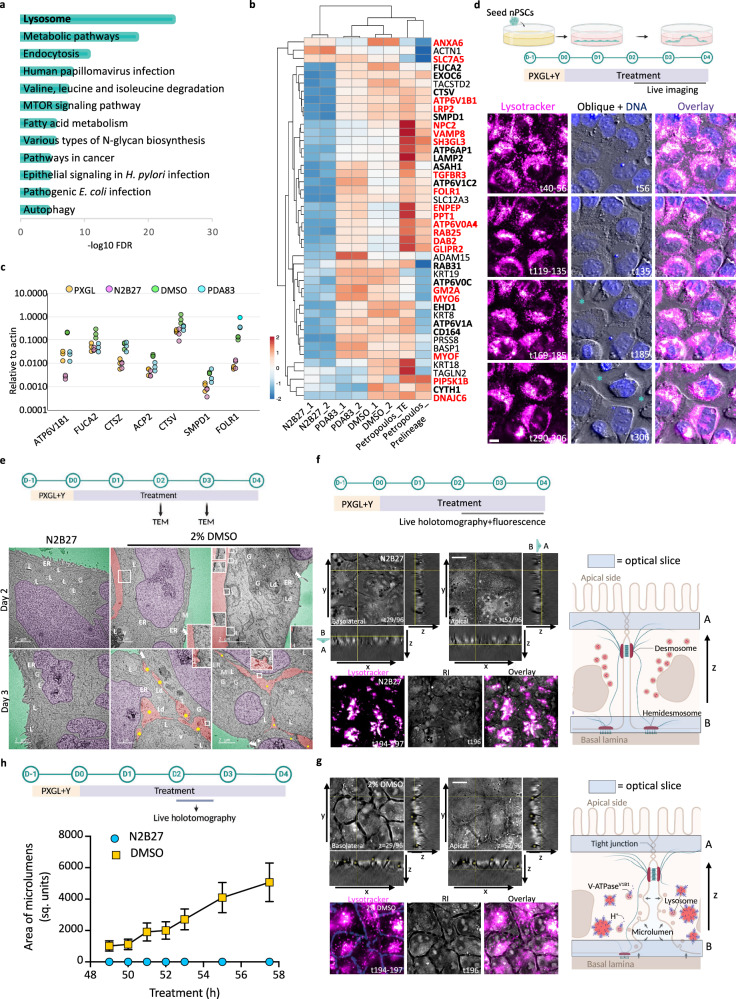
The lysosome pathway underlies DMSO-induced basolateral lumenogenesis and cavitation. **a** KEGG pathway enrichment analysis of differentially upregulated genes between the DMSO and N2B27 conditions. **b** Heatmap of lysosome pathway gene expression in DMSO, PDA83, N2B27, and human preimplantation blastocysts (log_10_ transformed gene expression values). **c** qRT-PCR analysis of the expression patterns of lysosome-related genes under different culture conditions (*n* = 3). **d** Live-cell fluorescence and oblique illumination microscopy images of 2% DMSO differentiation cultures (*n* = 2). LysoTracker (magenta) and Hoechst 33342 (blue) were added on day 2. Images were acquired every 2 min. Time points indicate the number of minutes since imaging began. Cyan asterisk: TE cyst. Scale bar, 10 μm. **e** TEM images of cells under different treatment conditions on days 2 and 3 of the differentiation experiment illustrated in the schematic above. L lysosome, ER endoplasmic reticulum, G Golgi, Ld lipid droplet, M mitochondria, V intracellular vacuole. Yellow asterisk: microlumen; solid arrowhead: tight junction; open arrowhead: desmosome. Red shading indicates the basolateral side, green indicates the apical side, and purple indicates the nucleus. Insets show endocytic cups on the basolateral membrane. Scale bars, 2 μm. **f**, **g** 3D live holotomography images showing the formation of microlumens under N2B27 (**f**) and 2% DMSO (**g**) conditions (*n* = 3). LysoTracker (magenta) was added immediately before imaging to track lysosomes. Cell drawings next to the x-z and y-z views indicate the apical (A) and basal (B) orientation of the cells. Blue masks in the lower left (**g**) indicate microlumens. Yellow asterisk: microlumen. Scale bar, 10 μm. **h** Graph showing microlumen area under N2B27 (blue) and 2% DMSO (yellow) conditions. Data are presented as the mean and standard deviation of three independent experiments. Two-way ANOVA was used for analysis.

Surprisingly, endocytic-lysosomal pathway genes were strongly enriched among those specifically upregulated in nPSCs treated with DMSO (Fig. 4a). Intriguingly, most of these endocytic-lysosomal genes were also specifically upregulated in pre-lineage and TE lineage cells in a published scRNA-seq dataset^35^ of human preimplantation embryos, suggesting their in vivo relevance (Fig. 4b). qRT-PCR confirmed the expression of selected genes and revealed a consistent gene expression pattern under PDA83 conditions (Fig. 4b, c). The list included well-documented TE marker genes (e.g., *PPT1*,^6,35^ *VAMP8*,^6,35^ *LRP2*,^6,35^ and *DAB2*^6,35^) and many genes encoding subunits of the vacuolar-type ATPase (V-ATPase), whose role in the TE remains unexplored (Fig. 4b; Supplementary information, Fig. S10a). Given that V-ATPases acidify intracellular vesicles and are essential for lysosomal function,^54^ their enrichment suggests a potential, previously unrecognized role of lysosomal activity in TE biology.

To explore the connection between lysosomal activity and TE morphogenesis, we performed live-cell imaging of lysosome dynamics and cell morphology in the 2D TE differentiation system described in Fig. 1 and previously.^3^ Beginning at day 2 of DMSO-induced TE cavitation, the inconspicuous cell–cell boundary lines under oblique illumination started to become reorganized into 3–5-µm-wide bands with numerous micron-sized blebs (mean diameter, 1.62 ± 0.3 μm (SD)), seemingly due to membrane detachment from the basal lamina (Fig. 4d, see t135; Supplementary information, Fig. S10b). The blebs formed dynamically and gradually merged into continuous basolateral “gulfs” a few microns wide between apposing cells, which grew in size over time, leading to delamination of neighboring cells to form fluid-filled cysts (Fig. 4d, t56-306; Supplementary information, Fig. S10b, c). These observations were reminiscent of hydraulic fracturing and coarsening of microlumens in the mouse blastocyst,^15^ suggesting that DMSO-induced cavitation could serve as a model for blastocoel formation. Simultaneous live-cell imaging of lysosomes using LysoTracker Red showed that smaller lysosomes frequently formed within cell–cell boundaries with blebs, whereas larger lysosomes were more centrally located near the nucleus. These observations were consistent with trafficking and maturation of lysosomes and suggested a role for lysosomes in the reorganization of basolateral cellular adhesion (Fig. 4d; Supplementary information, Fig. S10b, c). Consistent with our results, previous reports^55,56^ in mouse found abundant basally located lysosomes in TE but not ICM cells.

To better characterize the location and properties of intracellular vesicles, including lysosomes, we performed an ultrastructural analysis using transmission electron microscopy (TEM). Consistent with the live-cell imaging results, cells differentiated with DMSO accumulated endocytic vesicles and lysosomes on their basolateral side, where the Golgi apparatus was also preferentially located (Fig. 4e). DMSO-differentiated TE cells frequently contained large intracellular vacuoles several microns in diameter that contained secondary lysosomes (likely through fusion, see Supplementary information, Video S3) and granular contents consistent with the polysaccharidic polygranules described in intracellular vacuoles of TE cells in the mouse nascent blastocyst.^56^ Live-cell imaging confirmed that DMSO promoted the endocytosis of dextrans conjugated with pH-sensitive fluorogenic probes, which accumulated in large acidic intracellular vacuoles observed by TEM (Supplementary information, Fig. S10d).

### Functional requirement for V-ATPase in human blastoid cavitation

The V-ATPase complex, typically located on the lysosomal membrane and specifically upregulated in TE cells of the human embryo as well as during DMSO differentiation (Fig. 4b, c; Supplementary information, Fig. S10a), showed strong staining in vesicles of various sizes, including large vacuoles, suggesting its role in the acidification of these compartments (Supplementary information, Fig. S10e). Micron-sized microlumens consistently developed below the apical tight junctions under DMSO conditions, and they contained polygranules and membrane remnants, suggesting exocytosis of intracellular vacuoles into the microlumens (Fig. 4e). By contrast, cells under N2B27 control conditions showed apically located lysosomes and Golgi, with no microlumens present (Fig. 4e).

We next used live-cell holotomography,^57^ which resolves cell membranes and the apical-basal axis in a label-free, low-phototoxicity manner, to further characterize TE lumenogenesis. Under N2B27 conditions, cells remained well adhered to one another at both the apical and basal sides, with no microlumen formation throughout the experiment. Simultaneous wide-field fluorescence imaging showed that lysosomes were concentrated perinuclearly (Fig. 4f; Supplementary information, Fig. S10f and Video S4). By contrast, DMSO-differentiated TE cells developed numerous microlumens on the basal side while maintaining tight cell–cell junctions on the apical side. Consistent with the light microscopy and TEM results, lysosomes coincided with areas of microlumen formation (Fig. 4d, e, g; Supplementary information, Fig. S10f). The microlumens grew larger, coalesced, and caused apically sealed TE cells to cavitate to form cysts (Fig. 4h; Supplementary information, Videos S5, S6). Together, the above genetic and imaging data strongly suggest the importance of the lysosomal pathway for basolateral lumenogenesis and TE cavitation.

**Fig. 5:**
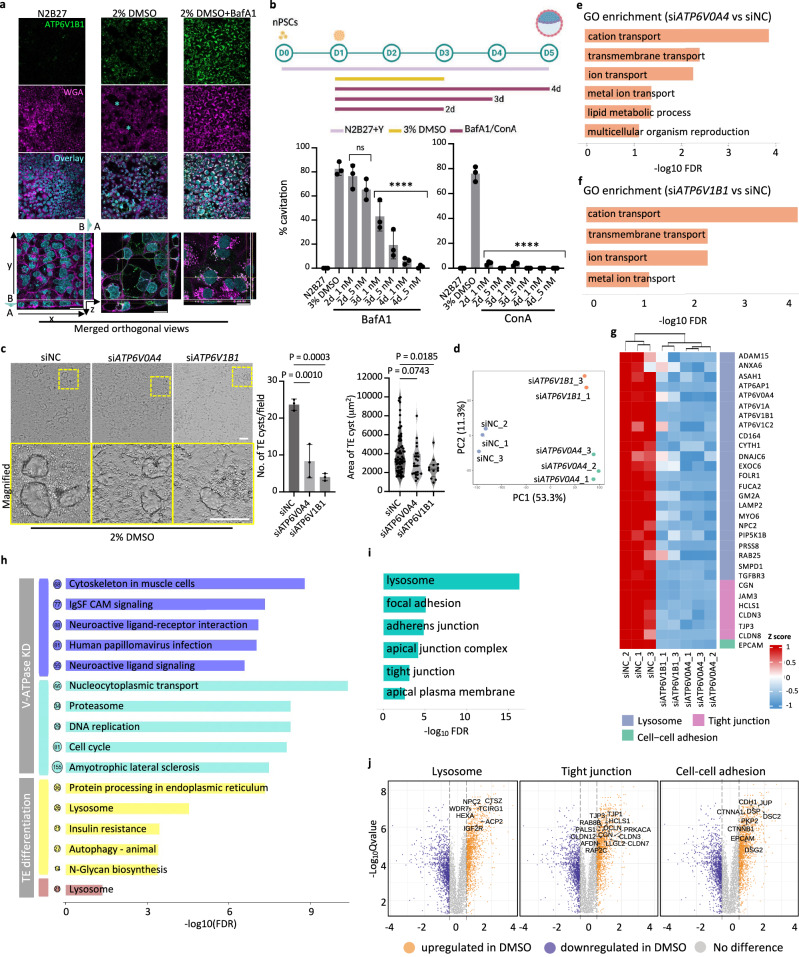
Disruption of V-ATPase function impairs TE cyst formation and cavitation in vitro. **a** Immunofluorescence analysis showing the expression of *ATP6V1B1* (green) and wheat germ agglutinin (WGA, magenta) under different treatment conditions on day 3 of differentiation (*n* = 3). Scale bars, 50 μm. The merged orthogonal views on the far right show higher-magnification images. Cyan asterisk: TE cyst. The cell drawings next to the x-z and y-z views indicate the apical (A) and basal (B) orientation of the cells. Scale bars, 20 μm. **b** Schematic showing the treatment conditions for BafA1 and ConA in the blastoid formation assay (top). Bar graphs (bottom) show the effects of BafA1 and ConA on blastoid cavitation. Data are presented as the mean ± standard deviation of three independent experiments. Statistical analysis was performed using one-way ANOVA followed by Dunnett’s post hoc test. *P* values are as indicated. **c** Representative brightfield images showing the effect of *ATP6V0A4* and *ATP6V1B1* KD on TE cyst formation (left). Scale bar, 100 µm. Bar graphs show the quantification of the number of TE cysts formed and their area (right). Data are presented as the mean ± standard deviation of three independent experiments. One-way ANOVA followed by Dunnett’s post hoc test was used. *P* values are as indicated. **d** PCA plot of bulk RNA-seq analysis of siNC, si*ATP6V0A4*, and si*ATP6V1B1*. **e,** **f** GO enrichment analysis of differentially downregulated genes between si*ATP6V0A4* and siNC (**e**) and between si*ATP6V1B1* and siNC (**f**). **g** Heatmap displaying the expression levels of lysosome-, tight junction-, and cell–cell adhesion-related genes across the indicated conditions. **h** Bar plot showing enriched KEGG pathways for the blue, turquoise, yellow, and brown WGCNA modules. Pathways are ranked by −log_10_(FDR), with module colors corresponding to co-expression modules associated with V-ATPase KD (blue and turquoise) or TE differentiation (yellow and brown). **i** GO enrichment analysis of differentially downregulated proteins showing pathways enriched in the DMSO treatment. **j** Volcano plots showing differential protein abundance between DMSO-treated and control groups for proteins associated with the lysosome (left), tight junction (middle), and cell–cell adhesion (right).

To test the requirement for functional lysosomes, we blocked lysosome acidification using a specific inhibitor of V-ATPases, bafilomycin A1 (BafA1), during TE differentiation. As expected, BafA1 dramatically reduced the signals of fluorescent indicators of low pH. Importantly, BafA1 potently and significantly blocked TE cavitation, even at a low dose, without causing gross cellular toxicity, a phenotype recapitulated using another specific V-ATPase inhibitor, concanamycin A (ConA) (Supplementary information, Fig. S11a–d). Cells treated with BafA1 still expressed ATP6V1B1 (a subunit of the V-ATPase complex) on vesicular membranes. However, they showed a paucity of large intracellular vacuoles and a dramatic accumulation of numerous small ATP6V1B1-stained lysosomes that, in turn, accumulated high levels of glycoproteins, a phenomenon not observed during normal TE differentiation (Fig. 5a; Supplementary information, Fig. S11e). These observations demonstrate that V-ATPase is essential for lysosome fusion, formation of intracellular vacuoles, and degradation and recycling of glycoproteins in lysosomes. Consistent with observations in 2D culture, BafA1 and ConA significantly reduced the cavitation rate under conditions of 3% DMSO blastoid induction compared with the control (Fig. 5b; Supplementary information, Fig. S11f, g).

Next, we investigated the functional roles of the V-ATPase subunits selectively upregulated in the TE, including *ATP6V0A4* and *ATP6V1B1*, during DMSO-induced TE cyst formation. To this end, we performed siRNA-mediated knockdown (KD) of *ATP6V0A4* and *ATP6V1B1* in nPSCs (Supplementary information, Fig. S12a, b). KD of either subunit resulted in a significant reduction in TE cyst formation (Fig. 5c). Importantly, this defect was rescued by overexpression of the corresponding *ATP6V0A4* or *ATP6V1B1* construct (Supplementary information, Fig. S12c–e), demonstrating that these V-ATPase subunits are essential for TE morphogenesis. To investigate the molecular consequences of disrupting V-ATPase during TE differentiation, we performed RNA-seq on nPSCs transfected with siRNAs targeting *ATP6V0A4* or *ATP6V1B1*, followed by a DMSO-induced TE differentiation assay. PCA and Pearson correlation analysis revealed distinct transcriptional profiles, clearly separating the *ATP6V0A4* and *ATP6V1B1* KD groups from the control (siNC) (Fig. 5d; Supplementary information, Fig. S12f). In addition, analysis of differential gene expression (DGE) confirmed unique transcriptional profiles across the KD and siNC groups (Supplementary information, Fig. S12g, h). GO enrichment analysis indicated significant downregulation of pathways associated with lysosomal function, including ion transport, transmembrane transport, and lipid metabolic processes, following *ATP6V0A4* or *ATP6V1B1* KD (Fig. 5e, f). Similarly, Kyoto Encyclopedia of Genes and Genomes (KEGG) pathway analysis identified “lysosome” as the only significantly affected pathway in both the si*ATP6V0A4* and si*ATP6V1B1* groups, consistent with the requirement for lysosomal activity in TE cyst formation (data not shown). Consistent with these findings, both si*ATP6V0A4* and si*ATP6V1B1* conditions showed pronounced downregulation of lysosomal genes relative to the siNC control (Fig. 5g). Genes associated with tight junction formation and cell–cell adhesion were also significantly affected, indicating broader disruption of epithelial organization upon V-ATPase depletion (Fig. 5g). Weighted gene co-expression network analysis (WGCNA) revealed a clear separation between transcriptional programs associated with V-ATPase-dependent perturbations and those linked to TE differentiation induced by DMSO or PDA83 (Fig. 5h). Modules correlated with KD conditions (blue and turquoise) were enriched for pathways involved in nucleocytoplasmic transport, proteasome function, and cell cycle regulation, indicating that loss of V-ATPase activity elicited broad homeostatic and stress-associated responses affecting cellular architecture and proliferative control. By contrast, modules associated with TE differentiation (yellow and brown) were selectively enriched for lysosomal pathways, protein processing in the endoplasmic reticulum, and *N*-glycan biosynthesis, highlighting lysosomal activation as an integral feature of the TE differentiation program (Fig. 5h). Notably, lysosome-related genes were specifically enriched in TE-associated modules and largely absent from KD-associated modules, indicating that V-ATPase-dependent lysosomal function is transcriptionally coordinated with TE differentiation rather than representing a secondary stress response (Supplementary information, Fig. S12i). Collectively, these data position V-ATPase-linked lysosomal pathways at the intersection of TE fate acquisition and morphogenetic competence, with their perturbation activating compensatory transcriptional programs incompatible with efficient cavitation.

To confirm our transcriptomic and functional findings using an independent analytical platform, we performed quantitative proteomic profiling of control and DMSO-treated samples during TE differentiation. Consistent with our transcriptional and functional analyses, DMSO treatment resulted in marked enrichment of lysosome-associated proteins, supporting activation of the V-ATPase lysosomal axis during TE cyst formation (Fig. 5i). In addition to lysosomal components, pathway enrichment analysis revealed significant upregulation of proteins involved in focal adhesion, adherens junctions, tight junctions, the apical junction complex, and the apical plasma membrane (Fig. 5i, j). These protein-level changes indicate that DMSO not only induces lysosomal activity but also promotes epithelial polarization and cell–cell adhesion programs essential for TE morphogenesis and lumen formation. Collectively, these findings reveal lysosomal pathway processes as previously unappreciated contributors to basolateral lumenogenesis during human blastocyst cavitation.

### V-ATPase is required for blastocyst cavitation in mice and humans

**Fig. 6:**
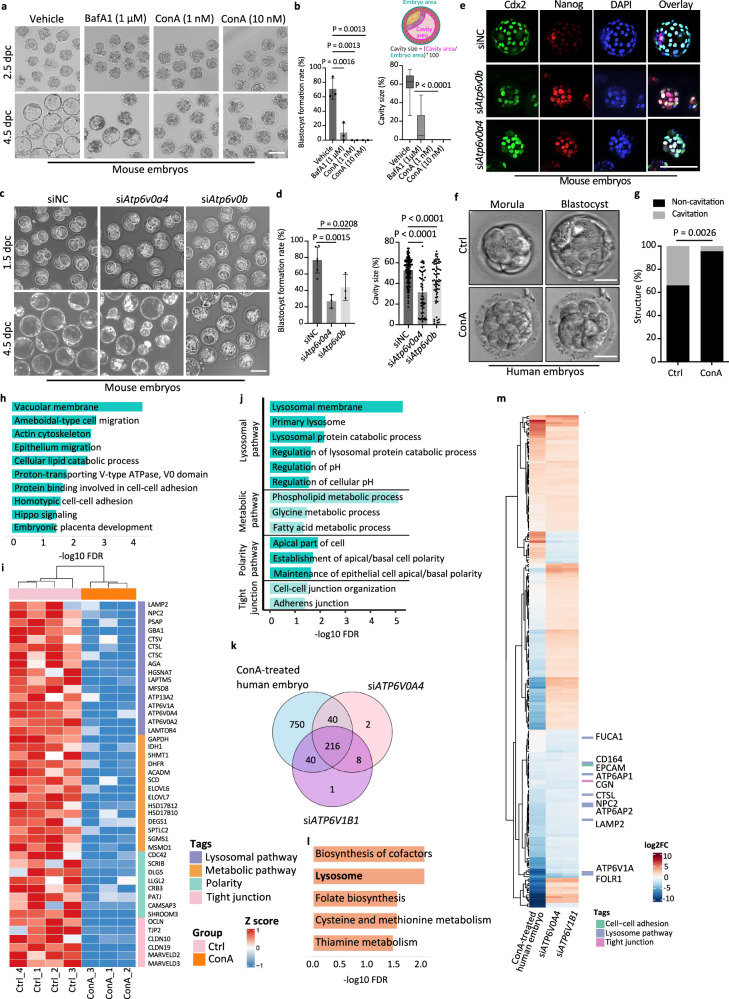
In vivo validation of V-ATPase function in mouse and human embryos. **a** Brightfield images showing the effects of BafA1 and ConA at various concentrations on mouse embryo cavitation (images cropped from Supplementary information, Fig. S13b). Scale bar, 50 μm. **b** Graphs showing the blastocyst formation rate and cavity size of mouse embryos. Data are presented as the mean ± standard deviation of three independent experiments. One-way ANOVA followed by Dunnett’s post hoc test was used. *P* values are as indicated. **c** Brightfield images showing the effect of siRNA-mediated KD of *Atp6v0a4* and *Atp6v0b* on mouse embryo cavitation (images cropped from Supplementary information, Fig. S13e). Scale bar, 25 μm. **d** Graphs showing the blastocyst formation rate and cavity size of mouse embryos. Data are presented as the mean ± standard deviation of three independent experiments. One-way ANOVA followed by Dunnett’s post hoc test was used. *P* values are as indicated. **e** Immunofluorescence analysis showing the expression of Cdx2 and Nanog under the indicated conditions (*n* = 3). Scale bar, 30 μm. **f** Brightfield images showing the effect of ConA (1 nM) on human embryo cavitation. Scale bars, 25 μm. **g** Graph showing the cavitation rate in control (Ctrl, *n* = 56) and ConA-treated human embryos (*n* = 45). A paired Student’s *t*-test was used, and the *P* value is as indicated. **h** GO enrichment analysis of differentially downregulated genes in human embryos under ConA treatment. **i** Heatmap showing the expression of different pathway-related genes in Ctrl and ConA-treated human embryos. **j** GO enrichment analysis of differentially downregulated genes in human embryos under ConA treatment. **k** Venn diagram showing overlapping DEGs among ConA-treated human embryos, si*ATP6V0A4*, and si*ATP6V1B1* groups. **l** KEGG analysis of the shared DEGs from **k**. **m** Heatmap showing the expression patterns of the overlapping genes from **k**.

To assess the in vivo significance of our findings, we performed pharmacological and genetic interventions targeting V-ATPase function during mouse blastocyst cavitation. First, morula-stage mouse embryos were treated with the V-ATPase inhibitor BafA1 (*n* = 46, three independent replicates) or ConA (*n* = 40, three independent replicates) (Supplementary information, Fig. S13a). These treatments resulted in impaired blastocyst cavitation, characterized by a reduced formation rate and smaller blastocoel cavity size compared with controls (*n* = 49, three independent replicates) (Fig. 6a, b; Supplementary information, Fig. S13b). To further confirm the requirement for V-ATPase activity during cavitation, we performed siRNA-mediated KD of V-ATPase genes in mouse embryos. As a first step, we examined the expression levels of V-ATPase subunits during early mouse development using published data.^58^ We found that *Atp6v0a4* and *Atp6v0b* were highly upregulated during the morula stage, whereas *Atp6v1b1* showed minimal expression (Supplementary information, Fig. S13c). Based on these findings, we selected *Atp6v0a4* and *Atp6v0b* for KD in mouse embryos. As expected, embryos injected with siRNA targeting *Atp6v0a4* (*n* = 66, three independent replicates) or *Atp6v0b* (*n* = 72, three independent replicates) exhibited a markedly reduced blastocyst formation rate and a significantly smaller blastocoel cavity compared with embryos injected with non-targeting control siRNA (siNC; *n* = 70, three independent replicates) (Fig. 6c, d; Supplementary information, Fig. S13d, e). Immunofluorescence analysis revealed impaired cavitation in the KD groups, while lineage markers such as Cdx2 and Nanog remained expressed, suggesting minimal cytotoxicity (Fig. 6e). Collectively, these data robustly support our in vitro observations and underscore the conserved and essential role of V-ATPase in early mammalian embryo cavitation.

Given these findings in mouse embryos, we next assessed the functional relevance of V-ATPases in human embryo cavitation. To do so, we treated in vitro-cultured morula-stage human embryos (see Methods) with a low concentration of ConA (*n* = 45, five independent replicates), which significantly reduced the cavitation rate relative to controls (*n* = 56, seven independent replicates) (Fig. 6f, g). ConA treatment did not increase cell death (Supplementary information, Fig. S13f). This observation is consistent with findings from mouse embryo studies, as well as in vitro 2D TE and 3D blastoid formation assays (Figs. 5a, b and 6a, b; Supplementary information, Figs. S11a–d, f, g and S13b). It is important to note that V-ATPase inhibition affected the lysosomal pathway in vitro (Fig. 5). To determine whether similar molecular changes occur in human embryos, we performed Smart-seq2 analysis on individual ConA-treated (*n* = 3) and control (*n* = 4) embryos. PCA and correlation analyses demonstrated distinct transcriptional profiles that clearly separated ConA-treated embryos from controls (Supplementary information, Fig. S13g, h). DGE analysis identified a broad set of DEGs between the ConA-treated and control groups (Supplementary information, Fig. S12i). As expected, these DEGs are enriched in GO terms related to V-ATPase and lysosomal function (e.g., vacuolar membrane, cellular lipid catabolic process, and V-type ATPase V0 domain). They are also enriched in GO terms associated with basolateral lumenogenesis and epithelial cavitation (e.g., actin cytoskeleton, epithelial migration, and cell–cell adhesion), as well as developmental pathways (e.g., embryonic placenta development and Hippo signaling), suggesting disruption of key developmental processes (Fig. 6h). Lysosome-related genes, including several V-ATPase subunits, were markedly downregulated in ConA-treated human embryos (Fig. 6i). GO analysis of the downregulated gene set revealed enrichment in the lysosomal pathway, autophagy, and metabolic processes (Fig. 6j). Together, the observed morphogenetic and transcriptomic defects demonstrate that V-ATPase function is essential for successful cavitation in human embryos by maintaining proper lysosomal function.

To assess the fidelity and robustness of our DMSO model, we next performed a comparative transcriptomic analysis between our in vitro model and in vivo human embryo datasets. Specifically, we compared the DEGs identified in ConA-treated human embryos with those from si*ATP6V0A4*- and si*ATP6V1B1*-treated in vitro samples. We observed significant gene overlap among the three datasets, with 216 genes shared by all groups (Fig. 6k). GO analysis of these overlapping genes revealed significant enrichment of lysosomal pathway genes, together with those involved in metabolic processes (Fig. 6l, m). Direct comparison of transcriptomic alterations between si*ATP6V0A4*- and si*ATP6V1B1*-treated in vivo and in vitro samples would have been ideal; however, obtaining a sufficient number of donated human embryos to conduct parallel microinjection experiments with treatment and control groups was impractical. Nevertheless, these concordant findings underscore how the DMSO model facilitated the discovery of a previously unrecognized mechanism underlying basolateral lumenogenesis, providing important mechanistic insight into the cellular remodeling events essential for TE cavitation. The strong concordance between in vitro and in vivo observations across both mouse and human embryos confirms the physiological relevance of the DMSO model and underscores its utility for recapitulating key developmental processes. Furthermore, our study emphasizes the essential role of V-ATPases in coordinating lysosomal function during early embryogenesis, providing a valuable framework for future investigations into the regulation of blastocyst formation and implantation.

### DMSO modulates membrane biophysical properties during cavitation initiation

Membrane biophysical properties are increasingly recognized as critical regulators of early embryonic morphogenesis, including compaction, polarization, and lumen formation.^59–61^ To examine whether DMSO modulates these properties during blastoid formation, we assessed membrane biophysical properties in our system.

**Fig. 7:**
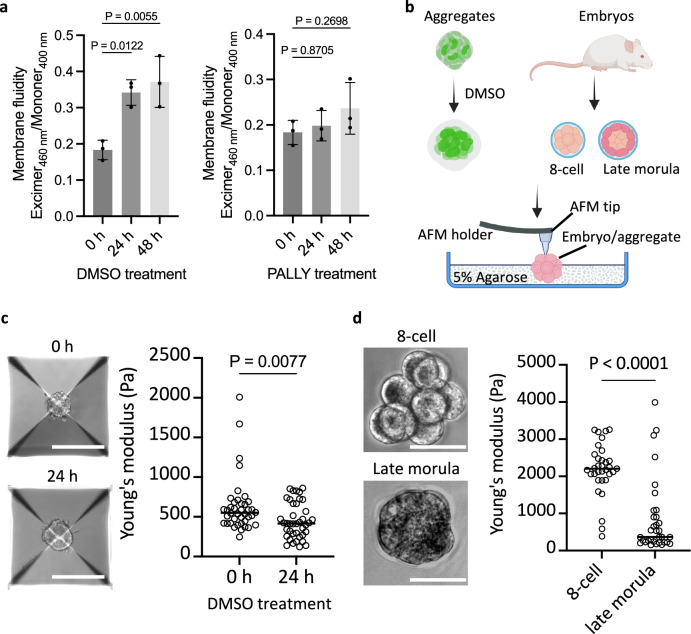
DMSO modulates membrane biophysical properties characteristic of early embryonic development. **a** Quantification of membrane fluidity measured by the excimer-to-monomer fluorescence ratio (460 nm/400 nm) in aggregates following DMSO (left) or PALLY (right) treatment at the indicated time points. Data are presented as the mean ± standard deviation of three independent experiments. One-way ANOVA followed by Dunnett’s post hoc test was used. *P* values are as indicated. **b** Schematic illustration of the experimental setup for AFM measurements. **c** Representative brightfield images showing aggregates used for AFM measurements (left) and quantification of Young’s modulus (right) of compacted aggregates before (0 h) and after (24 h) DMSO treatment (*n* = 40). Scale bars, 200 μm. Student’s *t*-test was used, and the *P* value is as indicated. **d** Representative brightfield images showing mouse embryos used for AFM measurements (left) and quantification of Young’s modulus of mouse embryos at the 8-cell and late morula stages (right) (*n* = 33). Scale bars, 50 μm. Student’s *t*-test was used, and the *P* value is as indicated.

We first measured membrane fluidity using a fluorescence-based assay following DMSO exposure. DMSO treatment induced a significant, time-dependent increase in membrane fluidity compared with untreated controls (Fig. 7a, left). Notably, the temporal dynamics of this increase coincided with the onset of lumen formation in the blastoid system. By contrast, parallel measurements under PALLY conditions did not show significant changes in membrane fluidity over the same time window (Fig. 7a, right), indicating that enhanced membrane fluidity is not a general consequence of blastoid induction but rather a distinct effect associated with DMSO treatment.

Because membrane fluidity is closely linked to mechanical properties such as cortical tension and contractility,^15,59^ we next assessed membrane stiffness using atomic force microscopy (AFM). AFM measurements were performed on compacted aggregates immediately before and after DMSO exposure, corresponding to the developmental window preceding cavitation (Fig. 7b). DMSO-treated aggregates exhibited a significant reduction in membrane stiffness relative to untreated controls (Fig. 7c), indicating pronounced membrane softening following DMSO treatment. To assess the in vivo relevance of these mechanical changes, we performed AFM measurements on mouse embryos at defined developmental stages. Late morula embryos (post-compaction, pre-cavitation) exhibited significantly lower membrane stiffness compared with early-stage 8-cell embryos (Fig. 7d), mirroring findings in the DMSO system.

Collectively, these data demonstrate that DMSO induces coordinated changes in membrane biophysical properties in vitro that closely parallel the biomechanical transitions that occur during early embryonic development in vivo. Importantly, these biophysical changes are not recapitulated under defined pathway-based induction conditions, supporting a distinct membrane- and mechanics-associated component of DMSO-mediated blastoid morphogenesis.

## Discussion

Human blastoids offer a valuable model for studying TE specification and morphogenesis, key areas of research on human blastocysts, implantation, and placental development. PD,^5–11,62^ A83,^5–11,62^ and many other small molecules and growth factors have been commonly used to drive nPSCs toward the TE fate and subsequent blastoid formation.^4,5,10,11^ The use of various factors targeting multiple pathways has been shown to result in different pathway activities among blastoid protocols and between blastoids and blastocysts,^12^ likely due to pleiotropy and complex crosstalk. This presents significant challenges for understanding the mechanism of TE differentiation. Further complicating the matter is the fact that existing blastoid protocols require cell aggregates, which may respond heterogeneously to the applied factors. Here, we show that a single small molecule, DMSO, potently induces TE from diverse types of human nPSCs. TE induction by DMSO can be leveraged to enable clonal blastoid generation from single nPSCs without additional pharmacological or genetic perturbations.

DMSO leads to changes in morphology, gene expression, and chromatin accessibility consistent with those observed during TE differentiation in vitro and in human blastocysts. Although the induction of TE fate by DMSO appears less potent than that by PDA83 in bulk RNA-seq and ATAC-seq analyses (Supplementary information, Figs. S1h and S2a), we interpret this as being consistent with the observation that window-specific treatment with DMSO alone can induce blastoids, whereas PDA83 culture cannot.^6^ This suggests that DMSO induces a TE fate in responsive cells (likely the outer cells in compacted aggregates) while preserving the EPI and PE lineage potential of the remaining cells. This behavior resembles the regulative capacity of the embryo, well-documented in classic experiments, in which cells in early blastocysts remain plastic and ICM cells can re-adopt a TE fate when exposed to the outside environment.^55,63,64^ By contrast, supra-physiological levels of signaling manipulation may override such in vivo plasticity and limit faithful recapitulation of blastocyst development.

Blastoids induced by DMSO resemble human blastocysts in morphology, lineage composition, and the ability to differentiate into peri-implantation lineages in attachment assays. Notably, attached DMSO blastoids developed a well-formed amniotic cavity-like central lumen within the EPI (Fig. 3d–f; Supplementary information, Fig. S7a, b), which was not observed under PALLY conditions. EVT differentiation remains a challenge in current blastoid models. Karvas et al.^9^ reported that expression of HLA-G, a definitive marker of EVT differentiation, was not detected until day 18 in their blastoid model, indicating delayed timing compared with EVT differentiation during natural human implantation.^5,34,45^ Similarly, another study acknowledged delayed EVT differentiation as a limitation of their model.^65^ By contrast, in attached DMSO blastoids, EVT differentiation, as evidenced by HLA-G expression, occurred in alignment with the expected timeline of human embryonic development.^5,34,45^ In addition, the above-mentioned blastoid models^9,65^ required a thicker 3D extracellular matrix (ECM) for post-implantation development. By contrast, the DMSO-only blastoid model successfully advanced through post-implantation stages without the need for 3D ECM culture, highlighting its versatility and robustness, likely due to the simpler signaling environment. In summary, we used the extraembryonic potential of nPSCs to create an integrated model system for early human embryogenesis encompassing both pre- and post-implantation stages. These results suggest that DMSO blastoids can better model certain developmental processes, complementing existing models.

The accumulation of intracellular vacuoles and basally localized lysosomes was reproducibly observed during DMSO-induced TE cavitation. These features have been described in the mouse embryo,^55,56,66^ but their significance remains unclear. The DMSO system offers a robust model for studying these phenomena in humans. Our observations suggest that lysosomes are involved in reorganization of the basolateral membrane and in the formation of microlumens and that lysosomal acidification by V-ATPases is required for human TE cavitation. V-ATPase genes such as *ATP6V1B1* and *ATP6V0A4* are among the genes upregulated in the TE lineage of pre-implantation human embryos in a published scRNA-seq dataset.^35^ More recently, advanced scRNA-seq analyses using Entropy Sort Feature Weighting identified *ATP6V1B1* and *ATP6V0A4* as two of four markers of the ICM/TE-to-TE branch point.^67^ We observed specific upregulation of *ATP6V1B1* and *ATP6V0A4* in DMSO-differentiated samples. Importantly, loss-of-function and rescue experiments targeting ATP6V1B1 and ATP6V0A4 demonstrated that these V-ATPase subunits are required for TE cavitation, consistent with our findings in both mouse and human embryos.

Mutations in the human *ATP6V1B1* and *ATP6V0A4* genes have been associated with several medical conditions, most notably autosomal recessive distal renal tubular acidosis, frequently accompanied by sensorineural hearing loss. These mutations lead to defective proton secretion in the kidney and inner ear, disrupting acid–base balance and causing metabolic acidosis, nephrocalcinosis, and auditory impairments that typically manifest in early childhood.^68,69^ These findings underscore the crucial roles of *ATP6V1B1* and *ATP6V0A4* in maintaining homeostasis in the renal and auditory systems.^70,71^ In mouse models, further evidence supports an essential role for V-ATPase in early development. Sun-Wada et al.^72^ demonstrated that in cultured mouse pre-implantation embryos, acridine orange staining revealed diffuse red fluorescence indicative of acidic compartments during early stages. After compaction, this fluorescence became concentrated in cortical and perinuclear regions and exhibited distinct patterns between ICM and TE cells in blastocysts. Importantly, these acidic compartments disappeared upon treatment with BafA1, confirming the role of V-ATPase in establishing intracellular acidity. In addition, studies of *Atp6v0c* mutant embryos, which lack a c-subunit of V-ATPase, revealed that apical-basal polarity of the visceral endoderm epithelium was not properly maintained, resulting in abnormal tissue morphology.^73^ These findings suggest that V-ATPase is crucial not only for intracellular acidification but also for establishing and maintaining cellular polarity during early embryogenesis. In *Xenopus* embryos, V-ATPase was shown to play a pivotal role in establishing left-right (LR) asymmetry.^74^ In that study, pharmacological inhibition of V-ATPase using BafA1 and ConA led to a pronounced incidence of heterotaxia, indicating that V-ATPase activity is critical for proper LR patterning and asymmetric development in *Xenopus*. However, due to significant differences in developmental timelines and cavitation mechanisms between *Xenopus* and mammals, particularly humans, extrapolating these findings to mammalian embryogenesis has been challenging.

The human embryos used in the validation experiments were tripronuclear (3PN). Although they arise from aberrant fertilization, 3PN embryos have been reported to retain substantial developmental potential, with evidence suggesting that they may have the ability to self-correct into euploid blastocysts.^75^ Reported blastocyst formation rates for 3PN embryos range from 10.75% to 62.5%, with variation reflecting differences in oocyte origin and culture conditions.^75–77^ Although 3PN embryos are typically discarded in routine clinical practice when surplus diploid embryos are available, they become clinically valuable in patients with absent or few 2PN embryos. Furthermore, a recent case report documented a healthy live birth following the transfer of a euploid blastocyst derived from a 3PN embryo identified by preimplantation genetic testing, providing compelling evidence of the developmental competence of such embryos and supporting their use as a biological model for studying early human developmental mechanisms.^78^

Despite these insights, our understanding of the roles of V-ATPase and the mechanisms of intra- and extracellular acidification during early human embryogenesis remains limited. In this study, we demonstrated that V-ATPase-mediated lysosomal acidification is required for blastoid cavitation, a critical morphogenetic step in early mammalian development. Experiments in both mouse and human embryos strongly confirmed the essential role of V-ATPase activity in proper blastocoel formation (Fig. 6), supporting a conserved requirement for V-ATPase-dependent acidification during blastocyst cavitation across mammalian species. Our findings indicate that acidic intracellular compartments generated by V-ATPase are essential for early human development by facilitating key morphogenetic events such as cavitation. These results add to a growing body of evidence underscoring the importance of V-ATPase in early development and highlight the need for further research to elucidate the precise molecular mechanisms through which V-ATPase-mediated acidification influences human embryogenesis. Our transcriptomic perturbation and proteomic analyses provide further support that DMSO activates TE regulatory networks consistent with those induced by conventional TE differentiation cues, rather than eliciting nonspecific stress responses. Together, these data represent an initial step toward molecular dissection of V-ATPase regulation and mechanism and suggest that the DMSO-based cavitation model provides a valuable platform for modeling in vivo-relevant morphogenetic events.

DMSO, a staple cryoprotectant used in the clinical preservation of human embryos, plays a crucial role in assisted reproductive technologies.^79–82^ DMSO is believed to increase cell membrane permeability^25^ and facilitate ion transport through the transient formation of water pores.^83^ The membrane effects of DMSO have been proposed to underlie its differentiation effects in cells.^21^ More intriguingly, cell membrane fluctuations^15^ and hydraulic fracturing of cell–cell contacts^15^ have been shown to regulate the earliest lineage specification events in the embryo. After embryo compaction, the outer blastomeres, with exposed plasma membranes and asymmetrical cell contacts, assume the TE fate. Consistent with this framework, we observed that DMSO was effective only when applied after aggregate compaction. To investigate this phenomenon further, we treated compacted aggregates with DMSO and examined membrane biophysical properties. Our results showed that DMSO induces changes in membrane biophysical properties in a manner that parallels early embryonic development. Specifically, DMSO increases membrane fluidity and reduces membrane stiffness in compacted aggregates at the stage preceding cavitation — effects that were not observed under defined pathway-based induction conditions such as PALLY. These findings indicate that membrane biophysical modulation is a distinct feature of DMSO-mediated morphogenesis rather than a general consequence of blastoid induction. Importantly, these in vitro observations mirror biomechanical transitions observed in vivo. Mouse embryos exhibit progressive reductions in surface tension from the zygote through the 8-cell stage and into later developmental stages,^84^ consistent with decreasing cortical tension and cell contractility during blastocyst morphogenesis.^15,59^ Such mechanical softening facilitates lumen expansion and hydraulic fracturing of cell–cell contacts, suggesting that DMSO induces an embryo-like biomechanical state that lowers the energetic barrier for cavity formation. Nevertheless, these membrane-related observations represent an initial step toward understanding how biophysical regulation contributes to early human development. We anticipate that this work will stimulate future studies using human blastoid models to directly interrogate membrane mechanics and force-mediated morphogenetic processes.

In this study, we used a DMSO-based model that promotes TE differentiation without the need for additional pharmacological or genetic manipulation. This simplified system enables the formation of blastoid-like structures and provides a tractable in vitro platform to investigate the molecular mechanisms of TE cavitation and blastocoel formation. Through this model, we identified the lysosomal pathway as a key regulator of basolateral lumenogenesis and demonstrated that intracellular vesicular acidification by V-ATPase is required for blastocoel development in mice and humans. These findings highlight the utility of the DMSO model for dissecting pathways critical to early developmental events and provide new insight into conserved cellular processes in early human and mouse embryogenesis.

## Materials and methods

### Ethics statement

All research procedures involving human PSCs and blastoid models were conducted at King Abdullah University of Science and Technology (KAUST) following review and approval (approval# 21IBEC055) by the KAUST Institutional Biosafety and Bioethics Committee (IBEC). KAUST IBEC, as the local ethics committee registered with the National Committee of Bioethics (NCBE) of Saudi Arabia, fulfils the role of an ESCRO (Embryonic Stem Cell Research Oversight) committee. These studies complied with the International Society for Stem Cell Research (ISSCR) 2021 Guidelines for Stem Cell Research and Clinical Translation. The PSCs used in this research comprised chemically reset human induced pluripotent stem cells (hiPSCs) and human embryonic stem cells, both previously reported in integrated human embryo models. Specifically, the human embryonic stem cell lines RUES2 and HNES1 were used in this study; both are established lines approved for research use and were obtained from recognized sources. No new human embryonic stem cell lines were derived. Stem cell-derived embryo models (blastoids) recapitulated embryonic developmental features in vitro and were cultured for no more than 10 days, consistent with the developmental stage stipulated by the 14-day rule for human embryo cultures. The nomenclature used for the embryo models aligns with community and ISSCR recommendations.

Human embryo studies were approved by the IRB of the Third Affiliated Hospital of Guangzhou Medical University (approval# 20230001). The embryos used were unsuitable for clinical applications and were collected after obtaining written informed consent from women aged 25–35 years with no familial genetic disorders or infectious diseases. All human embryo experiments strictly adhered to the 2016 Declaration of Helsinki guidelines for medical research involving human subjects and complied with the ethical principles for human-assisted reproductive technologies issued by the Ministry of Health of China in 2003, relevant Chinese laws and policies, and the 2021 ISSCR guidelines.

Research involving mouse embryos was conducted under protocols approved by the Animal Research Committee of Zhejiang University (approval# ZJU20230458) in strict accordance with established guidelines for ethical animal use. Animal experiments were performed at KAUST in accordance with institutional guidelines and approved by the IACUC (approval# 24IACUC016).

### Cell culture

The human PSCs included in this study are chemically reset (cR) SC9N hiPSCs. The RUES2 human ESC line was a generous gift from Dr. Ali H. Brivanlou and was reset in-house. These cells underwent epigenetic resetting as per a previously established protocol.^85^ In summary, primed-state PSCs were transitioned into iMEFs and treated with PD0325901 (hereafter PD) (1 µM), LIF (10 ng/mL), and VPA (1 mM) for 3 days. Subsequently, cells were transferred to a medium containing 1 µM PD, 2 µM XAV-939, 2 µM Gö6983, and 10 ng/mL LIF (hereafter PXGL). By day 10, naïve dome-shaped colonies emerged and were purified by fluorescence-activated cell sorting (FACS) for SUSD2 or via gelatinization for several passages. To generate HENSM naïve PSCs, PXGL-maintained naïve PSCs were propagated under HENSM conditions for at least three passages prior to downstream experimental use. The HENSM cell culture medium was formulated according to an established protocol,^86^ incorporating PXGL medium supplemented with 1:500 Geltrex, 50 µg/mL vitamin C, 1.2 µM ROCK inhibitor, 1 µM dasatinib (SRCi), and 5 ng/mL human activin A. Cell cultures were maintained at 37 °C under 7% CO_2_ and 5% O_2_. We used a previously established protocol^87^ to generate 8CLCs. In brief, nPSCs were dissociated into single cells and seeded at a density of 3 × 10^4^ cells per well on MEF feeder cells in a 6-well plate. Cells were cultured in PXGL medium for 24 h. On the following day, the PXGL medium was substituted with an enhanced 4CL medium (e4CL) comprising N2B27 basal medium supplemented with 10 nM UNC1999 (Cayman Chemical, Cat# 14621), 5 nM TSA (Cell Signaling Technology, Cat# 9950), 1 µM PD (VWR, Cat# 101761-458), 5 µM IWR-1 (Sigma, Cat# I0161-25MG), 20 ng/mL human LIF (Alliance Global, Cat# 8911SC), 20 ng/mL activin A (Sigma, Cat# A4940-10UG), 50 µg/mL L-ascorbic acid (Sigma, Cat# 33034), and 0.2% (v/v) Matrigel (Corning, Cat# 354277). Cells were maintained under hypoxic conditions, and the medium was refreshed daily until day 5. The HNES1 naïve embryonic PSC line was a generous gift from Drs. Austin Smith and Ge Guo and was cultured as described previously.^8^

### Mouse embryos

C57BL/6J mice were housed in the animal facility of Zhejiang University under a 12-h light-dark cycle. Their diet was procured from Xietong (Cat# 1010085). Female C57BL/6J mice (4–6 weeks old), male C57BL/6J mice (8 weeks old), and male DBA mice (8 weeks old) were obtained from SLAC Animal. Mice had ad libitum access to food and water and were maintained at 22–26 °C with 50%–70% humidity.

To collect preimplantation embryos, 4-week-old C57BL/6J female mice received an intraperitoneal injection of 7.5 IU pregnant mare serum gonadotropin (San-Sheng Pharmaceutical), followed 48 h later by an injection of 7.5 IU hCG from the same supplier. The superovulated females were mated overnight with adult C57BL/6J males after hCG administration. Zygotes were collected from the ampullae of the oviducts 24 h post hCG injection and treated with 100 µg/mL hyaluronidase in M2 medium (Sigma-Aldrich) to remove cumulus cells.

CD1 mice were housed in the KAUST animal facility under standard conditions with a 12-h light-dark cycle. Superovulation was induced in 3-week-old CD1 female mice by intraperitoneal injection of 5 IU pregnant mare serum gonadotropin, followed 48 h later by 5 IU hCG. Females were then mated overnight with adult CD1 males. Preimplantation embryos were collected at defined developmental stages, including 8-cell embryos at embryonic day E2.5 and late morulae at E3.0, by flushing the uterus with M2 medium. Collected embryos were incubated in 0.5% Pronase (Sigma Aldrich; Cat# 53702-50KU) at 37 °C for 5 min to remove the zona pellucida, washed once in M2 medium, and transferred using a mouth pipette for subsequent fixation and AFM analysis.

### Human embryos

Triploid human embryos, derived from zygotes containing three pronuclei (3PN) and deemed unsuitable for transfer, were donated by female volunteers aged 25–35 years in accordance with the ethical guidelines of the Third Affiliated Hospital of Guangzhou Medical University. Donors had no known family history of genetic disorders or infectious diseases. 3PN zygotes were identified during the in vitro fertilization procedure on the basis of the clear presence of three pronuclei after fertilization. These zygotes were subsequently cultured to the morula stage using time-lapse monitoring (Vitrolife) with G-1 (G1 PLUS, Vitrolife Sweden AB) and G-2 (G2 PLUS, Vitrolife Sweden AB) culture media. Only morulae that exhibited high-quality morphology without signs of developmental delay or arrest were selected for inhibitor treatment experiments.

### 2D TE differentiation assay

To prepare PXGL nPSCs for TE and blastoid differentiation, nPSCs were grown on iMEFs in PXGL medium for 3–4 days. Next, cells were dissociated using TrypLE for 5 min at 37 °C with gentle pipetting until a single-cell suspension was achieved. TrypLE was then inactivated using 0.2% BSA in N2B27 medium, and the cells were pelleted by centrifugation. To remove any remaining iMEFs, cells were seeded on a 0.1% gelatin-coated surface for 60 min. Cells were then passed through a 40-µm strainer to eliminate clumps, centrifuged once more, and resuspended in an appropriate medium. For TE differentiation, a previously established protocol was followed.^3^ In brief, cells were resuspended in PXGL medium containing ROCK inhibitor and plated onto laminin-coated dishes. After 24 h, the medium was replaced with N2B27 supplemented with PD (1 μM) and A83 (1 μM) (or other test compounds such as RA at various concentrations) for 96 h, after which the cells were harvested for downstream analysis. Cells remained in this medium for 3–4 days for SC9N and 4–5 days for HNES1, with daily medium exchanges. DMSO was used as a vehicle for PD, A83 inhibitors, and RA. The effective DMSO concentrations in the TE experiments were 0.004%, 0.004%, and 0.01% for PD03, A83, and RA, respectively.

For STB and EVT differentiation, experiments were performed as described previously,^32^ with modifications. In brief, cells that had been subjected to DMSO or PDA83 treatment for 4 days during the TE differentiation experiment were transferred to STB or EVT culture medium. For STB differentiation, cells were cultured in STB medium for 3 days, the medium was then replaced, and the cells were cultured for another 3 days before analysis. For EVT differentiation, cells were cultured in EVT medium for 3 days. The medium was then replaced with EVT differentiation medium without hNRG1 and with 0.5% Matrigel for another 3 days. Subsequently, the medium was replaced with EVT medium without hNRG1 and KSR and with 0.5% Matrigel for an additional 2 days before analysis.

### Blastoid formation assay

For the formation of PALLY and PALLY + 0.5% DMSO blastoids, we adhered to a well-established protocol.^8^ Cells were initially suspended in N2B27 basal medium supplemented with ROCK inhibitor (Y) and seeded at a density of ~75 cells per microwell in 400-µm AggreWell plates (STEMCELL Technologies, Cat# 34425). Upon compaction of the cells, the medium was changed to N2B27 supplemented with PALLY components (1 µM PD, 1 µM A83, 5 µM LPA, 10 ng/mL LIF, and 10 µM Y) and cultured for 2 days. The medium was then replaced with N2B27 supplemented with LPA (5 µM) and Y (10 µM) (LY) for an additional 2 days to enable maturation of the structures. DMSO served as a vehicle for PD and A83 inhibitors. In the case of blastoids cultivated in PALLY, the overall effective DMSO concentration during the PALLY phase was 0.008%, significantly lower than concentrations known to affect blastoid formation. For the generation of DMSO-only blastoids, cells were seeded as described earlier. Upon cell compaction, fresh N2B27 basal medium was supplemented with DMSO (2% or 3%) and 10 µM Y and cultured for 2 days. The medium was switched to N2B27 containing Y (10 µM) and cultured for an additional 2 days. For all blastoid formation assays, the media were changed daily and maintained at 37 °C under 5% CO_2_ and 5% O_2_. Blastoids were characterized morphometrically as single-layer cavitated structures with diameters ranging from 150 µm to 250 µm, featuring a single inner cell mass; non-cavitated or multi-cavitated structures were excluded from subsequent analyses.

### Derivation of clonal blastoids from single nPSCs

To generate single-cell clonal blastoids from SC9N nPSCs, we followed a standard protocol using the AggreWell 400 plate. The nPSCs were cultured in PXGL medium with daily medium replacement and passaged every 3 days to maintain their healthy dome-shaped morphology. Prior to seeding, we added the ROCK inhibitor to the PXGL medium and introduced it to the cells 1 day in advance. On the day of seeding (referred to as day 0), we harvested nPSCs using TrypLE and DNase I. We confirmed the successful digestion of the cells into single cells by microscopy examination. It is worth noting that the DNase I concentration and incubation time for digestion can be adjusted as needed to achieve single-cell status. Subsequently, we seeded the nPSCs at a density of 700–900 cells per well in N2B27 medium containing the ROCK inhibitor Y. The exact seeding density may vary among cell types, but we visually confirmed single-cell seeding under the microscope. Single cells can additionally be enriched by FACS of the pooled population. Next, we treated the nPSCs with N2B27 medium supplemented with Y every other day until compacted aggregate formation was observed, typically within 6–8 days. Once the compacted aggregates had formed, we exposed the structures to the following treatment conditions: PALLY alone, PALLY with 0.5% DMSO, and 3% DMSO for a duration of 48 h. Subsequently, for the former two conditions, the structures were treated with LPA and Y, whereas the latter condition was treated with Y alone for an additional 48 h. After completing the final treatment phase, we imaged the structures and harvested them for downstream analyses.

### Immunofluorescence

All samples, except for blastoids, were grown in ibidi 8-well chambers (Cat# 80826). The samples were washed with PBS and fixed with 4% PFA for 15 min. Afterwards, the samples were washed three times with PBS and permeabilized and blocked using a solution containing 0.2% Triton X-100 and 6% normal donkey serum for 1 h. The same solution was used to dilute the primary antibody according to the manufacturer’s recommendations and to incubate the samples overnight. Following incubation, the samples were washed three times with a solution containing 0.2% Triton X-100 and 6% normal donkey serum. The secondary antibodies were diluted at a 1:500 ratio and incubated with the samples for 1 h at 37 °C. Finally, the samples were washed three more times with PBS. Antibody details are provided in Supplementary information, Table S1. Samples other than blastoids were mounted using ProLong Antifade solution with DAPI, whereas blastoids were maintained in PBS with DAPI. Images were acquired using Leica SP8, Stellaris 8, or Zeiss Celldiscoverer 7 (CD7) confocal microscopes with LSM 900 and processed using Fiji software.

For mouse embryo immunofluorescence, samples were fixed in 4% PFA for 20 min at room temperature, washed three times in PBS with 3 mg/mL polyvinylpyrrolidone (PVP–PBS, Sigma, PVP-360), permeabilized in PBS with 0.25% Triton X-100 for 1 h at room temperature, and incubated in blocking solution (0.2% BSA, 0.25% Triton X-100, 0.01% Tween 20, 2% donkey serum, and 3 mg/mL PVP in PBS) for 1 h. The embryos were then incubated at 4 °C overnight with primary antibodies. The next day, embryos were washed three times in blocking solution, incubated for 1 h at room temperature with secondary antibodies diluted 1:400 in blocking solution, and washed three times with blocking solution. Nuclei were then stained with DAPI for 10 min, and the samples were washed three times before mounting in PVP–PBS covered with mineral oil (Sigma, M8410) in a glass-bottom cell culture dish. Imaging was performed using an Olympus FV3000 fluorescence microscope at 60× magnification with an oil-immersion objective.

### Flow cytometry

Samples were dissociated by incubation in TrypLE for 5–15 min with agitation and passed through a 40-mm strainer. Samples were then stained with FACS-grade antibodies in FACS buffer for 30 min. For cell cycle analysis, samples were fixed in pre-chilled 80% ethanol at −20 °C overnight, then washed with PBS and incubated in 200 µg/mL RNase A at 37 °C for 30 min. Each sample was stained with 10 μL propidium iodide staining solution (Tonbo Biosciences, Cat# 13-6990-T200) for 20 min. All samples were analyzed and/or sorted using a BD FACSAria Fusion cytometer. Data were processed using BD FlowJo software. The Watson model in FlowJo was used for cell cycle analysis.

### Live-cell imaging

#### Live-cell imaging in 2D and 3D culture using the Zeiss CD7 microscope

Live-cell imaging was performed using a Zeiss CD7 microscope equipped with environmental control for temperature (37 °C), oxygen (5% O_2_), and carbon dioxide (7% CO_2_) to maintain physiological conditions. For blastoid formation imaging, images were acquired every 2 h using a 5× objective and a 0.5× tube lens with the oblique brightfield method. Similarly, 2D TE differentiation was monitored by seeding cells in 8-well ibidi chambers and imaging according to the same protocol used for the TE differentiation experiments. V-ATPase inhibitors, including BafA1 (10 nM) and ConA (10 nM), were added on day 2. Endocytosis events were tracked by adding pHrodo Red Dextran (Thermo Fisher Scientific, 10 μg/mL) at day 2 of differentiation and performing imaging every hour using a 20×/0.95 objective, 1× tube lens, and oblique brightfield contrast, combined with confocal imaging of the AF568 channel for pHrodo Red Dextran. Lysosome dynamics were monitored by adding LysoTracker Red DND-99 (Thermo Fisher Scientific, 50 nM) at day 2 or later, with images captured every 2 min using a 20×/0.95 objective, 1× tube lens, and oblique brightfield contrast, together with LED mode of the 567 nm channel for LysoTracker Red DND-99 and LED mode of the 385 nm channel for DAPI.

Movies and individual images were exported using Zen Blue software, and subsequent adjustments to image parameters were made with Fiji software. To illustrate lysosome dynamics over time, a series of 16 images was compiled as maximum z-projections for the LysoTracker Red DND-99 channel, capturing all lysosomal events within a 30-min interval. This image set was then duplicated, with one copy processed using the “enhance local contrast” (CLAHE) method and then averaged with the original z-projection image to generate the final image for the LysoTracker channel. The DAPI and oblique channels were represented using the image from the last time point.

#### Live-cell imaging of 2D TE differentiation using Nanolive holotomography coupled with wide-field fluorescence

2D TE differentiation was monitored in real time by refractive index (RI)-based 3D holotomography combined with wide-field fluorescence microscopy (3D Cell Explorer, Nanolive, Switzerland) using a 60× objective with a depth of field of 30 μm. LysoTracker Red DND-99 was used for lysosome tracking (Cy5 channel). The Cy5 channel was activated concurrently with the RI channel to facilitate live-cell imaging. To monitor the 2D TE differentiation process, nPSCs were seeded in a 35-mm µ-Dish (ibidi) containing PXGL medium. After 24 h, the medium was replaced with either N2B27 containing Y or N2B27 supplemented with 2% DMSO and Y. Prior to live-cell imaging, the Nanolive microscope was maintained at 37 °C and 5% CO_2_. Once environmental conditions were established, images were acquired using STEVE software (Nanolive) to generate an RI-based z-stack and a Cy5 track for time-lapse analysis. Images were acquired every 1 min for the RI and Cy5 channels, comprising 96 slices for the RI channel and a single shot for the Cy5 channel. All slices were displayed as maximum z-projections, and image parameters were adjusted using Fiji software. To characterize the apical and basolateral surfaces, top and bottom slices (containing the best focus) from the RI channel were chosen for analysis. The CLAHE method was used as described above to enhance the local contrast of lysosomes.

### TEM

Cells were fixed with 2.5% glutaraldehyde in 0.1 M sodium cacodylate buffer (pH 7.4) at 4 °C (Electron Microscopy Science). Within 24 h, the samples were rinsed three times with fresh 0.1 M cacodylate buffer (pH 7.4) at room temperature for 5 min. Cells were post-fixed using 1% osmium tetroxide (Electron Microscopy Science) in 1.5% potassium ferrocyanide for 1 h at room temperature, followed by 2% osmium tetroxide in water for 30 min at room temperature, and rinsed three times with water for 5 min after each incubation. Preparations were incubated with 1% uranyl acetate at 4 °C for 12 h. Subsequently, the cells were dehydrated in an ethanol series (50%, 70%, 80%, 90%, and 100%) followed by 100% acetone and infiltrated in 3:1, 1:1, and 1:3 mixtures of acetone/Epon resin (Electron Microscopy Science). Finally, the samples were embedded in pure Epon resin and cured for 72 h at 60 °C. Ultrathin sections of 70 nm were prepared using a UC6 ultramicrotome (Leica Microsystems), mounted on copper grids, and stained with 3% lead citrate (Electron Microscopy Science) for 2 min. Micrographs were acquired using a Titan ST transmission electron microscope (Thermo Fisher Scientific) operating at 300 kV.

### RNA library preparation for bulk RNA-seq and ATAC-seq of 2D TE differentiation

RNA was extracted using the QIAGEN RNeasy kit (Cat# 74104) and quality checked with Qubit and Bioanalyzer 2100 instruments. RNA was outsourced to Novogene for additional QC, stranded library construction, and sequencing, with an output of 9 GB raw data. ATAC-seq libraries were constructed using a commercial kit from Diagenode (Cat# C01080002) in accordance with the manufacturer’s recommendations. Ready-to-sequence libraries were sequenced by Novogene, Inc.

### Bulk RNA-seq data analysis of 2D TE differentiation

Quality control, alignment, gene annotation, and read count quantification were performed by Sequentia Inc. For public RNA-seq datasets, we downloaded the raw data and performed de novo analysis to obtain raw counts for the samples using the same pipeline as Sequentia. Batch effects were removed using ComBat. For global analyses, we considered only genes with a count of 15 or higher in at least two samples. Principal component, differential expression, and cluster analyses were performed using log_2_ expression values computed with custom scripts, in addition to the Bioconductor package DESeq2 (v1.38.3). For PCA of the RNA-seq data, plotPCA() in the DESeq2 package was used to normalize the counts and compute the PCA data. PCA data were plotted with the ggplot2 (v3.5.1) package in R (http://ggplot2.org). Gene density contributing to the PCA plots was calculated using kernel density estimation. Heatmaps were plotted with the pheatmap (v1.0.12) package in R using Z-scores. Log_2_ expression values were used to construct dotplots of marker expression.

### Bulk ATAC-seq data analysis of 2D TE differentiation

ATAC-seq data were preprocessed using NGmerge to merge paired-end reads and trim adapters. Cleaned reads were aligned to the human genome assembly (hg38) using Bowtie 2 (v2.3.4.1) with default settings, except for the options “-X 2000 --no-unal --very-sensitive.” Blacklist regions from the hg38 genome were excluded from the analysis. The resulting BAM files were converted to read coverage files in bigWig format using deepTools. Peaks were called using MACS2 (v.2.1.1.20160309) with the options “--nomodel -f BAMPE --keep-dup all” in addition to the default settings. Peak annotation was performed using ChIPseeker to identify associated genomic features. The resulting peak intensity matrix was normalized to counts per million (CPM) and log_2_-transformed for PCA. The PCA plot was generated using the ggplot2 package in R, visualizing normalized ATAC-seq reads mapped to peaks associated with growth and differentiation marker genes. Heatmaps were generated by normalizing read enrichment within a 5-kb window upstream and downstream of transcription start sites (TSSs) and transcription end sites (TESs) of growth and differentiation marker genes. TF binding sites and footprints were predicted, and motif enrichment was analyzed using HINT-ATAC with the aligned BAM files.

### Smart-seq2 transcriptome profiling and analysis of human embryos

Human embryos were collected from control and ConA-treated groups. For each human embryo, mRNA was isolated and reverse transcribed into cDNA using oligo(dT) primers. Template switching was then used to generate full-length cDNA, and the cDNA was amplified by PCR. After amplification, the cDNA was processed for library construction and sequenced to a depth of 6 Gb to obtain paired-end reads (PE150) on the Illumina HiSeq platform by Annoroad Gene Technology (Beijing, China; www.annoroad.com).

DEGs (log_2_fold change > 0.5, adjusted false discovery rate (FDR) < 0.05) between the two groups were identified using DESeq2 v1.28.1. GO enrichment analysis of DEGs was performed using the enrichGO function in the R package clusterProfiler v4.8.3. Gene set enrichment analysis (GSEA) was performed using GSEA v2.0.14 software (www.broadinstitute.org/gsea/index.jsp). Heatmaps of gene expression were generated with the pheatmap package in R (https://CRAN.R-project.org/package=pheatmap).

### RNA library preparation and transcriptomic analysis for the V-ATPase KD assay

mRNA was purified from total RNA using poly(T) oligo-attached magnetic beads. After fragmentation, first-strand cDNA was synthesized using random hexamer primers, followed by second-strand cDNA synthesis. After end repair, A-tailing, adapter ligation, size selection, amplification, and purification, the library was examined with a Qubit instrument and real-time PCR for quantification and with a Bioanalyzer to detect the size distribution.

After library quality control, libraries were pooled on the basis of the effective concentration and targeted data amount, and then subjected to Illumina sequencing.

Raw fastq reads were processed using FASTQ software to remove reads that contained adapters, reads that contained poly-Ns, and low-quality reads. At the same time, the Q20, Q30, and GC content values of the clean data were calculated. All downstream analyses were performed using clean, high-quality data.

The reference genome and gene model annotation files were downloaded from the genome website. HISAT2 (v2.2.1) was used to build an index of the reference genome and to align the paired-end clean reads to the reference genome. HISAT2 uses the gene model annotation file to create splice-aware alignments, providing better alignment accuracy compared with other non-splice-aware alignment tools. The mapped reads of each sample were assembled into full-length transcripts using StringTie (v2.2.3).^88^

featureCounts (v2.0.6) was used to count the number of reads mapped to each gene, and the FPKM (fragments per kilobase of transcript per million reads) value of each gene was calculated from the gene length and the number of mapped reads. Differential expression analysis for two conditions/groups was performed using the DESeq2 R package (v1.42.0). The resulting *P* values were adjusted using the Benjamini–Hochberg method to control the FDR. The criteria for significant differential expression were *P*_adj_ ≤ 0.05 and |log_2_(fold change)| ≥ 1.

GO enrichment analysis of DEGs was performed using clusterProfiler (v4.8.1), correcting for gene length bias. GO terms with corrected *P* values < 0.05 were considered significantly enriched in the DEGs. We used the clusterProfiler R package to test the statistical enrichment of KEGG pathways (http://www.genome.jp/kegg/) in the DEGs.

To examine the overlap and functional relevance of gene expression changes between ConA-treated human embryos and siRNA-mediated KD of ATP6 genes (si*ATP6V0A4*, and si*ATP6V1B1*) in vitro, we focused on the top 3000 most variably expressed genes that were also differentially expressed between treatment and control groups from each dataset. A Venn diagram was generated using the VennDiagram package to visualize shared and unique genes among the three conditions. A heatmap showing the log_2_(fold change) values of these overlapping genes was created with pheatmap, highlighting selected lysosome-related genes to underscore their biological significance. KEGG pathway enrichment of the shared genes was analyzed using clusterProfiler, and the top-enriched pathways (FDR ≤ 0.1) were visualized as bar plots using ggplot2.

### WGCNA

WGCNA (v1.73)^89^ was performed in R (v4.3.1) using 19 RNA-seq samples integrated from three separate datasets: the 2D TE differentiation dataset (*n* = 5 DMSO; *n* = 2 PDA83, this study), the Guo et al. dataset^3^ (*n* = 2 day 3 PDA83; *n* = 2 day 5 PDA83), and the KD study dataset (*n* = 3 siNC; *n* = 3 siATP6V0A4; *n* = 2 siATP6V1B1, this study). Raw counts were intersected across datasets (31,821 genes), and low-abundance genes were filtered (counts ≥ 15 in ≥ 2 samples; 18,955 retained). Expression was variance stabilized with DESeq2 v1.42.0 (vst, blind = TRUE)^90^ and batch corrected with limma v3.58.1 (removeBatchEffect)^91^ using a trait-preserving design. Signed networks were built from the corrected matrix. The soft threshold was the minimal power that achieved a signed scale-free fit *R*^2^ ≥ 0.8 (power = 14). Modules were detected with blockwiseModules (networkType = “signed”, TOMType = “signed”, corType = “bicor”, minModuleSize = 30, mergeCutHeight = 0.25). Module–trait associations were Pearson correlations between eigengenes and dummy-encoded traits (*P* from corPvalueStudent). Topology was summarized with TOMsimilarityFromExpr (power = 14). Functional enrichment was performed using clusterProfiler v4.10.1^92^ with enrichGO for Biological Process (BH-adjusted, *P* ≤ 0.05, *q* ≤ 0.2) and enrichKEGG for pathway analysis (BH-adjusted, *P* ≤ 0.05, *q* ≤ 0.2).

### scRNA-seq library construction and sequencing

For scRNA-seq of PALLY and PALLY + 0.5% DMSO blastoids, we collected pre-implantation blastoid structures and pooled them into a 10-cm dish containing N2B27 medium with Y. We selected 150–250-µm blastoids under a dissection microscope, washed them with PBS, and transferred them to a 1.5-mL Eppendorf tube containing TrypLE and DNase I. These structures were placed in a 37 °C mixer and agitated at 800 rpm for 15 min. The dissociated blastoids were passed through a 40-µm strainer and stained for the TE marker TROP2. To capture a sufficient number of PE cells, we used FACS to deplete TROP2^+^ cells. Similarly, we digested post-implantation PALLY and PALLY + 0.5% DMSO blastoids with TrypLE, counted the cells, and proceeded with single-cell library preparation. For scRNA-seq of DMSO-only D6 blastoids, the structures were collected as described above. Single-cell dissociation was performed using TrypLE and DNase I at 37 °C in a dish, with pipetting every 3 min to facilitate cell separation. The resulting cell suspension was then passed through a 40-µm strainer. A similar procedure was followed for DMSO-only D10 attached blastoids. We then used the 10× Chromium Single Cell 3′ Solution v2 or v3.1 for library preparation, targeting 5000 cells per reaction and adhering to the manufacturer’s protocol. Ready-to-sequence libraries were sent to Novogene for sequencing.

### scRNA-seq data analysis

Raw FASTQ files underwent standard quality control (QC) procedures with FastQC v0.11.9 and BBDuk v35.85, including trimming low-quality bases, removing adapter sequences, and filtering low-quality reads with parameters of a minimum 35-bp read length and a quality score of 25. Using STARsolo, high-quality reads were mapped against the reference human genome (GRCh38/Ensembl release 104), resulting in matrices of raw counts. An additional QC step in R filtered out cells with minimal counts across genes and those with high percentages of mitochondrial gene expression. The matrices were normalized using the scaling normalization technique in the Batchelor R package. The Scran R package facilitated dimensionality reduction, graph-based cell clustering, cluster visualization, and cluster marker gene identification. Clusters were annotated on the basis of recognized publications and curated lists of cell-type marker genes. Doublet detection incorporated two strategies from the scDblFinder R package (https://bioconductor.org/packages/release/bioc/html/scDblFinder.html). The initial method identified doublets as clusters situated between two distinct clusters in terms of expression profiles. The secondary approach involved simulating doublets from expression data and using a classifier to pinpoint doublet cells.

Datasets were integrated following previously reported guidelines.^8^ Published datasets^8,35,36,38^ were downloaded, processed, and transformed into count expression matrices. These matrices underwent filtration steps, considering parameters such as mitochondrial counts and gene counts. Subsequent processes, such as data normalization and integration, combined functions and tools from the Scran, Batchelor, and SeuratWrappers R packages. The results included graph-based clustering formed on the Mutual Nearest Neighbors (MNN) low-dimensional coordinates and a UMAP visualization generated from the leading 20 principal components. The identification of cluster markers and annotations was consistent with previously described techniques. Enrichment analyses leveraged the KEGG and GO databases, examining the marker gene lists for each cluster. Pathways and terms were evaluated using hypergeometric tests with FDR corrections, and significant findings were highlighted. The pathview package v1.30.1 was used to visually represent gene expression within specific functional pathways.

### Reference-based cell-type annotation of the scRNA-seq dataset

Pre-implantation day 6 cells were annotated using a previously established framework.^40^ In brief, cells were projected onto a reference atlas comprising six public datasets. To mitigate sparsity, cells were aggregated into metacells using miloR.^93^ Metacells were rescaled and cosine-normalized for alignment with the reference, then integrated using MNN in the Batchelor package.^94^ Cell identities and stages were predicted using Support Vector Machine-trained models. Low-confidence matches (Spearman correlation < 0.5) and outliers were excluded.

### RNA velocity analysis

Spliced and unspliced expression matrices were generated from the pre-implantation D6 aligned BAM file using the velocyto pipeline^95^ with the GRCh38 genome annotation (v2024-A) and a repeat mask (UCSC GRCh38) to exclude repetitive elements. Quantification was restricted to a whitelist of QC-passed, doublet-free barcodes. The processed single-cell dataset was analyzed using scVelo.^96^ Spliced and unspliced abundance values were normalized and smoothed using nearest-neighbor moments. RNA velocity was estimated using the dynamical model, which infers full splicing kinetics for each gene without assuming steady-state dynamics. Latent time was inferred to reconstruct temporal dynamics.

### SCENIC analysis

Gene regulatory networks were inferred, and regulons were predicted using the pySCENIC framework.^97^ Co-expression modules were inferred using the GRNBoost2 algorithm on the basis of a curated list of human TFs. These modules were pruned for direct targets by identifying enriched *cis*-regulatory motifs (cisTarget) using the hg38 refseq_r80 v10 ranking database (10-kb search space) and corresponding motif-to-factor annotations (available at https://resources.aertslab.org/). Regulon activity was quantified using AUCell and projected into UMAP and t-SNE embeddings to visualize regulatory heterogeneity. The final integrated dataset, containing gene expression, regulon activity scores, and embeddings, was exported as a SCope-compatible LOOM file. Regulons with significantly higher activity in specific clusters were identified using the Wilcoxon rank-sum test. The top 5 most specific regulons for each group, ranked by statistical significance, were visualized in a heatmap.

### Real-time quantitative PCR

Total RNA was extracted using a QIAGEN kit (Cat# 74104) following the manufacturer’s protocol and quality checked with a NanoDrop spectrophotometer. RNA (1 µg) was reverse transcribed to cDNA using the Bio-Rad iScript cDNA synthesis kit (Cat# 1708890) or the Thermo SuperScript III First-Strand Synthesis System (Cat# 18080400) following the manufacturer’s recommendations. Subsequent qPCR was performed on the Bio-Rad CFX384 detection system using Bio-Rad SsoAdvanced SYBR polymerase (Cat# 1725270). Data analysis was performed in Excel using the ΔCt method with actin as the reference gene. To extract mRNA from mouse embryos, five embryos were placed into each 0.2-mL PCR tube. For each tube, 4 µL of lysis buffer containing 0.2% Triton X-100, RNase inhibitor, dNTPs, and oligo(dT) primers was added. Samples were immediately incubated at 72 °C for 3 min to facilitate RNA release, and cDNA was then synthesized using SuperScript II reverse transcriptase. All oligo sequences used in this study are listed in Supplementary information, Table S2.

### Western blot

Cells were lysed using RIPA lysis buffer (150 mM NaCl, 1.0% Triton X-100, 0.5% Na-deoxycholate, and 50 mM Tris, pH 8.0) with protease and phosphatase inhibitor cocktails (Sigma, Cat# 11836170001; Life Technologies, Cat# 78440). Samples were prepared using 2× Laemmli buffer (Bio-Rad, Cat# 1610747) according to the manufacturer’s instructions, and 10 µg of each sample was separated on 4%–12% Bis-Tris Plus gels (Thermo Fisher, Cat# NW04125BOX) and transferred to nitrocellulose membranes. Membranes were blocked in 5% skim milk or BSA in 0.1% TBS-Tween 20, according to antibody instructions, for 1 h at room temperature and then incubated overnight at 4 °C with primary antibodies. After washing, membranes were incubated with secondary antibodies for 1 h at room temperature. Chemiluminescent HRP substrate (Thermo Fisher, Cat# 34076) was used for signal detection. Protein expression was analyzed using Fiji software.

### In vitro blastoid attachment assay

The in vitro attachment assay was performed according to a previously established protocol.^4,43,98^ On day 5 or 6, well-formed blastoids were manually picked using a stereomicroscope (Leica M205 FCA). An 8-well µ-slide chamber (ibidi, Cat# 80827) was pre-coated with Matrigel diluted in cold DMEM/F12 (1:30) for 30 min prior to use. Subsequently, 3–5 blastoids were introduced into each chamber containing IVC1 medium and incubated at 37 °C with 5% CO_2_ and 5% O_2_. The IVC1 medium consisted of Advanced DMEM/F12 (Thermo, Cat# 12634-010) supplemented with 20% heat-inactivated FBS (Thermo, Cat# 30044333), 2 mM L-GlutaMAX (Thermo, Cat# 35050-061), 0.5% penicillin–streptomycin (Thermo, Cat# 15140-122), 1% ITS-X (Thermo, Cat# 51500-056), and 1% sodium pyruvate. The medium also included specific additives: 8 nM β-estradiol (Sigma-Aldrich, Cat# E8875), 200 ng/mL progesterone (Sigma-Aldrich, Cat# P0130), 25 μM N-acetyl-L-cysteine (Sigma-Aldrich, Cat# A7250), and ROCK inhibitor (Selleckchem, Cat# S1049). After 48 h, the medium was replaced with IVC2, which mirrored the composition of IVC1 but substituted 20% FBS with 30% KnockOut Serum Replacement. Structures were then cultured for an additional 48 h. On day 4, the medium was collected for testing with a commercial hCG pregnancy kit, and the structures underwent further downstream analysis. Immunofluorescence assays were performed on day 4 post attachment.

### PKC and RA pathway assays

For the pan-PKC inhibitor assay, 1 mM Gö6983 (Selleckchem) was added during the compaction stage under the above-mentioned conditions. The structures were collected on day 6 and subjected to immunofluorescence analysis to check the expression of GATA3, YAP1, and OCT4. For isozyme-specific peptide inhibitors against PKCα/β, PKCζ, and PKCη isoforms, 100 nM Gö6976 (Cell Signaling), 10 mM of PKCζ pseudosubstrate inhibitor (Life Technologies), and 10 mM PKCη pseudosubstrate inhibitor (Millipore) were added during the compaction stage, and cavity formation was examined under the microscope. For the RA pathway assay, SC9N nPSCs were plated as described for the TE differentiation protocol. After 24 h of plating, the medium (PXGL + Y) was replaced with N2B27 only or N2B27 supplemented with 5 mM RA, 2% DMSO, AGN193109 (Santa Cruz Biotechnology, Cat# sc-210768; 50 nM, 500 nM, or 5000 nM), or LY2955303 (Sigma-Aldrich, Cat# SML2183; 0.1 mM or 1 mM); 2% DMSO, together with AGN193109 and LY2955303, was also tested alongside the other conditions. After 96 h of daily medium changes, the cells were harvested for flow cytometry analysis of TROP2 expression. Since the RA inhibitor drugs were prepared in DMSO, the effective DMSO concentration from these drugs did not exceed 0.1%.

### Pharmacological and siRNA-mediated inhibition of V-ATPase during TE cyst formation

To inhibit V-ATPase activity, cells were treated with BafA1 and ConA at the specified concentrations. Cells were prepared following the blastoid differentiation protocol. In brief, cells were plated in AggreWell plates using N2B27 medium supplemented with Y. Once compact aggregates had formed, they were treated with 3% DMSO along with BafA1 and ConA and incubated for 2–4 days (see Fig. 5b for the experimental scheme). Cavitation efficiency was assessed on day 5. Both BafA1 and ConA were dissolved in DMSO, and DMSO was used as the vehicle control. The final DMSO concentration was 0.01%.

For siRNA-mediated loss-of-function studies, cells were prepared as described for the TE differentiation assay. After 24 h, cells were transfected with esiRNA targeting *ATP6V0A4* (Sigma-Aldrich, Cat# EHU132531), siRNA targeting *ATP6V0A4* (Thermo, Cat# s27050) or *ATP6V1B1* (Sigma-Aldrich, Cat# EHU017741), or siRNA targeting *ATP6V1B1* (Thermo, Cat# s1796) using Lipofectamine RNAiMAX (Invitrogen, Cat# 13778150) or MessengerMax (Invitrogen, Cat# LMRNA015) reagents. On the following day, fresh N2B27 medium supplemented with 2% DMSO was added, and the cells were cultured for an additional 48 h, with daily replenishment of medium containing 2% DMSO. To rescue the KD genes, overexpression constructs expressing *GFP-ATP6V0A4* or *GFP-ATP6V1B1* mRNA were introduced into the respective KD backgrounds and subjected to downstream analysis as described above.

### mRNA IVT synthesis

Plasmids for *GFP-ATP6V0A4* and *GFP-ATP6V1B1* IVT mRNA synthesis were customized from GenScript. Following restriction digestion, capped mRNA was synthesized from the linearized plasmid template using the HighYield T7 ARCA mRNA Synthesis Kit (m5CTP/Psi-UTP) from Jena Bioscience (Cat# RNT-103). Residual template DNA was removed by TURBO DNase treatment for 15 min. The mRNA was then subjected to poly(A) tailing using the Poly(A) Tailing Kit from Invitrogen (Cat# AM1350) for 1 h, followed by phosphatase treatment with Quick CIP from NEB (Cat# M0525S) for 3 h to remove 5′-phosphate groups. The synthesized mRNA was purified using the Monarch Spin RNA Cleanup Kit from NEB (Cat# T2040L), and the purified mRNA was aliquoted and stored at −80 °C until use.

### Effect of loss of function of V-ATPases on human and mouse embryo cavitation

Human morulae in the ConA-treated group were cultured in G-2 medium (G2 PLUS, Vitrolife Sweden AB) supplemented with 1 nM ConA (MCE, HY-N1724) for a minimum of 24 h. By day 5 post in vitro fertilization, embryos that had not undergone cavitation were classified as arrested morulae, whereas control embryos had successfully progressed to the blastocyst stage with cavitation. Blastocysts from the control group and arrested morulae from the ConA-treated group were then collected for subsequent analysis.

PN5-stage mouse zygotes were collected 24 h post hCG administration and cultured in KSOM medium (Millipore) under mineral oil in a humidified incubator at 37 °C with 5% CO₂. Zygotes were cultured in KSOM until the 8-cell stage, then transferred to medium containing 1 µM BafA1 or 1 nM/10 nM ConA and maintained until embryonic day 4.5 (E4.5 dpc). Embryos were imaged using a Nikon ECLIPSE Ts2 microscope with ImageView software, and images were analyzed using ImageJ. Cavity size was quantified using a previously published method.^99^ In brief, the periphery of each embryo and the cavity lining were manually traced using the polygon selection tool, and their respective areas were measured using the Fiji ROI function. Cavity size was calculated as the percentage of the cavity area relative to the total embryo area. A schematic depicting the cavity size calculation is included as an inset in the corresponding figure.

For siRNA-mediated loss-of-function studies of mouse embryo cavitation, a pooled siRNA solution (final concentration, 20 µM) was microinjected into the zygote cytoplasm using an Eppendorf FemtoJet microinjector and Narishige micromanipulators. The microinjection procedure was performed under a stereomicroscope to ensure precise delivery. Following microinjection, zygotes were cultured in a humidified incubator at 37 °C with 5% CO₂ until they reached the blastocyst stage. Embryo viability and development were monitored periodically to assess the effects of siRNA treatment. The sequences of the siRNAs used in this study are listed in Supplementary information, Table S2.

### Mass spectrometry analysis

For mass spectrometry (MS) analysis, nPSCs were differentiated using the 2D TE assay as described previously. Cells were collected and washed three times with ice-cold PBS and lysed in a buffer containing 0.5% SDS dissolved in 50 mM triethylammonium bicarbonate, supplemented with a protease inhibitor cocktail (Sigma-Aldrich, Cat# 11873580001) and benzonase (5 U/mL, Merck Millipore). Cell lysates were centrifuged at 10,000× *g* for 10 min. The protein content of the resulting supernatants was determined using a NanoDrop spectrophotometer (Thermo Fisher Scientific).

Approximately 20 µg of protein was digested using the SP3 approach following a published protocol.^100^ The digest was desalted, dried, and resuspended in 0.1% FA. Approximately 200 ng of peptide mixture per sample was analyzed using a timsTOF Pro 2 mass spectrometer coupled to a nanoElute liquid chromatography system (Bruker Daltonik GmbH, Germany). Samples were injected directly into an RP-C18 Aurora emitter column (75 µm i.d. × 250 mm, 1.6 μm, 120 Å pore size; Ion Opticks, Australia) using a one-column separation method. A 75-min run was established using mobile phase A (0.1% formic acid in water) and mobile phase B (0.1% formic acid in acetonitrile): 2%–25% B for 60 min, 25%–37% B for 15 min, ramping from 37% to 95% B over 5 min, and maintaining 95% B for 5 min. The column temperature was set to 50 °C, and the flow rate was maintained at 250 nL/min. Eluted peptides were introduced into the mass spectrometer via a CaptiveSpray nano-electrospray ion source (Bruker Daltonik GmbH) with an electrospray voltage of 1.5 kV. The ion source temperature was set to 180 °C, and the dry gas flow rate was 3 L/min. Samples were analyzed using the diaPASEF scheme, consisting of 24 cycles including a total of 48 mass-width windows (13 Da (*m/z*) from *m/z* 400 to *m/z* 1000) and a TIMS scan range from 0.64 Vs/cm^2^ to 1.45 Vs/cm^2^ (1/K0). Collision energy increased linearly from 20.01 eV at 0.6 Vs/cm^2^ (1/K0) to 52.00 eV at 1.35 Vs/cm^2^ (1/K0). The scan range for MS and MS/MS spectra was set to 100–1700 *m/z*. TIMS ramp time and accumulation time were set to 100 ms.

### AFM measurements

Following removal of the zona pellucida, mouse embryos were immobilized in 3% agarose within a 35-mm cell culture dish. After embryos were secured, samples were transferred to a JPK NanoWizard BioScience SPM system for AFM measurements.

The instrument was equipped with a Bruker SAA-SPH-1UM probe containing a silicon nitride cantilever with spherical tip geometry. Prior to data acquisition, probe parameters were defined, including a nominal tip radius of 1 µm and a spring constant of ~0.25 N/m. The exact spring constant was calibrated in situ using the thermal noise method implemented in JPK software to ensure accurate force measurements. For mechanical modeling, a Poisson’s ratio of 0.45 was applied, which is commonly used for soft biological samples. Mechanical characterization was performed by acquiring force-distance curves across the embryo surface. Measurements were conducted using a trigger force of 5 pN and a total *Z*-axis travel range of 15 µm. Both approach and retraction phases were carried out at a constant loading speed of 5 µm/s. Each measurement was saved as a JPK force file containing complete approach and retract curves, which were subsequently used for quantitative analysis of embryo mechanical properties.

### MS data analysis

The diaPASEF data were analyzed using the directDIA approach in Spectronaut software (v18.6). In brief, the MS data (.d files) and UniProt human proteome sequences (20,386 sequences) were loaded into Spectronaut. The default settings for database matching consisted of the following parameters: full trypsin cleavage, peptide lengths between 7 and 52 amino acids, and a maximum of two missed cleavages. In addition, lysine and arginine (KR) were used as special amino acids for decoy generation, and N-terminal methionine was removed during pre-processing of the protein database. In the directDIA workflow, data extraction was based on maximum intensity in both MS1 and MS2 spectra with relative mass tolerances of 10 ppm and 20 ppm, respectively. The retention time window of the extracted ion chromatograms was set dynamically, with a correction factor of 0.5 to enhance specificity. Biognosys default settings were applied for both identification and quantification. To determine differential protein abundance between samples, an unpaired *t*-test assuming equal variance was performed, followed by group-wise multiple-testing correction and clustering. Proteins with a fold change > 1.5 and a *q*-value < 0.05 were considered differentially expressed.

### Statistical analysis and reproducibility

Results were documented as means and standard deviations from at least three independent experiments (unless otherwise specified in the respective figure legends or within the figures). Comparisons between the two sets were analyzed using the Student’s *t*-test. Experiments involving three or more groups were examined by one-way or two-way ANOVA, followed by the indicated post hoc test. A *P*-value < 0.05 was considered statistically significant. All statistical analyses were performed in GraphPad Prism 10.

## Supplementary information

Supplementary information, Fig. S1

Supplementary information, Fig. S2

Supplementary information, Fig. S3

Supplementary information, Fig. S4

Supplementary information, Fig. S5

Supplementary information, Fig. S6

Supplementary information, Fig. S7

Supplementary information, Fig. S8

Supplementary information, Fig. S9

Supplementary information, Fig. S10

Supplementary information, Fig. S11

Supplementary information, Fig. S12

Supplementary information, Fig. S13

Supplementary information, Video S1

Supplementary information, Video S2

Supplementary information, Video S3

Supplementary information, Video S4

Supplementary information, Video S5

Supplementary information, Video S6

Supplementary information, Video legends

Supplementary information, Table S1

Supplementary information, Table S2

## Data Availability

scRNA-seq, bulk RNA-seq, and bulk ATAC-seq were deposited at the Gene Expression Omnibus (GEO) repository. The accession numbers are as follows: scRNA-seq datasets (GSE237669 and GSE316552), bulk RNA-seq (GSE236961 — 2D TE assay; GSE291369 — human embryo data), and bulk ATAC-seq (GSE239744). The bioproject accession number for *ATP6V0A4* and *ATP6V1B1* KD datasets is PRJNA1240850. The MS proteomics data have been deposited to the ProteomeXchange Consortium via the PRIDE partner repository with the dataset identifier PXD069054.
