# Supplementary material for: A single small molecule-based human embryo model reveals V-ATPase requirement in mammalian blastocyst cavitation
Source: Cell Res. 2026 Apr 6;36(7):475–98. doi: 10.1038/s41422-026-01239-3 (PMC13287814; doi:10.1038/s41422-026-01239-3)
Supplement: Supplementary file 1 — Supplementary information, Fig. S1 [file 41422_2026_1239_MOESM1_ESM.pdf]

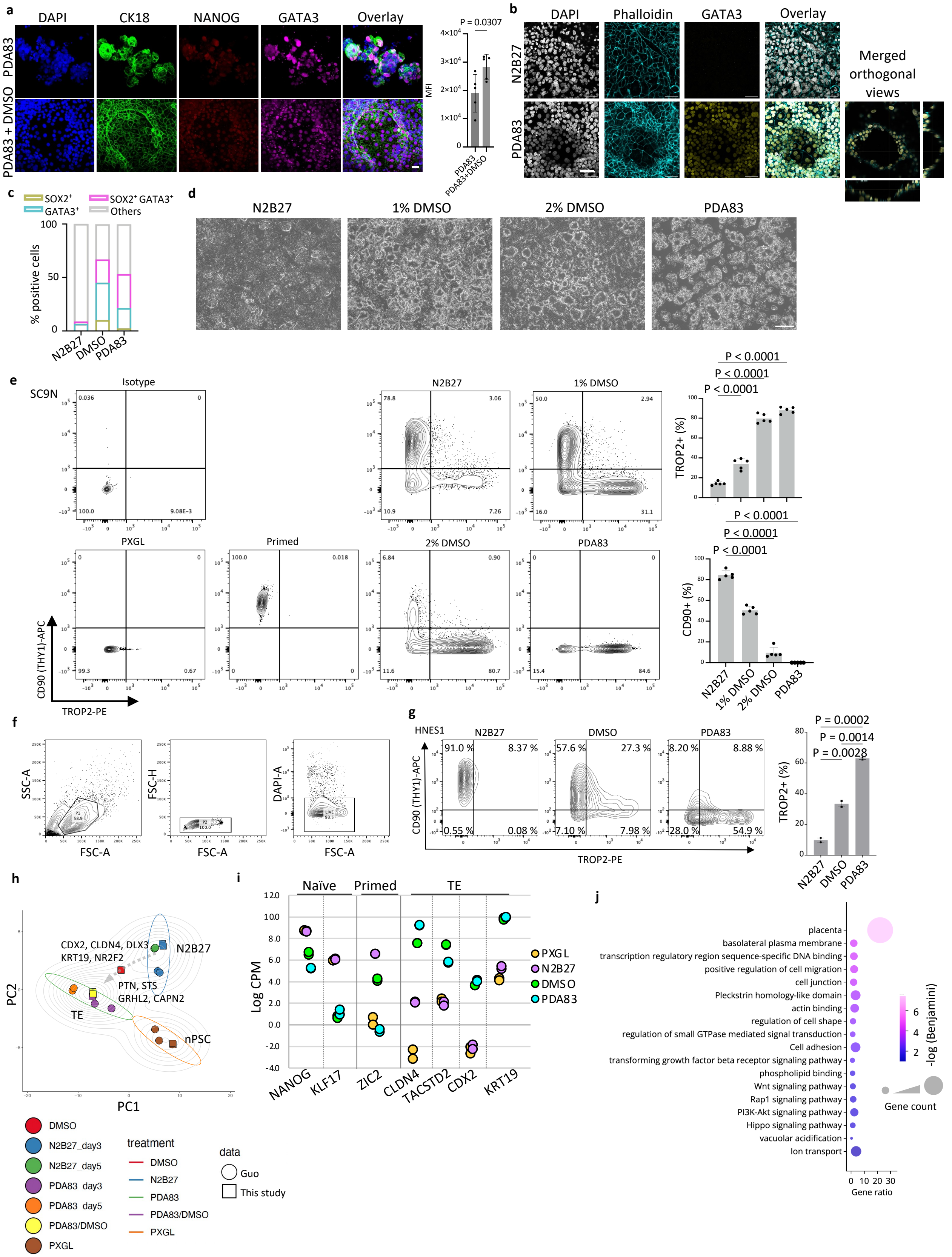
a
DAPI
CK18
NANOG
GATA3
Overlay
PDA83
PDA83 + DMSO
P = 0.0307
MFI
PDA83
PDA83+DMSO
b
DAPI
Phalloidin
GATA3
Overlay
N2B27
PDA83
Merged orthogonal views
c
SOX2+
SOX2+ GATA3+
GATA3+
Others
% positive cells
N2B27
DMSO
PDA83
d
N2B27
1% DMSO
2% DMSO
PDA83
e
SC9N
Isotype
N2B27
1% DMSO
PXGL
Primed
2% DMSO
PDA83
CD90 (THY1)-APC
TROP2-PE
P < 0.0001
TROP2+ (%)
CD90+ (%)
f
SSC-A
FSC-A
FSC-H
DAPI-A
g
HNES1
N2B27
DMSO
PDA83
91.0 %
8.37 %
57.6 %
27.3 %
8.20 %
8.88 %
0.55 %
0.08 %
7.10 %
7.98 %
28.0 %
54.9 %
CD90 (THY1)-APC
TROP2-PE
P = 0.0002
P = 0.0014
P = 0.0028
TROP2+ (%)
h
CDX2, CLDN4, DLX3
KRT19, NR2F2
N2B27
PTN, STS
GRHL2, CAPN2
TE
nPSC
PC2
PC1
DMSO
N2B27_day3
N2B27_day5
PDA83_day3
PDA83_day5
PDA83/DMSO
PXGL
treatment
DMSO
N2B27
PDA83
PDA83/DMSO
PXGL
data
Guo
This study
i
Naïve
Primed
TE
Log CPM
PXGL
N2B27
DMSO
PDA83
NANOG
KLF17
ZIC2
CLDN4
TACSTD2
CDX2
KRT19
j
placenta
basolateral plasma membrane
transcription regulatory region sequence-specific DNA binding
positive regulation of cell migration
cell junction
Pleckstrin homology-like domain
actin binding
regulation of cell shape
regulation of small GTPase mediated signal transduction
Cell adhesion
transforming growth factor beta receptor signaling pathway
phospholipid binding
Wnt signaling pathway
Rap1 signaling pathway
PI3K-Akt signaling pathway
Hippo signaling pathway
vacuolar acidification
Ion transport
-log (Benjamini)
Gene count
Gene ratio

Fig. S1 DMSO promotes exit of the naïve state and facilitates TE differentiation. **a** Immunofluorescence analysis shows the expression of CK18 (green), NANOG (red), and GATA3 (magenta) in PDA83 and PDA83+2% DMSO conditions (n = 3). Scale bar, 40 μm. The bar graph shows the quantification of CK18 expression in PDA83 and PDA83+2% DMSO conditions (**right**). Data are presented as the mean ± standard deviation of three independent experiments. The Student t-test was used. P value is as indicated. **b** Immunofluorescence analysis shows the staining of Phalloidin (cyan) and GATA3 (yellow) in N2B27 and PDA83 conditions. Scale bar, 50 μm. **c** Quantification of HNES1 immunofluorescence data showing the percentage of the mentioned lineage. **d** Representative brightfield images display the cyst formation under different conditions (n = 5). Scale bar, 400 μm. **e** FACS data displays the population of cells in TROP2 (TE marker) and CD90 (primed state marker) in the SC9N nPSC cell line (n = 3). Data are presented as the mean ± standard deviation from five independent experiments. One-way ANOVA followed by the Dunnett post hoc test was used. P values are as indicated. **f** FACS gating strategy. **g** FACS data displays the population of cells in TROP2 (TE marker) and CD90 (primed state marker) in different conditions (n = 2) in the HNES1 nESC cell line. One-way ANOVA followed by Tukey post hoc test was used. P values are as indicated. **h** PCA plot of integrated bulk RNAseq analysis of nPSC, N2B27, and TE differentiation from this study and previously published data. The dotted arrow indicates the top PCA loading genes (*CDX2, CLDN4, DLX3, KRT19, NR2F2, PTN, STS, GRHL2,* and *CAPN2*) between the N2B27 and DMSO samples. **i** Bulk RNAseq data show the expression pattern of various genes in naïve, primed, and TE states under different culture conditions (n = 2). **j** Gene ontology (GO) analysis of differentially expressed genes between DMSO and N2B27 conditions.
