# Supplementary material for: A single small molecule-based human embryo model reveals V-ATPase requirement in mammalian blastocyst cavitation
Source: Cell Res. 2026 Apr 6;36(7):475–98. doi: 10.1038/s41422-026-01239-3 (PMC13287814; doi:10.1038/s41422-026-01239-3)
Supplement: Supplementary file 2 — Supplementary information, Fig. S2 [file 41422_2026_1239_MOESM2_ESM.pdf]

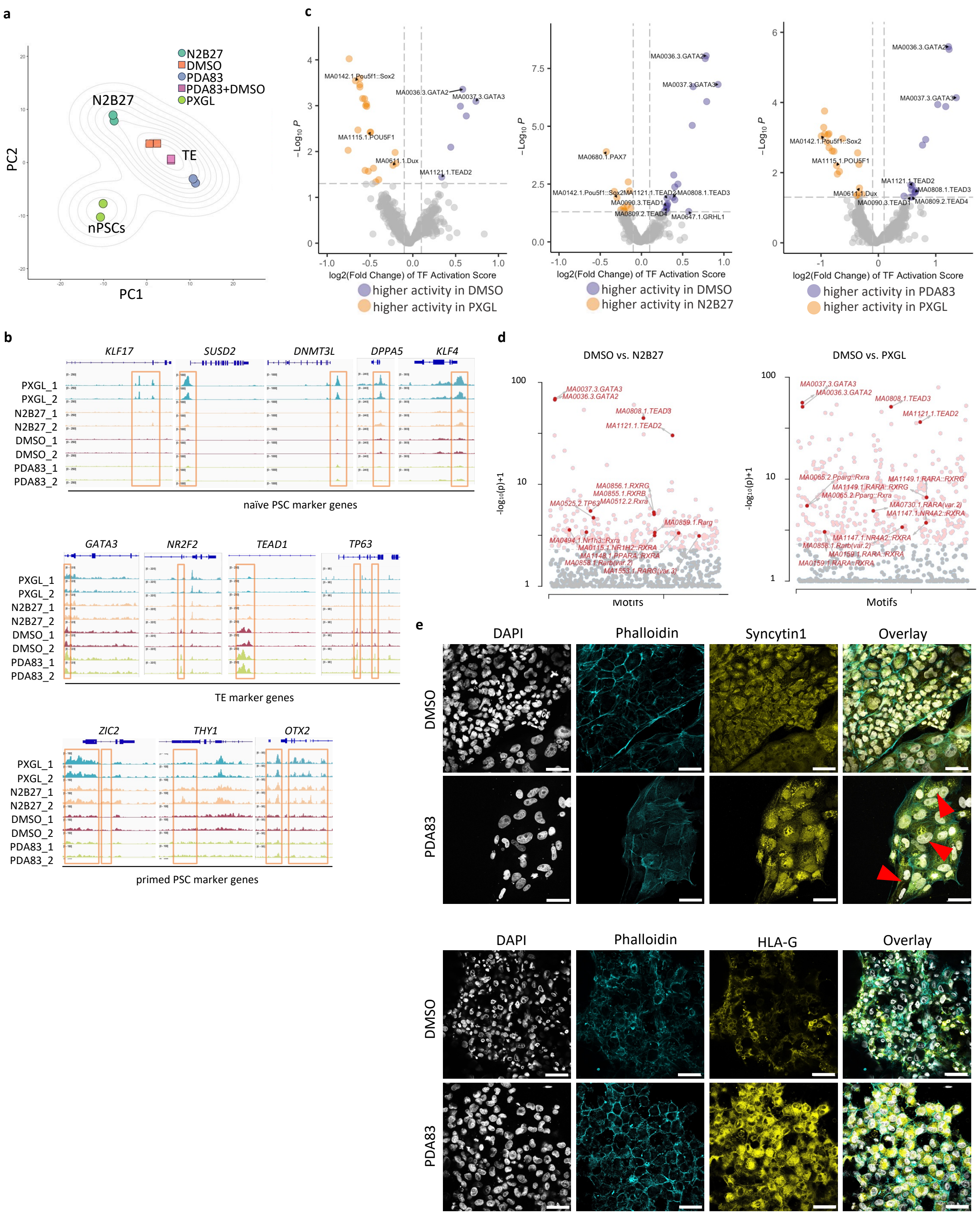
a
N2B27
DMSO
PDA83
PDA83+DMSO
PXGL
TE
nPSCs
PC1
PC2
b
KLF17
SUSD2
DNMT3L
DPPA5
KLF4
PXGL_1
PXGL_2
N2B27_1
N2B27_2
DMSO_1
DMSO_2
PDA83_1
PDA83_2
naïve PSC marker genes
GATA3
NR2F2
TEAD1
TP63
TE marker genes
ZIC2
THY1
OTX2
primed PSC marker genes
c
log2(Fold Change) of TF Activation Score
higher activity in DMSO
higher activity in PXGL
higher activity in N2B27
higher activity in PDA83
d
DMSO vs. N2B27
DMSO vs. PXGL
Motifs
e
DAPI
Phalloidin
Syncytin1
Overlay
HLA-G
DMSO
PDA83

Fig. S2 ATAC-seq analysis reveals the role of DMSO in TE fate. a PCA plot of bulk ATAC-seq data of nPSC, N2B27, and TE differentiation conditions. **b** IGV plots of ATAC-seq signals for marker genes of naïve PSC (**top**), TE (**middle**), and primed PSC (**bottom**). **c** The volcano plots show the comparisons of transcription factor activity across the epigenome of DMSO vs. PXGL (**left**), DMSO vs. N2B27 (**middle**), and PDA83 vs. PXGL (**right**) conditions. **d** Motif enrichment analysis shows the enriched TF motifs in DMSO vs. N2B27 (**left**), and DMSO vs. PXGL (**right**). **e** Immunofluorescence analysis of F-actin (stained by phalloidin in cyan), Syncytin1 (yellow) (**top**), HLA-G (yellow) (**bottom**) under the DMSO and PDA83 conditions (n = 3). Red arrows indicate the multinucleated cells. Scale bar, 50 μm.
