# Supplementary material for: A single small molecule-based human embryo model reveals V-ATPase requirement in mammalian blastocyst cavitation
Source: Cell Res. 2026 Apr 6;36(7):475–98. doi: 10.1038/s41422-026-01239-3 (PMC13287814; doi:10.1038/s41422-026-01239-3)
Supplement: Supplementary file 3 — Supplementary information, Fig. S3 [file 41422_2026_1239_MOESM3_ESM.pdf]

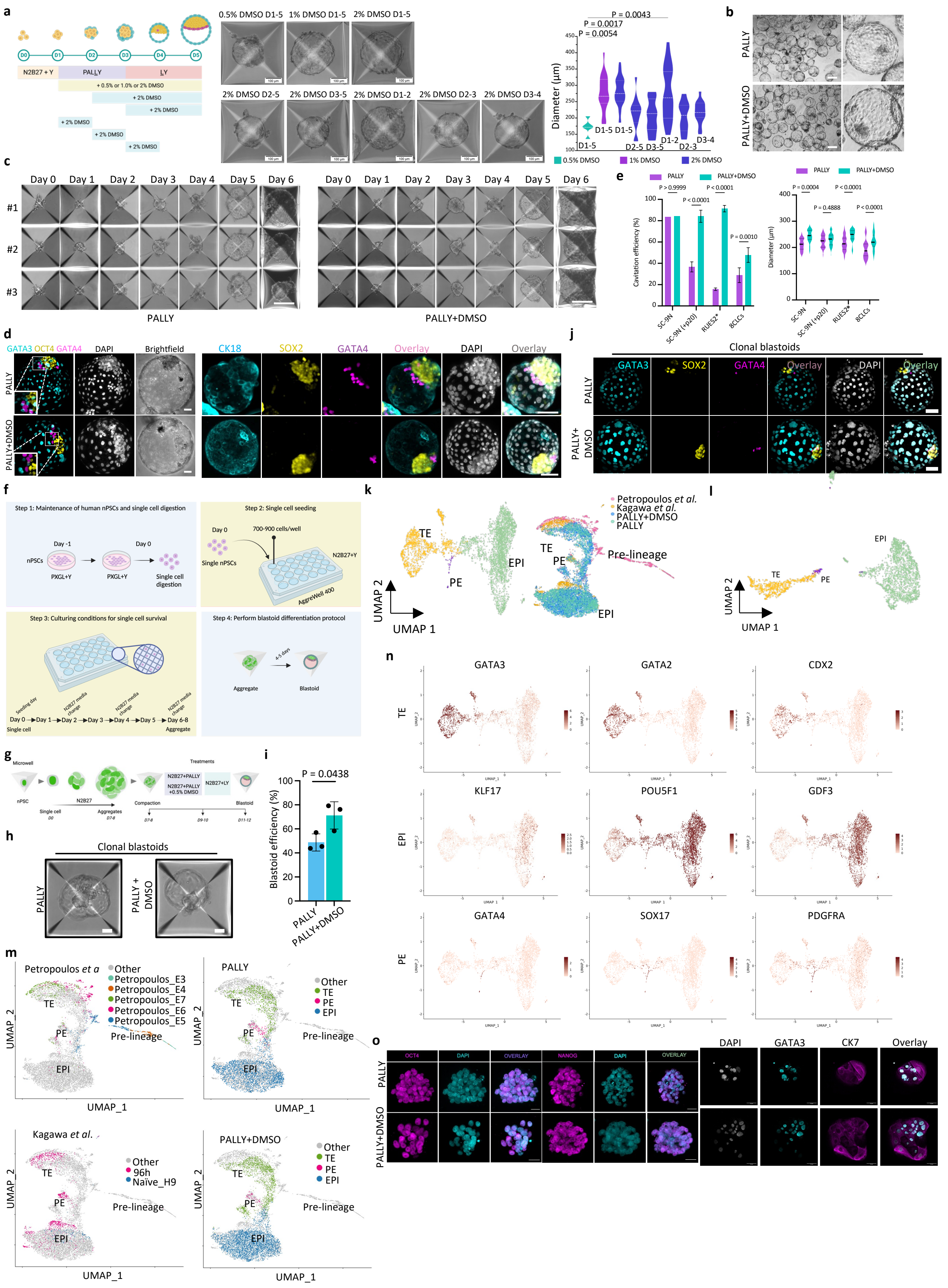
a
N2B27 + Y
PALLY
LY
+ 0.5% or 1.0% or 2% DMSO
+ 2% DMSO
0.5% DMSO D1-5
1% DMSO D1-5
2% DMSO D1-5
2% DMSO D2-5
2% DMSO D3-5
2% DMSO D1-2
2% DMSO D2-3
2% DMSO D3-4
P = 0.0043
P = 0.0017
P = 0.0054
Diameter (µm)
0.5% DMSO
1% DMSO
2% DMSO
b
PALLY
PALLY+DMSO
c
Day 0
Day 1
Day 2
Day 3
Day 4
Day 5
Day 6
#1
#2
#3
PALLY
PALLY+DMSO
e
Cavitation efficiency (%)
Diameter (µm)
SC-9N
SC-9N (+p20)
RUES2*
8CLCs
d
GATA3 OCT4 GATA4
DAPI
Brightfield
CK18
SOX2
GATA4
Overlay
j
Clonal blastoids
GATA3
SOX2
GATA4
Overlay
DAPI
PALLY+ DMSO
f
Step 1: Maintenance of human nPSCs and single cell digestion
Step 2: Single cell seeding
Step 3: Culturing conditions for single cell survival
Step 4: Perform blastoid differentiation protocol
Single nPSCs
700-900 cells/well
AggreWell 400
Aggregate
Blastoid
k
Petropoulos et al.
Kagawa et al.
PALLY+DMSO
PALLY
TE
EPI
PE
Pre-lineage
UMAP 1
UMAP 2
l
g
Treatments
N2B27
Compaction
h
Clonal blastoids
PALLY + DMSO
i
P = 0.0438
Blastoid efficiency (%)
n
GATA3
GATA2
CDX2
KLF17
POU5F1
GDF3
GATA4
SOX17
PDGFRA
m
Petropoulos et al
Other
Petropoulos_E3
Petropoulos_E4
Petropoulos_E7
Petropoulos_E6
Petropoulos_E5
UMAP_1
UMAP_2
Kagawa et al.
96h
Naïve_H9
o
OCT4
DAPI
OVERLAY
NANOG
GATA3
CK7
Overlay

Fig. S3 Effect of DMSO in the PALLY-derived pre-implantation blastoids. a Scheme explains the design of the dose and time window of DMSO exposure in PALLY protocol (**left**). Brightfield images show the formation of blastocyst-like structures under different conditions. Scale bar, 100 μm (**middle**). The violin plot shows the diameter (n = 3). One-way ANOVA analysis was used, and P values are as indicated (**right**). **b** Brightfield images showing the blastocyst-like structures formed in PALLY and PALLY+0.5% DMSO conditions (n = 3). Scale bar, 200 μm. **c** Brightfield images consistently show the blastocyst-like structures in PALLY (**left**) and PALLY+0.5% DMSO (**right**) conditions (n = 3). Scale bar, 200 μm. **d** Immunofluorescence analysis shows the expression of GATA3 (cyan), OCT4 (yellow), and GATA4 (magenta) (left panels) and the expression of CK18 (cyan), SOX2 (yellow), and GATA4 (magenta) (right panels) (n = 3). Scale bar, 60 μm. **e** Comparative analyses of efficiency (**left**) and size (**right**) of the blastoids in different cell types (n = 1 for SC-9N; n = 4 for SC-9N (+p20); n = 4 for RUES2; and n = 3 for 8CLCs). SC-9N (+p20) refers to the SC-9N parental cell line (first column) that has been passaged 20 times. The asterisk denotes that the RUES2 cell line was treated with PALLY+1% DMSO. Data are presented as the mean ± standard deviation. Two-way ANOVA followed by Bonferroni's post hoc test was used, and P values are as indicated. **f** Schematic shows the step-by-step derivation of clonal blastoids from single nPSCs. **g** Schematic depicts the generation of clonal blastoids in PALLY and PALLY+0.5% DMSO conditions. **h** Representative brightfield images showing the cavitated structures in PALLY and PALLY+0.5% DMSO conditions (n = 3). Scale bar, 50 μm. **i** Graph shows the percentage of cavitated structures. Data are presented as the mean ± standard deviation from three independent experiments. A two-tailed t-test was used, and P value is as indicated. **j** Immunofluorescence analysis of the clonal blastoids displays the TE marker (GATA3; cyan), EPI marker (SOX2; yellow), and PE marker (GATA4; magenta) (n = 3). Scale bar, 50 μm. **k** UMAP of the transcriptome of 3244 single cells of the pre-implantation PALLY+DMSO blastoids with cell type annotation (**left**). UMAP projection of integrated datasets showing cells from this study and published studies by Kagawa et al. and Petropoulos et al. (**right**). **l, m** UMAP of the transcriptome of 4687 single cells of the pre-implantation PALLY blastoids with cell type annotations (**l**) and a UMAP projection of the integrated datasets showing cells from this study and previously published reports (**m**). **n** Feature plots of markers of each blastocyst lineage (TE, EPI, and PE) in PALLY+0.5% DMSO condition. **o** Immunofluorescence analysis shows the rederivation of stem cells (top **left**, OCT4; **top right**, NANOG; and **bottom**, GATA3/CK7) in PALLY and PALLY+0.5% DMSO conditions (n = 1). Scale bar, 20 μm.
