# Supplementary material for: A single small molecule-based human embryo model reveals V-ATPase requirement in mammalian blastocyst cavitation
Source: Cell Res. 2026 Apr 6;36(7):475–98. doi: 10.1038/s41422-026-01239-3 (PMC13287814; doi:10.1038/s41422-026-01239-3)
Supplement: Supplementary file 4 — Supplementary information, Fig. S4 [file 41422_2026_1239_MOESM4_ESM.pdf]

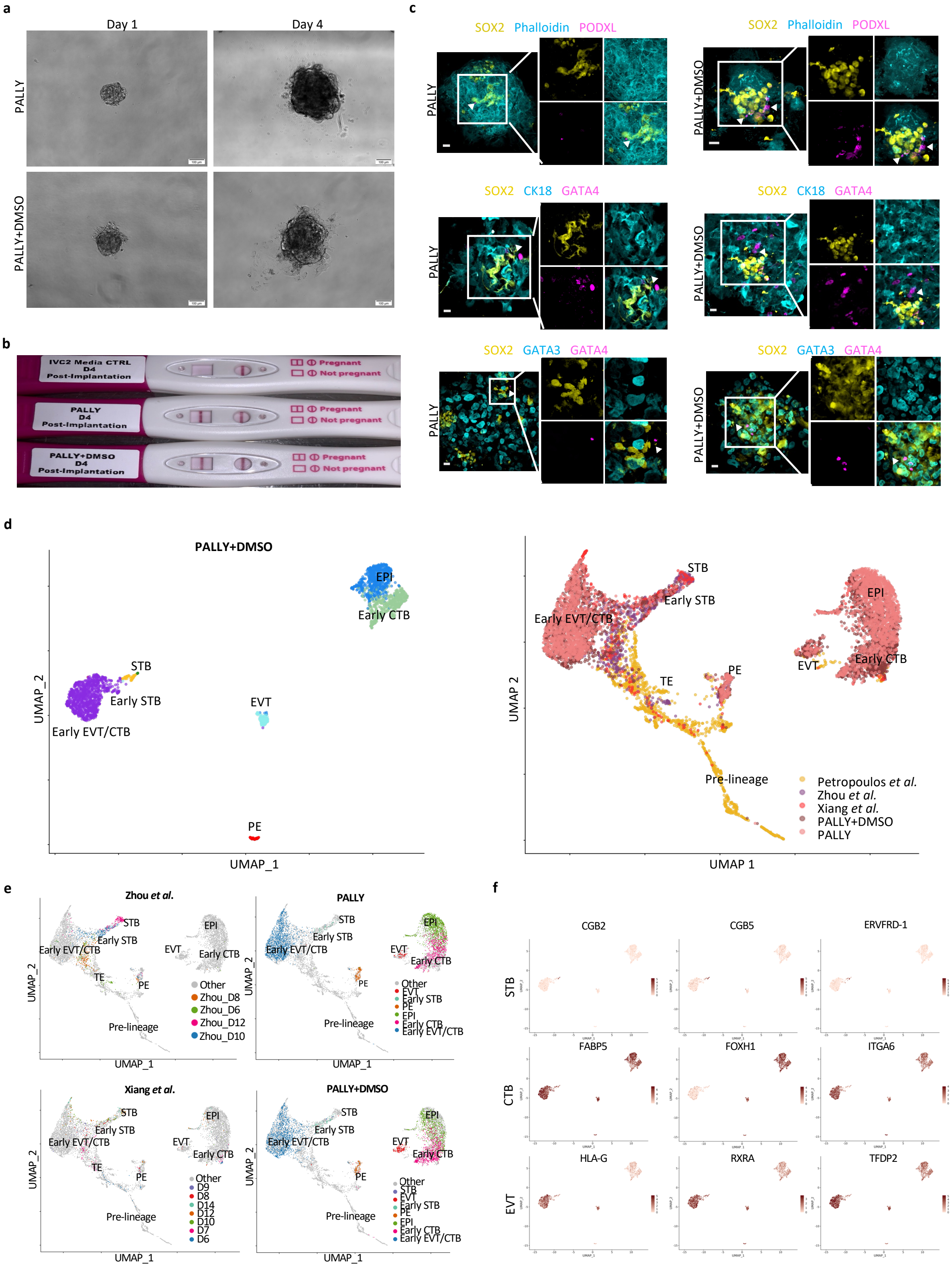
a
Day 1
Day 4
PALLY
PALLY+DMSO
b
IVC2 Media CTRL D4 Post-Implantation
PALLY D4 Post-Implantation
PALLY+DMSO D4 Post-Implantation
Pregnant
Not pregnant
c
SOX2 Phalloidin PODXL
SOX2 CK18 GATA4
SOX2 GATA3 GATA4
PALLY
PALLY+DMSO
d
PALLY+DMSO
EPI
Early CTB
STB
Early STB
Early EVT/CTB
EVT
PE
UMAP_1
UMAP_2
TE
Pre-lineage
Petropoulos et al.
Zhou et al.
Xiang et al.
PALLY+DMSO
PALLY
UMAP 1
UMAP 2
e
Zhou et al.
PALLY
Xiang et al.
PALLY+DMSO
Other
Zhou_D8
Zhou_D6
Zhou_D12
Zhou_D10
D9
D8
D14
D12
D10
D7
D6
f
CGB2
CGB5
ERVFRD-1
FABP5
FOXH1
ITGA6
HLA-G
RXRA
TFDP2
STB
CTB
EVT

Fig. S4 Effect of DMSO in the PALLY-derived peri-implantation blastoids. a Brightfield images show the successful implantation of blastoid in PALLY and PALLY+0.5% DMSO conditions (n = 3). Scale bar, 100 µm**. b** Commercial pregnancy test kit detects stimulated hCGβ under various conditions. **c** Immunofluorescence analysis of SOX2, Phalloidin, and PODXL (**top**); SOX2, CK18, and GATA4 (**middle**); and SOX2, GATA3, and GATA4 (**bottom**) of the attached structures (n = 2). Scale bar, 60 µm. **d** UMAPs of the transcriptome of 3875 single cells of attached PALLY+0.5% DMSO blastoids with cell type annotations (**left**), alongside an integrated dataset projection with published data (**right**). **e** UMAP projections of integrated datasets showing cells from this study and previously published reports. **f** Feature plots of markers of TE subtypes (STB, CTB, and EVT).
