# Supplementary material for: A single small molecule-based human embryo model reveals V-ATPase requirement in mammalian blastocyst cavitation
Source: Cell Res. 2026 Apr 6;36(7):475–98. doi: 10.1038/s41422-026-01239-3 (PMC13287814; doi:10.1038/s41422-026-01239-3)
Supplement: Supplementary file 5 — Supplementary information, Fig. S5 [file 41422_2026_1239_MOESM5_ESM.pdf]

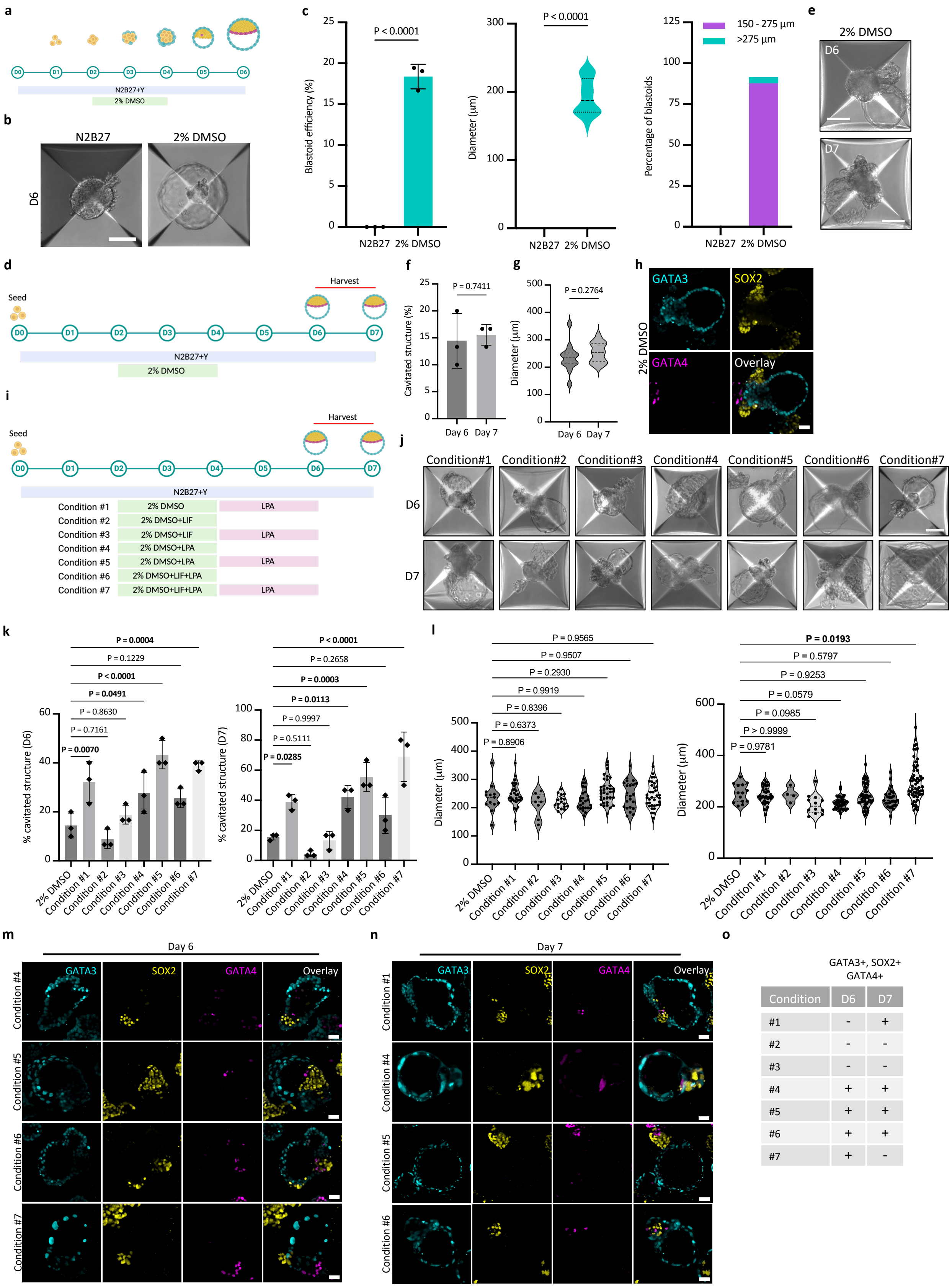

Condition	D6	D7
#1	-	+
#2	-	-
#3	-	-
#4	+	+
#5	+	+
#6	+	+
#7	+	-

Fig. S5 Generation of blastoids using 2% DMSO alone. **a** Schematic representation of the generation of blastocyst-like structures using 2% DMSO. **b** Representative phase-contrast images of DMSO-derived cavitated structures in AggreWell microwells on D6 (n = 3). Scale bar, 100 µm. **c** Percentage of cavitated structures (**left**), size (**middle**), and percentage size distribution (**right**) of the structures. Data are presented as the mean ± standard deviation from three independent experiments. A two-tailed t-test was used, and P values are as indicated. The morphometric definition of human blastocyst-like structures is provided in *Methods*. **d** Schematic representation of the effect of extended culture on human blastocyst-like structure generation. **e** Representative phase-contrast images of DMSO-derived cavitated structures in AggreWell microwells on D6 and D7 (n = 3). Scale bar, 100 µm. **f, g** Comparison of percentage cavitation (**f**) and size (**g**) of the D6 and D7 structures. Data are presented as the mean ± standard deviation from three independent experiments. A two-tailed t-test was used, and P values are as indicated. **h** Immunofluorescence analysis of the TE marker (GATA3; cyan), EPI marker (SOX2; yellow), and PE marker (GATA4; magenta) (n = 3). Scale bar, 50 µm. **i** Schematic representation of the generation of human blastocyst-like structures using various conditions. **j** Representative phase-contrast images of cavitated structures in AggreWell microwells using various treatment conditions and time points as outlined in (**i**) (n = 3). Scale bar, 100 µm. **k** Graphs show the percentage cavitation in different treatment conditions in D6 (**left**) and D7 (**right**) structures. Data are presented as mean ± standard deviation from three independent experiments. One-way ANOVA followed by the Dunnett post hoc test was used, and P values are as indicated. **l** Graphs show the diameter of the cavitated structures in different treatment conditions in D6 (**left**) and D7 (**right**) structures. Data are presented as mean ± standard deviation from three independent experiments. One-way ANOVA followed by the Dunnett post hoc test was used, and P values are as indicated. **m, n** Immunofluorescence analysis of the TE marker (GATA3; cyan), EPI marker (SOX2; yellow), and PE marker (GATA4; magenta) in various treatment conditions at D6 (**m**) and D7 (**n**) (n = 3). Scale bar, 50 µm. **o** Summary table shows the effect of various treatment conditions and time windows on trilineage development. (+) indicates the presence of all three lineages (GATA3+, SOX2+, and GATA4+), and (-) indicates the absence of at least one marker.
