# Supplementary material for: A single small molecule-based human embryo model reveals V-ATPase requirement in mammalian blastocyst cavitation
Source: Cell Res. 2026 Apr 6;36(7):475–98. doi: 10.1038/s41422-026-01239-3 (PMC13287814; doi:10.1038/s41422-026-01239-3)
Supplement: Supplementary file 6 — Supplementary information, Fig. S6 [file 41422_2026_1239_MOESM6_ESM.pdf]

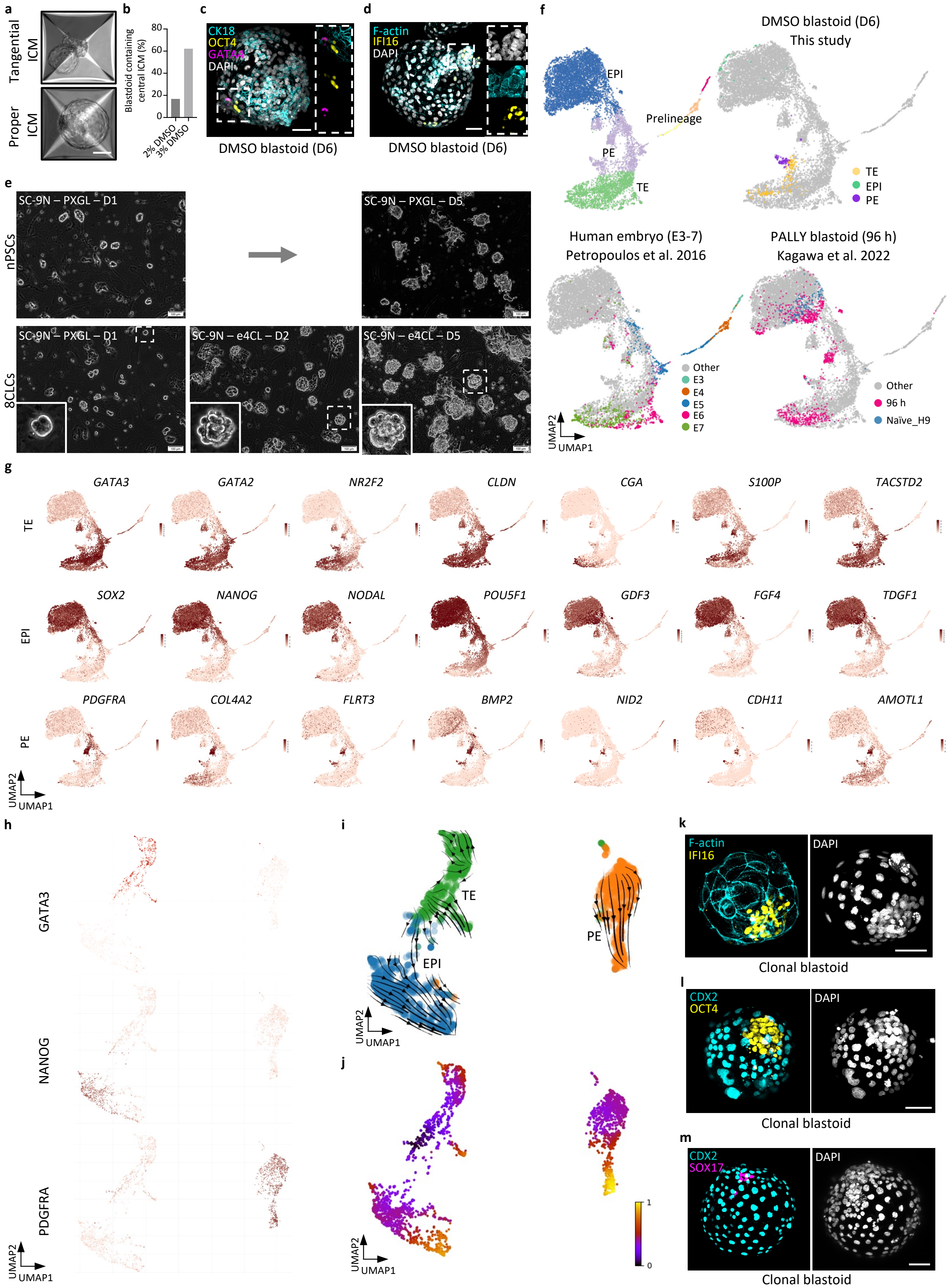
a
Tangential ICM
Proper ICM
b
Blastoid containing central ICM (%)
80
60
40
20
0
2% DMSO
3% DMSO
c
CK18
OCT4
GATA4
DAPI
DMSO blastoid (D6)
d
F-actin
IFI16
DAPI
DMSO blastoid (D6)
e
SC-9N – PXGL – D1
SC-9N – PXGL – D5
nPSCs
SC-9N – PXGL – D1
SC-9N – e4CL – D2
SC-9N – e4CL – D5
8CLCs
f
EPI
Prelineage
PE
TE
DMSO blastoid (D6)
This study
TE
EPI
PE
Human embryo (E3-7)
Petropoulos et al. 2016
Other
E3
E4
E5
E6
E7
PALLY blastoid (96 h)
Kagawa et al. 2022
Other
96 h
Naïve_H9
UMAP2
UMAP1
g
TE
GATA3
GATA2
NR2F2
CLDN
CGA
S100P
TACSTD2
EPI
SOX2
NANOG
NODAL
POU5F1
GDF3
FGF4
TDGF1
PE
PDGFRA
COL4A2
FLRT3
BMP2
NID2
CDH11
AMOTL1
UMAP2
UMAP1
h
GATA3
NANOG
PDGFRA
UMAP2
UMAP1
i
TE
EPI
PE
UMAP2
UMAP1
j
1
0
UMAP2
UMAP1
k
F-actin
IFI16
DAPI
Clonal blastoid
l
CDX2
OCT4
DAPI
Clonal blastoid
m
CDX2
SOX17
DAPI
Clonal blastoid

Fig. S6 Generation of blastoids from 3% DMSO alone. **a** Representative brightfield images of DMSO-derived cavitated structures. Scale bar, 100 μm. **b** Graph shows the percentage of blastoids showing proper ICM. **c** Immunofluorescence images showing the expression of CK18 (cyan), OCT4 (yellow), and GATA4 (magenta) (n = 3). Scale bar, 50 μm. **d** Immunofluorescence images showing the staining of F-actin (cyan) and IFI16 (yellow) (n = 3). Scale bar, 50 μm. **e** Representative brightfield images show the formation of 8CLCs from SC-9N nPSCs (n=2). Scale bar, 100 μm. **f** An independent trial of scRNAseq analysis of day 6 3% DMSO blastoids showing UMAP-based clustering with lineage annotation and integration with published datasets. **g, h** Features plots show the marker gene expression of the TE, EPI, and PE lineages from two different trials. **i** RNA velocity trajectory analysis of day 6 3% DMSO blastoids. **j** Latent time analysis based on RNA velocity dynamics reveals the temporal relationships among individual cells. **k-m** Immunofluorescence images of clonal blastoids showing the expression pattern of F-actin, IFI16, CDX2, OCT4, and SOX17 (n = 3). Scale bar, 50 μm.
