# Supplementary material for: A single small molecule-based human embryo model reveals V-ATPase requirement in mammalian blastocyst cavitation
Source: Cell Res. 2026 Apr 6;36(7):475–98. doi: 10.1038/s41422-026-01239-3 (PMC13287814; doi:10.1038/s41422-026-01239-3)
Supplement: Supplementary file 7 — Supplementary information, Fig. S7 [file 41422_2026_1239_MOESM7_ESM.pdf]

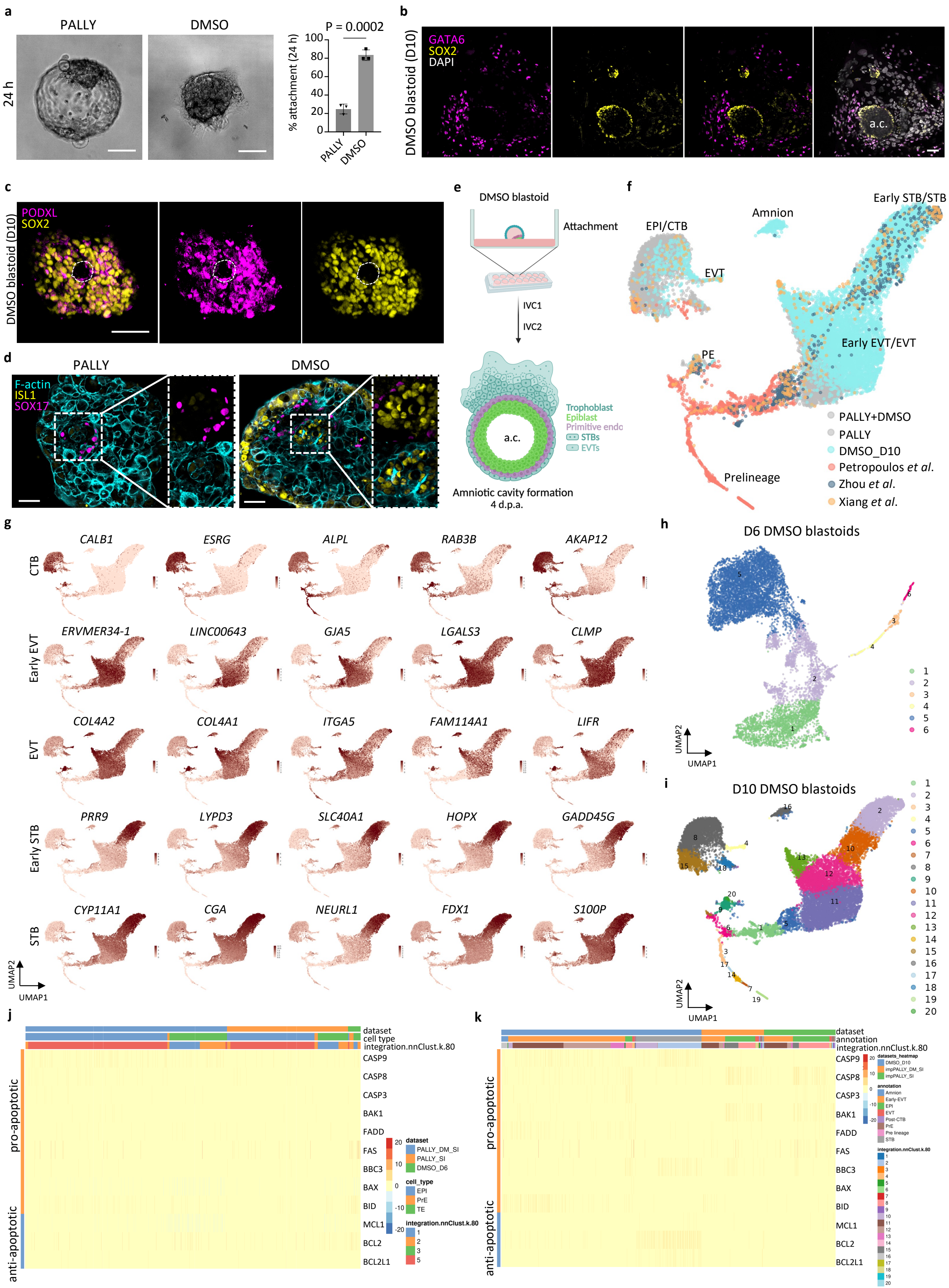
a
PALLY
DMSO
24 h
P = 0.0002
% attachment (24 h)
100
80
60
40
20
0
PALLY
DMSO
b
DMSO blastoid (D10)
GATA6
SOX2
DAPI
a.c.
c
DMSO blastoid (D10)
PODXL
SOX2
d
PALLY
DMSO
F-actin
ISL1
SOX17
e
DMSO blastoid
Attachment
IVC1
IVC2
a.c.
Trophoblast
Epiblast
Primitive endo
STBs
EVTs
Amniotic cavity formation
4 d.p.a.
f
EPI/CTB
Amnion
Early STB/STB
EVT
Early EVT/EVT
PE
Prelineage
PALLY+DMSO
PALLY
DMSO_D10
Petropoulos et al.
Zhou et al.
Xiang et al.
g
CTB
CALB1
ESRG
ALPL
RAB3B
AKAP12
Early EVT
ERVMER34-1
LINC00643
GJA5
LGALS3
CLMP
EVT
COL4A2
COL4A1
ITGA5
FAM114A1
LIFR
Early STB
PRR9
LYPD3
SLC40A1
HOPX
GADD45G
STB
CYP11A1
CGA
NEURL1
FDX1
S100P
UMAP2
UMAP1
h
D6 DMSO blastoids
1
2
3
4
5
6
UMAP2
UMAP1
i
D10 DMSO blastoids
1
2
3
4
5
6
7
8
9
10
11
12
13
14
15
16
17
18
19
20
UMAP2
UMAP1
j
dataset
cell type
integration.nnClust.k.80
pro-apoptotic
anti-apoptotic
CASP9
CASP8
CASP3
BAK1
FADD
FAS
BBC3
BAX
BID
MCL1
BCL2
BCL2L1
20
10
0
-10
-20
dataset
PALLY_DM_SI
PALLY_SI
DMSO_D6
cell_type
EPI
PrE
TE
integration.nnClust.k.80
1
2
3
5
k
dataset
annotation
integration.nnClust.k.80
pro-apoptotic
anti-apoptotic
CASP9
CASP8
CASP3
BAK1
FADD
FAS
BBC3
BAX
BID
MCL1
BCL2
BCL2L1
datasets_heatmap
DMSO_D10
impPALLY_DM_SI
impPALLY_SI
annotation
Amnion
Early-EVT
EPI
EVT
Post-CTB
PrE
Pre lineage
STB
integration.nnClust.k.80

Fig. S7 DMSO blastoids model D10 embryogenesis. a Representative brightfield images showing attached PALLY and DMSO blastoids after 24 h (n = 3). Scale bar, 100 µm (**left**). The graph shows the quantification of percentage attached blastoids between PALLY and DMSO 24 h after attachment (n = 3). Student t-test was used. P value is as indicated (**right)**. **b** Immunofluorescence images showing the expression of SOX2 (yellow) and GATA6 (magenta) of attached 3% DMSO blastoid (n =3). Scale bar, 50 µm. **c** Immunofluorescence images showing the expression of SOX2 (yellow) and PODXL (magenta) of attached 3% DMSO blastoid (n =3). Scale bar, 50 µm. **d** Immunofluorescence images showing the staining of F-actin (cyan), ISL1 (yellow), and SOX17 (magenta) of attached PALLY and DMSO blastoid (n =3). Scale bar, 50 µm. **e** Schematic representation of the *in vitro* attachment assay. **f** UMAP projections of integrated datasets showing cells from this study and previously published reports. **g** Feature plots of markers of TE subtypes (Early EVT/EVT, Early STB/STB, and CTB). **h**, **i** UMAPs show the cell clusters of the integrated datasets of pre- (**h**) and post-implantation (**i**) datasets. **j, k** Heatmaps show the expression levels of pro- and anti-apoptotic genes from pre- (**j**) and post-implantation (**k**) datasets.
