# Supplementary material for: A single small molecule-based human embryo model reveals V-ATPase requirement in mammalian blastocyst cavitation
Source: Cell Res. 2026 Apr 6;36(7):475–98. doi: 10.1038/s41422-026-01239-3 (PMC13287814; doi:10.1038/s41422-026-01239-3)
Supplement: Supplementary file 8 — Supplementary information, Fig. S8 [file 41422_2026_1239_MOESM8_ESM.pdf]

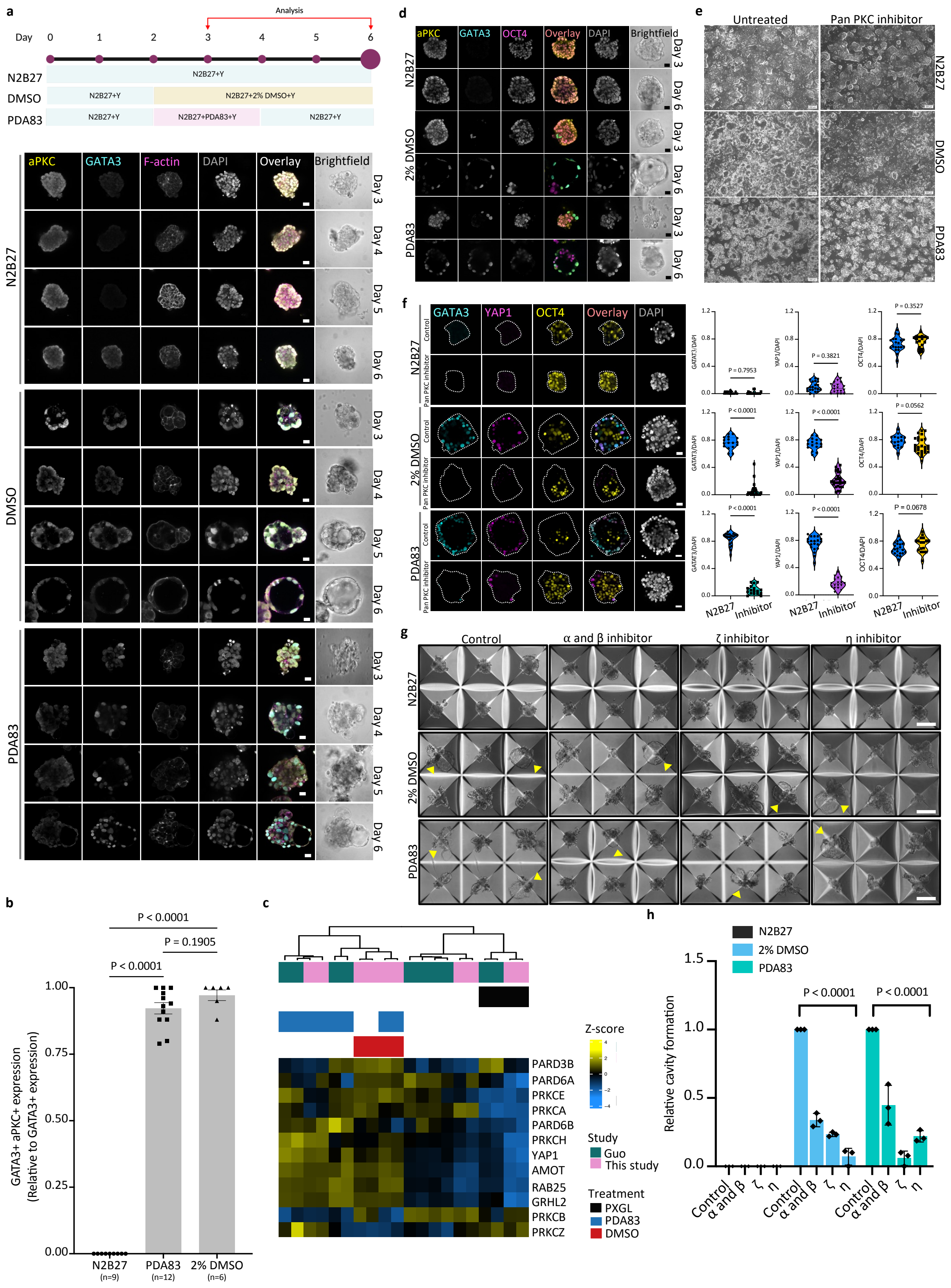
a
Analysis
Day 0 1 2 3 4 5 6
N2B27
N2B27+Y
DMSO
N2B27+Y
N2B27+2% DMSO+Y
PDA83
N2B27+Y
N2B27+PDA83+Y
N2B27+Y
aPKC
GATA3
F-actin
DAPI
Overlay
Brightfield
Day 3
Day 4
Day 5
Day 6
N2B27
DMSO
PDA83
b
P < 0.0001
P = 0.1905
P < 0.0001
GATA3+ aPKC+ expression (Relative to GATA3+ expression)
1.00
0.75
0.50
0.25
0.00
N2B27 (n=9)
PDA83 (n=12)
2% DMSO (n=6)
c
PARD3B
PARD6A
PRKCE
PRKCA
PARD6B
PRKCH
YAP1
AMOT
RAB25
GRHL2
PRKCB
PRKCZ
Z-score
Study
Guo
This study
Treatment
PXGL
PDA83
DMSO
d
aPKC
GATA3
OCT4
Overlay
DAPI
Brightfield
N2B27
2% DMSO
PDA83
e
Untreated
Pan PKC inhibitor
N2B27
DMSO
PDA83
f
GATA3
YAP1
OCT4
Overlay
DAPI
Control
Pan PKC inhibitor
N2B27
2% DMSO
PDA83
P = 0.7953
P = 0.3821
P = 0.3527
P < 0.0001
P < 0.0001
P = 0.0562
P < 0.0001
P < 0.0001
P = 0.0678
GATA3/DAPI
YAP1/DAPI
OCT4/DAPI
N2B27
Inhibitor
g
Control
α and β inhibitor
ζ inhibitor
η inhibitor
N2B27
2% DMSO
PDA83
h
N2B27
2% DMSO
PDA83
Relative cavity formation
1.5
1.0
0.5
0.0
P < 0.0001
P < 0.0001
Control
α and β
ζ
η

Fig. S8 DMSO is a downstream regulator of the aPKC pathway and mediates cavity formation. a Immunofluorescence analysis shows the colocalization pattern of aPKC (yellow), GATA3 (cyan), and F-actin (magenta) in N2B27, DMSO, and PDA83 conditions. Scale bar, 25 μm. Schematic representation of the treatment process is depicted in respective conditions. **b** Quantitative analysis of day 6 structures from (**a**). Data are presented as mean ± standard deviation. The number of structures analyzed is as indicated. One-way ANOVA followed by Tukey's post hoc test was used, and P values are as indicated. **c** Heatmap shows the enrichment of aPKC cell polarity genes. **d** Representative immunofluorescence images showing the expression of aPKC (yellow), GATA3 (cyan), and OCT4 (magenta) of D3 and D6 structures in N2B27, 2% DMSO, and PDA83 conditions. Scale bar, 25 μm. **e** Representative brightfield images of day 4 samples in N2B27, DMSO, and PDA83 conditions with and without treatment with PKC inhibitor (n = 3). **f** Representative immunofluorescence images of D6 structures treated with pan PKC inhibitor showing the expression of GATA3 (cyan), YAP1 (magenta), and OCT4 (yellow) in N2B27, DMSO, and PDA83 conditions. Scale bar, 25 μm. Quantification of GATA3, YAP1, and OCT4 normalized fluorescence intensity of the structures in the above conditions is provided in the respective panel. Two-tailed t-test was used, and P values are as indicated. **g** Representative brightfield images of day 6 structures in N2B27, DMSO, and PDA83 conditions (n = 3). The yellow arrow indicates properly formed cavitated structures. Scale bar, 200 μm. **h** Relative cavitation efficiency after treatments with different inhibitors of PKC isoforms in N2B27, DMSO, and PDA83 conditions. Data are presented as mean ± standard deviation from three independent experiments. Two-way ANOVA followed by Tukey's post hoc test was used, and P values are as indicated.
