# Supplementary material for: A single small molecule-based human embryo model reveals V-ATPase requirement in mammalian blastocyst cavitation
Source: Cell Res. 2026 Apr 6;36(7):475–98. doi: 10.1038/s41422-026-01239-3 (PMC13287814; doi:10.1038/s41422-026-01239-3)
Supplement: Supplementary file 9 — Supplementary information, Fig. S9 [file 41422_2026_1239_MOESM9_ESM.pdf]

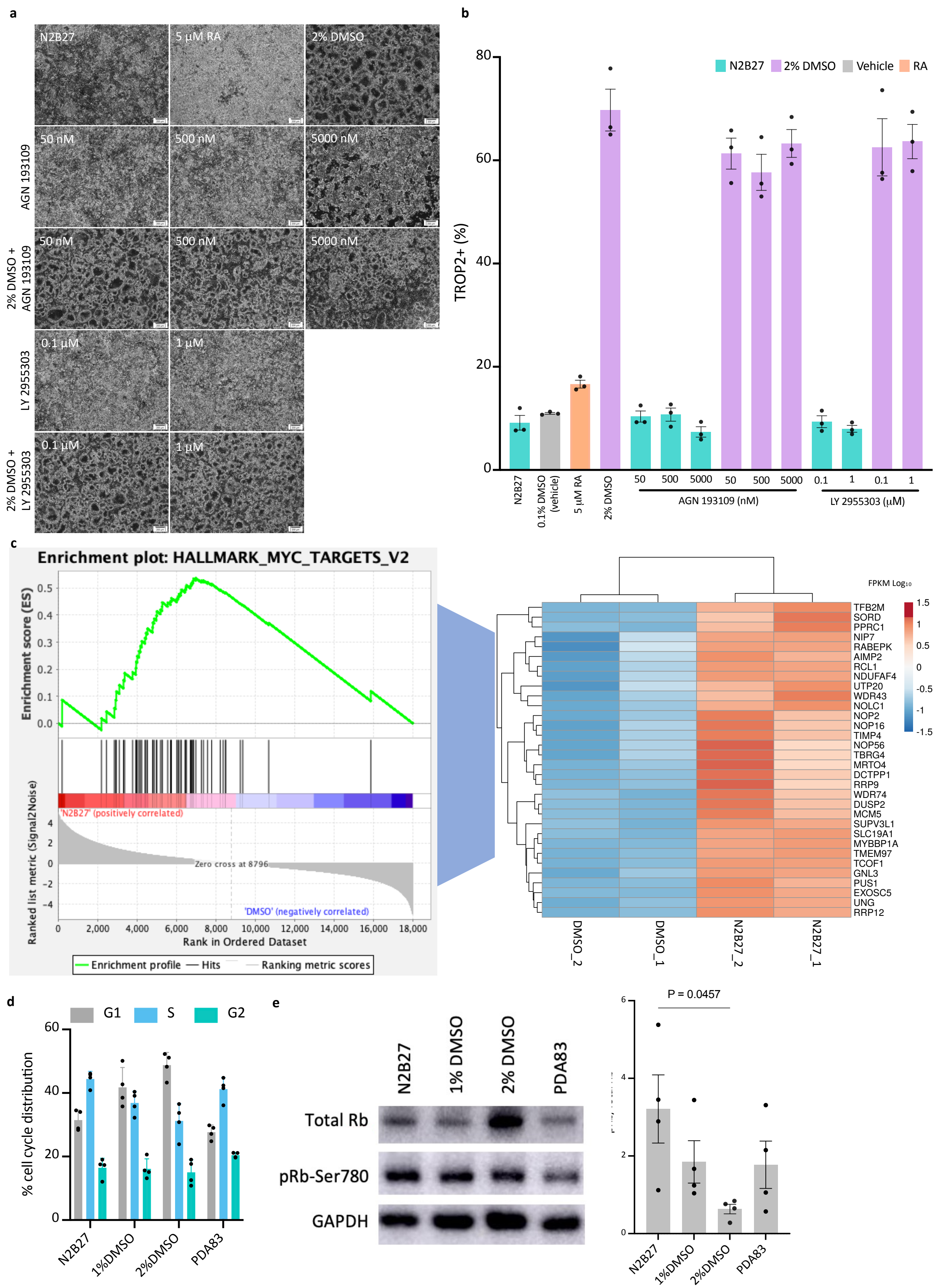
a
N2B27
5 µM RA
2% DMSO
AGN 193109
50 nM
500 nM
5000 nM
2% DMSO + AGN 193109
LY 2955303
0.1 µM
1 µM
2% DMSO + LY 2955303
b
N2B27
2% DMSO
Vehicle
RA
TROP2+ (%)
0
20
40
60
80
0.1% DMSO (vehicle)
5 µM RA
50
500
5000
0.1
1
AGN 193109 (nM)
LY 2955303 (µM)
c
Enrichment plot: HALLMARK_MYC_TARGETS_V2
Enrichment score (ES)
Ranked list metric (Signal2Noise)
'N2B27' (positively correlated)
Zero cross at 8796
'DMSO' (negatively correlated)
Rank in Ordered Dataset
Enrichment profile
Hits
Ranking metric scores
FPKM Log10
TFB2M
SORD
PPRC1
NIP7
RABEPK
AIMP2
RCL1
NDUFAF4
UTP20
WDR43
NOLC1
NOP2
NOP16
TIMP4
NOP56
TBRG4
MRTO4
DCTPP1
RRP9
WDR74
DUSP2
MCM5
SUPV3L1
SLC19A1
MYBBP1A
TMEM97
TCOF1
GNL3
PUS1
EXOSC5
UNG
RRP12
DMSO_2
DMSO_1
N2B27_2
N2B27_1
d
G1
S
G2
% cell cycle distribution
N2B27
1%DMSO
2%DMSO
PDA83
e
N2B27
1% DMSO
2% DMSO
PDA83
Total Rb
pRb-Ser780
GAPDH
P = 0.0457

Fig. S9 Effects of RA and cell cycle in DMSO-derived blastoids. a Representative brightfield images of day 4 samples in N2B27, DMSO, RA (5 μM), AGN193109 (a pan-RAR antagonist used at 50, 500, and 5000 nM), and LY2955303 (a selective RARγ antagonist used at 0.1 and 1 μM) conditions (n = 3). Scale bar, 200 μm. **b** FACS analysis of samples from (**a**). Data are presented as mean ± standard error of the mean (n=3). **c** Gene Set Enrichment plot and gene expression heatmap of genes related to proliferation in DMSO and N2B27 conditions. **d** Bar graph shows the percentage distribution of cell cycle phases in N2B27, 1%/2% DMSO, and PDA83 conditions. Data are presented as the mean ± standard deviation from four independent experiments. **e** Western blot analysis shows the expression of the mentioned proteins (**left**), and quantification shows the normalized pRb-Ser780 expression (**right**). GAPDH was used as a loading control. Data are presented as the mean ± standard deviation (n = 4). One-way ANOVA followed by Tukey's post hoc test was used, and P value is as indicated.
