# Supplementary material for: A single small molecule-based human embryo model reveals V-ATPase requirement in mammalian blastocyst cavitation
Source: Cell Res. 2026 Apr 6;36(7):475–98. doi: 10.1038/s41422-026-01239-3 (PMC13287814; doi:10.1038/s41422-026-01239-3)
Supplement: Supplementary file 10 — Supplementary information, Fig. S10 [file 41422_2026_1239_MOESM10_ESM.pdf]

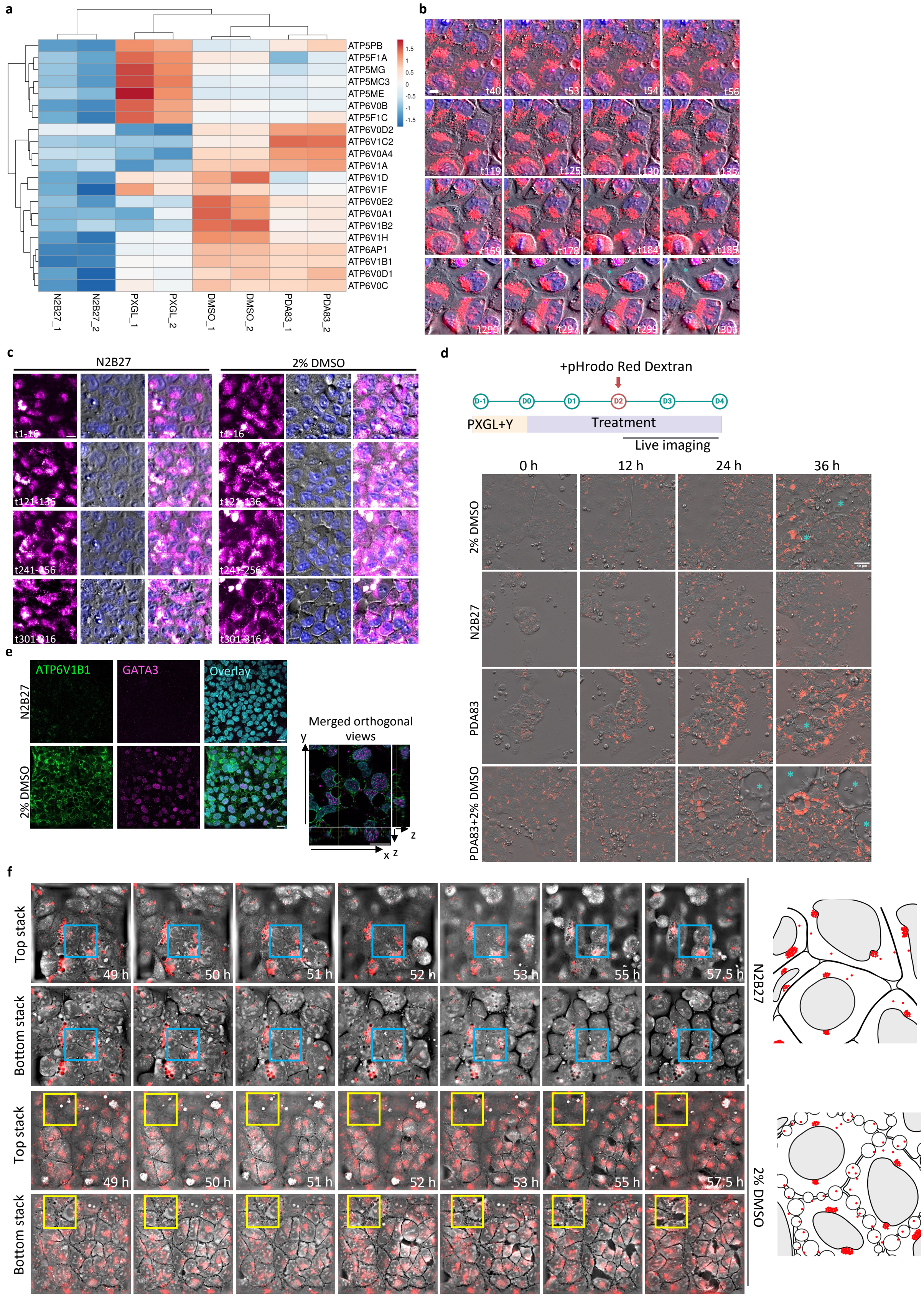
a
ATP5PB
ATP5F1A
ATP5MG
ATP5MC3
ATP5ME
ATP6V0B
ATP5F1C
ATP6V0D2
ATP6V1C2
ATP6V0A4
ATP6V1A
ATP6V1D
ATP6V1F
ATP6V0E2
ATP6V0A1
ATP6V1B2
ATP6V1H
ATP6AP1
ATP6V1B1
ATP6V0D1
ATP6V0C
1.5
1
0.5
0
-0.5
-1
-1.5
N2B27_1
N2B27_2
PXGL_1
PXGL_2
DMSO_1
DMSO_2
PDA83_1
PDA83_2
b
t40
t53
t54
t56
t119
t125
t130
t135
t169
t178
t184
t185
t290
t297
t299
t300
c
N2B27
2% DMSO
t1-16
t121-136
t241-256
t301-316
d
+pHrodo Red Dextran
D-1
D0
D1
D2
D3
D4
PXGL+Y
Treatment
Live imaging
0 h
12 h
24 h
36 h
2% DMSO
N2B27
PDA83
PDA83+2% DMSO
e
ATP6V1B1
GATA3
Overlay
N2B27
2% DMSO
Merged orthogonal views
y
z
x z
f
Top stack
Bottom stack
49 h
50 h
51 h
52 h
53 h
55 h
57.5 h
N2B27
2% DMSO

Fig. S10 DMSO induces TE cavitation through the lysosome pathway. **a** Heatmap showing expression of ATP5 and ATP6 family genes in cells under DMSO, PDA83, and N2B27 differentiation conditions, as well as in PXGL-maintained nPSCs (log_{10} transformed gene expression values). Biological replicates, n=2. **b** Merged live cell fluorescence and oblique illumination microscopy images of a single timepoint related to Fig. 4d show the morphology change and lysosome dynamics in DMSO condition (n = 2). Lysotracker (red) and Hoechst 33342 (blue) were added on day 2, and images were taken every 2 minutes. Time points indicate the number of minutes since imaging started. Cyan asterisk: TE cyst. Scale bar, 10 µm. **c** Live cell fluorescence and oblique illumination microscopy images of 2% DMSO differentiation culture as shown in Fig. 4d. Lysotracker (magenta) and Hoechst 33342 (blue) were added at day 2 (n = 2). Time points indicate the number of minutes since imaging started. Cyan asterisk: TE cyst. Scale bar, 10 µm **d** Live cell fluorescence, and oblique illumination microscopy images from different treatment conditions (n = 3). pHrodo red dextran (red) was added on day 2. Time points indicate the number of hours since imaging started. Cyan asterisk: TE cyst. Scale bar, 40 µm. **e** Immunofluorescence analysis shows the expression of ATP6V1B1 (green) and GATA3 (purple) under N2B27 and DMSO conditions at day 4 (left). The orthogonal view demonstrates higher magnification images to show the detailed morphology (right). Scale bar, 20 µm. **f** Snapshots of live cell imaging reveal the formation of microlumen in N2B27 and DMSO conditions. The blue and yellow areas were analyzed to quantify microlumen formation. Refer to Fig. 4h for quantification. Scale bar, 20 µm. Cartoons on the right side depict the accumulation of microlumens.
