# Supplementary material for: A single small molecule-based human embryo model reveals V-ATPase requirement in mammalian blastocyst cavitation
Source: Cell Res. 2026 Apr 6;36(7):475–98. doi: 10.1038/s41422-026-01239-3 (PMC13287814; doi:10.1038/s41422-026-01239-3)
Supplement: Supplementary file 11 — Supplementary information, Fig. S11 [file 41422_2026_1239_MOESM11_ESM.pdf]

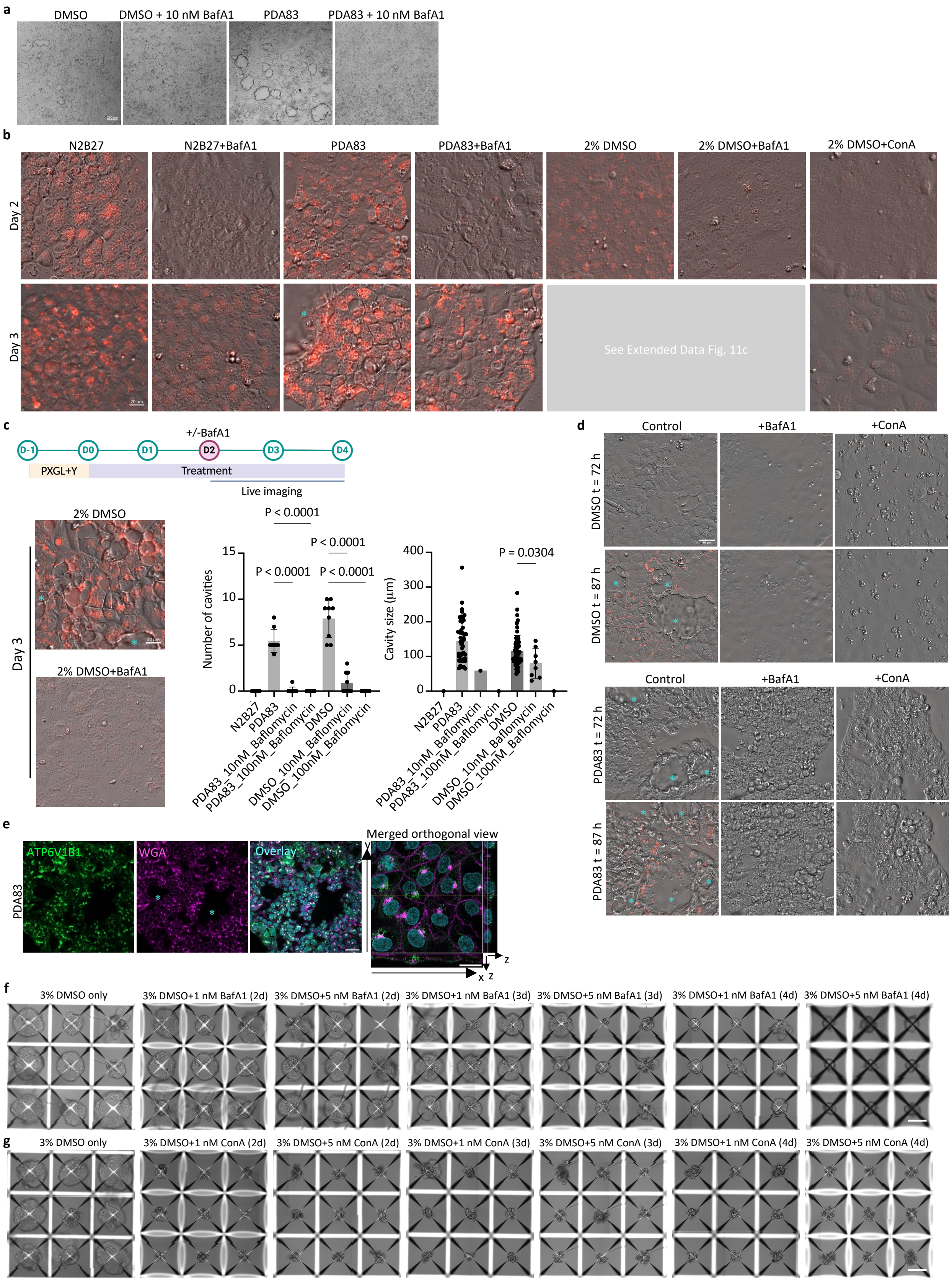
a
DMSO
DMSO + 10 nM BafA1
PDA83
PDA83 + 10 nM BafA1
b
N2B27
N2B27+BafA1
PDA83
PDA83+BafA1
2% DMSO
2% DMSO+BafA1
2% DMSO+ConA
Day 2
Day 3
See Extended Data Fig. 11c
c
+/-BafA1
D-1
D0
D1
D2
D3
D4
PXGL+Y
Treatment
Live imaging
2% DMSO
2% DMSO+BafA1
Day 3
Number of cavities
Cavity size (μm)
P < 0.0001
P < 0.0001
P < 0.0001
P < 0.0001
P = 0.0304
N2B27
PDA83
PDA83_10nM_Baflomycin
PDA83_100nM_Baflomycin
DMSO
DMSO_10nM_Baflomycin
DMSO_100nM_Baflomycin
d
Control
+BafA1
+ConA
DMSO t = 72 h
DMSO t = 87 h
PDA83 t = 72 h
PDA83 t = 87 h
e
ATP6V1B1
WGA
Overlay
Merged orthogonal view
PDA83
f
3% DMSO only
3% DMSO+1 nM BafA1 (2d)
3% DMSO+5 nM BafA1 (2d)
3% DMSO+1 nM BafA1 (3d)
3% DMSO+5 nM BafA1 (3d)
3% DMSO+1 nM BafA1 (4d)
3% DMSO+5 nM BafA1 (4d)
g
3% DMSO only
3% DMSO+1 nM ConA (2d)
3% DMSO+5 nM ConA (2d)
3% DMSO+1 nM ConA (3d)
3% DMSO+5 nM ConA (3d)
3% DMSO+1 nM ConA (4d)
3% DMSO+5 nM ConA (4d)

Fig. S11 Effect of V-ATPase inhibitors on TE cyst formation and blastoid cavitation. a Tiled brightfield images of day 4 TE differentiation with DMSO and PDA83 treated with and without 10 nM BafA1 from day 0 to day 4. Scale bar, 200 µm. Biological replicate n=3. **b** Live cell images taken on day 2 or 3 after adding Lysotracker (red). Cells were treated with or without BafA1 (10 nM) or ConA (10 nM) under different conditions (n = 2). Cyan asterisk: TE cyst. Scale bar, 20 µm. **c** Day 3 live-cell oblique illumination bright field images overlayed with fluorescence Lysotracker (red) signals in 2% DMSO differentiation culture with or without BafA1 (10 nM) (n = 2). Cyan asterisk: TE cyst. Scale bar, 20 µm. The bar graphs show the quantification of the number of cavities formed and their sizes under various conditions (n=3). Data are presented as the mean ± standard deviation from three independent experiments. One-way ANOVA followed by Tukey's post hoc test was used (top graph), and the Student t-test was used in the bottom graph. P values are as indicated. **d** Live cell images were taken on day 3 of the differentiation experiment (t=72 h, after adding pHrodo Red dextran) and 15 h later (t=87 h). Cells were treated with or without BafA1 (10 nM) or ConA (10 nM) under different conditions (n = 2). Cyan asterisk: TE cyst. Scale bar, 20 µm. **e** Immunofluorescence analysis shows the expression of ATP6V1B1 (green) and wheat germ agglutinin (magenta) under PDA83 conditions on day 3 of the differentiation experiment (**left**) (n = 3). Scale bar, 50 µm. The orthogonal view demonstrates higher magnification images to show the detailed morphology (**right**). Cyan asterisk: TE cyst. Scale bar, 20 µm. **f, g** Representative brightfield images of day 5 structures showing the effect of BafA1 (**f**) and ConA (**g**) on 3% DMSO-derived blastoid formation assay. Scale bar, 200 µm.
