# Supplementary material for: A single small molecule-based human embryo model reveals V-ATPase requirement in mammalian blastocyst cavitation
Source: Cell Res. 2026 Apr 6;36(7):475–98. doi: 10.1038/s41422-026-01239-3 (PMC13287814; doi:10.1038/s41422-026-01239-3)
Supplement: Supplementary file 12 — Supplementary information, Fig. S12 [file 41422_2026_1239_MOESM12_ESM.pdf]

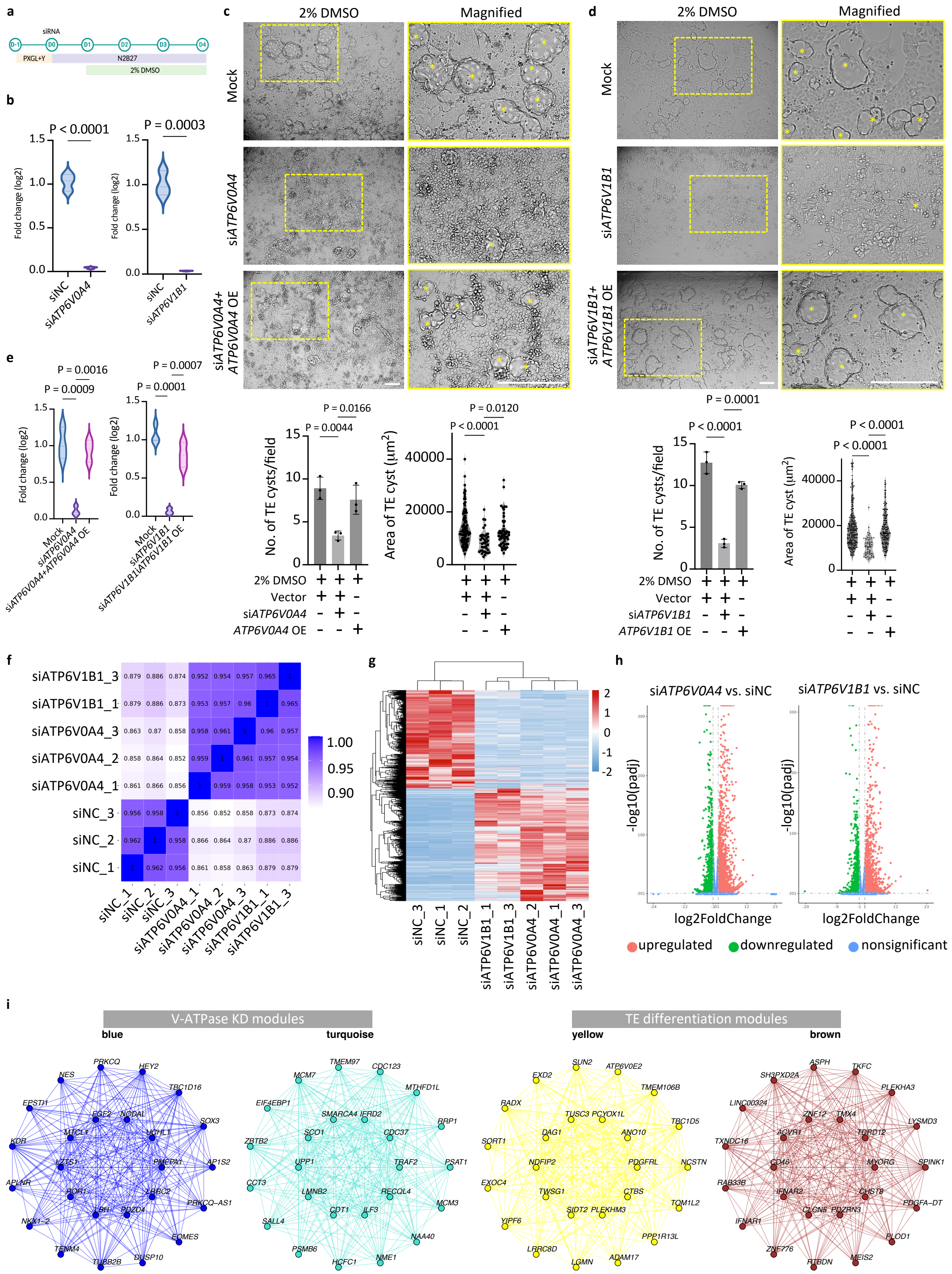
a
siRNA
D-1
D0
D1
D2
D3
D4
PXGL+Y
N2B27
2% DMSO
b
P < 0.0001
P = 0.0003
Fold change (log2)
siNC
siATP6V0A4
siATP6V1B1
c
2% DMSO
Magnified
Mock
siATP6V0A4
siATP6V0A4+ ATP6V0A4 OE
d
siATP6V1B1
siATP6V1B1+ ATP6V1B1 OE
e
P = 0.0016
P = 0.0009
P = 0.0007
P = 0.0001
Mock
siATP6V0A4+ATP6V0A4 OE
siATP6V1B1+ATP6V1B1 OE
P = 0.0166
P = 0.0044
P = 0.0120
P < 0.0001
No. of TE cysts/field
Area of TE cyst (μm²)
Vector
ATP6V0A4 OE
ATP6V1B1 OE
f
siATP6V1B1_3
siATP6V1B1_1
siATP6V0A4_3
siATP6V0A4_2
siATP6V0A4_1
siNC_3
siNC_2
siNC_1
g
h
siATP6V0A4 vs. siNC
siATP6V1B1 vs. siNC
-log10(padj)
log2FoldChange
upregulated
downregulated
nonsignificant
i
V-ATPase KD modules
TE differentiation modules
blue
turquoise
yellow
brown

Fig. S12 Effect of V-ATPase knockdown on TE cyst formation. a Schematic illustration of the siRNA transfection strategy used in the SC-9N cell line. **b** Graphs show the efficiency of siRNA targeting *ATP6V0A4* and *ATP6V1B1*. Data are presented as the mean ± standard deviation from three independent experiments. Student's t-test was used. P value is as indicated. **c, d** Representative brightfield images show the effects of *ATP6V0A4* and *ATP6V1B1* knockdown, as well as the corresponding rescue effects, on TE cyst formation. Scale bars, 100 µm. Bar graphs show the quantification of the number of TE cysts formed and their area. Data are presented as the mean and standard deviation from three independent experiments. One-way ANOVA followed by the Dunnett post hoc test was used. P values are as indicated. **e** Graphs show the efficiency of siRNA targeting *ATP6V0A4* and *ATP6V1B1* and the corresponding rescue effects. Data are presented as the mean ± standard deviation from three independent experiments. One-way ANOVA followed by the Dunnett post hoc test was used. P values are as indicated. **f** Heatmap of Pearson correlation coefficients showing the similarity between RNA-seq datasets across the indicated groups. **g** Heatmap displaying gene expression profiles for the siNC, si*ATP6V0A4*, and si*ATP6V1B1* groups. **h** Volcano plots showing differentially expressed genes in si*ATP6V0A4* vs. siNC (**left**) and si*ATP6V1B1* vs. siNC (**right**) groups. **i** WGCNA network modules associated with knockdown (blue and turquoise) and TE differentiation (yellow and brown).
