# Supplementary material for: A single small molecule-based human embryo model reveals V-ATPase requirement in mammalian blastocyst cavitation
Source: Cell Res. 2026 Apr 6;36(7):475–98. doi: 10.1038/s41422-026-01239-3 (PMC13287814; doi:10.1038/s41422-026-01239-3)
Supplement: Supplementary file 13 — Supplementary information, Fig. S13 [file 41422_2026_1239_MOESM13_ESM.pdf]

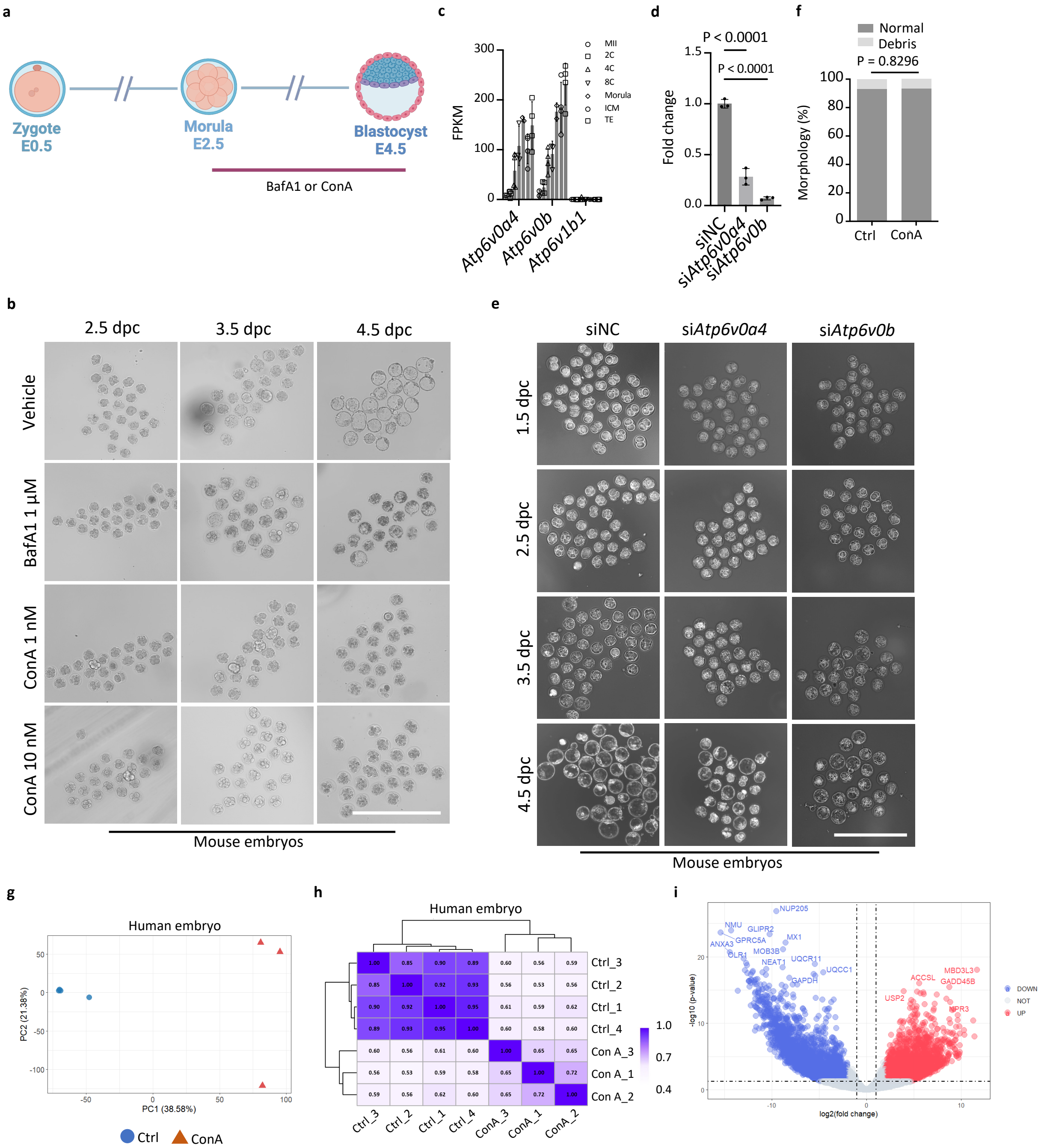
a
Zygote
E0.5
Morula
E2.5
Blastocyst
E4.5
BafA1 or ConA
c
FPKM
300
200
100
0
MII
2C
4C
8C
Morula
ICM
TE
Atp6v0a4
Atp6v0b
Atp6v1b1
d
P < 0.0001
P < 0.0001
Fold change
1.5
1.0
0.5
0.0
siNC
siAtp6v0a4
siAtp6v0b
f
Normal
Debris
P = 0.8296
Morphology (%)
100
80
60
40
20
0
Ctrl
ConA
b
2.5 dpc
3.5 dpc
4.5 dpc
Vehicle
BafA1 1 µM
ConA 1 nM
ConA 10 nM
Mouse embryos
e
siNC
siAtp6v0a4
siAtp6v0b
1.5 dpc
2.5 dpc
3.5 dpc
4.5 dpc
Mouse embryos
g
Human embryo
PC2 (21.38%)
PC1 (38.58%)
Ctrl
ConA
h
Human embryo
Ctrl_3
Ctrl_2
Ctrl_1
Ctrl_4
Con A_3
Con A_1
Con A_2
1.0
0.7
0.4
i
NUP205
NMU
GLIPR2
ANXA3
GPRC5A
MX1
OLR1
MOB3B
NEAT1
UQCR11
UQCC1
GAPDH
ACCSL
MBD3L3
GADD45B
USP2
NPR3
-log10 (p-value)
log2(fold change)
DOWN
NOT
UP

Fig. S13 Ablation of V-ATPase function impairs cavitation in mouse and human embryos. a Schematic showing the experimental design for lysosomal inhibitor treatment in mouse embryos. **b** Brightfield images illustrating the effect of BafA1 and ConA at various concentrations on mouse embryo cavitation. Scale bar, 100 µm**. c** Expression profiles of V-ATPase subunit genes across different developmental stages of mouse embryos using data from a published study[59]. **d** Graph shows the knockdown efficiency of si*Atp6v0a4* and si*Atp6v0b*. Data are presented as the mean and standard deviation from three independent experiments. One-way ANOVA followed by the Dunnett post hoc test was used. P values are as indicated. **e** Brightfield images show the effect of siRNA-mediated knockdown of *Atp6v0a4* and *Atp6v0b* in mouse embryo cavitation. Scale bar, 100 µm**. f** Graph shows the percentage of cellular debris indicating cell death in Ctrl and ConA-treated human embryos. Paired t-test was used. P value is as indicated. **g** PCA plot of Smart-seq2 analysis of Ctrl and ConA-treated human embryos. **h** Heatmap of Pearson correlation coefficients of the transcriptomic profiles of Ctrl and ConA-treated human embryos. **i** Volcano plots showing differentially expressed genes in ConA treatment vs Ctrl datasets.
