# Supplementary material for: A single small molecule-based human embryo model reveals V-ATPase requirement in mammalian blastocyst cavitation
Source: Cell Res. 2026 Apr 6;36(7):475–98. doi: 10.1038/s41422-026-01239-3 (PMC13287814; doi:10.1038/s41422-026-01239-3)
Supplement: Supplementary file 20 — Supplementary information, Video legends [file 41422_2026_1239_MOESM20_ESM.pdf]

Supplementary video legends

Videos S1 and S2: Live cell image analysis shows the formation of blastoids from nPSCs under PALLY (Video S1) and PALLY+0.5% DMSO (Video S2) conditions.

Video S3: Live cell image analysis shows the cyst formation and lysosome dynamics in DMSO treatment starting at day 3. The timestamp represents the time after adding Lysotracker (red) and Hoechst 33342 (blue). Scale bar, 20 μm.

Videos S4 and S5: RI-based 3D holotomography coupled with fluorescence live-cell imaging shows microlumen formation under N2B27 (Video S4) and DMSO (Video S5) conditions. The timestamp represents the treatment duration. Scale bar, 20 μm.

Video S6: Comparison of apical (**left**) and basolateral (**right**) regions shows the formation of microlumens and cysts under DMSO condition. The timestamp represents the treatment duration. Scale bar, 20 μm.
