# Supplementary material for: A single small molecule-based human embryo model reveals V-ATPase requirement in mammalian blastocyst cavitation
Source: Cell Res. 2026 Apr 6;36(7):475–98. doi: 10.1038/s41422-026-01239-3 (PMC13287814; doi:10.1038/s41422-026-01239-3)
Supplement: Supplementary file 21 — Supplementary information, Table S1 [file 41422_2026_1239_MOESM21_ESM.pdf]

Table S1. Antibodies used in this study.

Antibody	Manufacturer	Catalog number
**Primary antibodies**		
CK7	Abcam	ab181598
CK18	Santa Cruz	sc-31700
OCT3/4	Santa Cruz	sc-9081
NANOG	Abcam	ab77095
NANOG	R&D Systems	AF2729
CDX2	Biogenex	AM392-5M
YAP1	Abcam	ab205270
GATA4	Cell Signaling Technology	36966S
PODXL	Cell Signaling Technology	55601S
SOX2	Cell Signaling Technology	4900S
SOX2	R&D Systems	AF2018
HLA-G	Cell Signaling Technology	79769S
Syncytin1	Bioss	BS-2962R
AQP3	Invitrogen	PA5-77840
GATA3	R&D Systems	MAB6300
GATA3	Cell Signaling Technology	5852S
PKCzeta	Abcam	ab59364
Rb (4H1)	Cell Signaling Technology	14731S
Phospho-Rb (Ser780)	Cell Signaling Technology	3590S
ATP6V1B1	Invitrogen	PA5-56878
IFI16	Abcam	ab169788
F-actin (FITC-labeled)	Sigma-Aldrich	P5282
hCGβ	Invitrogen	MA5-18033
GATA6	Cell Signaling Technology	5851S
ISL1	R&D Systems	AF1837
SOX17	R&D Systems	MAB1924
GAPDH	Invitrogen	MA5-15738
**FACS antibodies**		
TROP2	Invitrogen	53-6024-82
CD90	Invitrogen	17-0909-42
SUSD2	BioLegend	327401
**Secondary antibodies**		
Donkey anti-Mouse IgG (H+L) Alexa Fluor 488	Invitrogen	A-21202

Donkey anti-Rabbit IgG (H+L) Alexa Fluor 488	Invitrogen	A-21206
Donkey anti-Goat IgG (H+L) Alexa Fluor 488	Invitrogen	A-11055
Donkey anti-Mouse IgG (H+L) Alexa Fluor 568	Invitrogen	A-10037
Donkey anti-Rabbit IgG (H+L) Alexa Fluor 568	Invitrogen	A-10042
Donkey anti-Goat IgG (H+L) Alexa Fluor 568	Invitrogen	A-11057
Donkey anti-Mouse IgG (H+L) Alexa Fluor 594	Invitrogen	R-37115
Donkey anti-Rabbit IgG (H+L) Alexa Fluor 594	Invitrogen	R-37119
Donkey anti-Goat IgG (H+L) Alexa Fluor 594	Invitrogen	A-11058
Donkey anti-Mouse IgG (H+L) Alexa Fluor 647	Invitrogen	A-31571
Donkey anti-Rabbit IgG (H+L) Alexa Fluor 647	Invitrogen	A-31573
Donkey anti-Goat IgG (H+L) Alexa Fluor 647	Invitrogen	A-21447
Anti-Mouse IgG (H+L), HRP	Cell Signaling Technology	7076S
Anti-Rabbit IgG (H+L), HRP	Invitrogen	SA1-200
Donkey anti-Mouse IgG (H+L) Alexa Fluor 488	Abcam	ab150109
Donkey anti-Mouse IgG (H+L) Alexa Fluor 647	Abcam	ab150135
