# Supplementary material for: A single small molecule-based human embryo model reveals V-ATPase requirement in mammalian blastocyst cavitation
Source: Cell Res. 2026 Apr 6;36(7):475–98. doi: 10.1038/s41422-026-01239-3 (PMC13287814; doi:10.1038/s41422-026-01239-3)
Supplement: Supplementary file 22 — Supplementary information, Table S2 [file 41422_2026_1239_MOESM22_ESM.pdf]

Table S2. Oligos and siRNAs used in this study.

Gene name	Forward sequence 5'-3'	Reverse sequence 5'-3'
**qPCR oligos used in this study**		
ZIC2	GCGCAACTCCACAACCAGTA	TGCCGCATATAGCGGAAAAAG
KLF17	GCTGCCCAGGATAACGAGAAC	ATCTCTGCGCTGTGAGGAAAG
TACSTD2	ACAACGATGGCCTCTACGAC	GTCCAGGTCTGAGTGGTTGAA
NANOG	TTTGTGGGCCTGAAGAAAACT	AGGGCTGTCCTGAATAAGCAG
CLDN4	TGGGGCTACAGGTAATGGG	GGTCTGCGAGGTGACAATGTT
CDX2	GGAACCTGTGCGAGTGGAT	TCCGTGTACACCACTCGATATT
KRT19	CCGCGACTACAGCCACTACT	GTCGATCTGCAGGACAATCC
ATP6V0A4	CTGCCGAGGAAACGTGTACTT	GGCTCGAAACCCATCACAGA
ATP6V1B1	GGCGGTCACCCGAAACTAC	GGACGATCTCCGCATACTGG
FUCA2	TGGTTCTGGTGGTATTGGCAA	CCCCACAAGGTAAAGCCTTCA
CTSZ	CAGCGGATCTGCCCAAGAG	CGATGACGTTCTGCACGGA
ACP2	GTTACCTTGCTGTACCGCCAT	CCCTGGGGCCATTCTTCTTC
CTSV	CGTGACGCCAGTGAAGAATCA	CGCTCAGTGAGACAAGTTTCC
SMPD1	CTGTCTGACTCTCGGGTTCTC	CTATGCGATGTAACCTGGCAG
FOLR1	GCTCAGCGGATGACAACACA	CCTGGCCCATGCAATCCTT
**Mouse embryos qPCR**		
Atp6v0a4	TGGAGGCTGCGTATTGCTG	CCTTCGGACTTCATTCACAAACT
Atp6v0b	AGTTGCTCTACCTCGGGATCT	ATGCCACATCAAAGCGAAAGC
**siRNAs used in mouse embryo experiments**		
siNC	UUCUCCGAACGUGUCACGUTT	ACGUGACACGUUCGGAGAATT
si*Atp6v0a4*	GGGUCAUCAAAGUACAGAATT	UUCUGUACUUUGAUGACCCTT
si*Atp6v0b*	GCAUCAUCAUGGCAAUUGUTT	ACAAUUGCCAUGAUGAUGCTT
